# Supplementary material for: Splitting schizophrenia: divergent cognitive and educational outcomes revealed by genomic structural equation modelling
Source: Mol Psychiatry. 2026 Jan 31;31(6):3098–107. doi: 10.1038/s41380-026-03444-3 (PMC13190233; doi:10.1038/s41380-026-03444-3)
Supplement: Supplementary file 2 — Supplemental table 1 [file 41380_2026_3444_MOESM2_ESM.pdf]

| GWAS Trait | Author | Observed h2 | Observed h2 SE | h2 z | mean chi-square | LDScore intercept | Number of independent hits | Sample size (NEFF if binary trait) |
|---|---|---|---|---|---|---|---|---|
| **Schizophrenia (EUR)** | Trubetskoy et al 202 | 0.403 | 0.014 | 28.2 | 2.05 | 1.08 | 313 EUR and Asian | 117,498 |
| **Bipolar disorder** | Mullins et al 2021 | 0.284 | 0.012 | 24.1 | 1.61 | 1.03 | 64 | 101,962 |
| **Educational attainme** | Lee et al 2018 | 0.112 | 0.003 | 37.2 | 2.65 | 0.98 | 1,271 | 766,345 |
| **IQ** | Savage et al 2018 | 0.188 | 0.007 | 27.3 | 2.04 | 1.03 | 242 | 269,867 |
| **SZspecific** | Current study | 0.305 | 0.014 | 21.6 | 1.41 | 1.01 | 63 | 65,626 |
| **PSYshared** | Current study | 0.406 | 0.015 | 26.5 | 1.55 | 0.99 | 78 | 71,376 |

*Summary statistics of SZspecific and PSYshared were created through GWAS-by-subtraction. h2, SE, mean chi-square and LDScore intercept were computed with the LDSC software contained within the R package GenomicSEM. Number of independent hits was calculated as reported in the methods. The Sample size is the sample size reported by Lee et al., 2018 for EA and Savage et al., 2018 for IQ. NEFF were provided in the summary statistics for Trubetskoy et al., 2022 and Mullins et al., 2021. NEFF was calculated as described in Demange et al., 2021 for SZspecific and PSYshared.*
