# Supplementary material for: Splitting schizophrenia: divergent cognitive and educational outcomes revealed by genomic structural equation modelling
Source: Mol Psychiatry. 2026 Jan 31;31(6):3098–107. doi: 10.1038/s41380-026-03444-3 (PMC13190233; doi:10.1038/s41380-026-03444-3)
Supplement: Supplementary file 3 — Supplemental table 2 [file 41380_2026_3444_MOESM3_ESM.pdf]

Results of GWAS by subtraction model						
lhs	rhs	Estimate (Unstandardised)	SE (Unstandardised)	Standardised Genotype	SE (Standardised)	P-value
PSYshared	Schizophrenia	0.296	0.010	0.679	0.024	5.00E-183
PSYshared	Bipolar disorder	0.432	0.009	1.000	0.021	<5E-300
SZspecific	Schizophrenia	0.321	0.009	0.734	0.021	4.68E-275
