# Supplementary material for: Splitting schizophrenia: divergent cognitive and educational outcomes revealed by genomic structural equation modelling
Source: Mol Psychiatry. 2026 Jan 31;31(6):3098–107. doi: 10.1038/s41380-026-03444-3 (PMC13190233; doi:10.1038/s41380-026-03444-3)
Supplement: Supplementary file 4 — Supplemental table 3 [file 41380_2026_3444_MOESM4_ESM.pdf]

Association results for the 63 independent SNPs that reached genome-wide significance (P < 5x10-8) in GWAS of SZspecific

SNP	CHR	BP	A1	A2	MAF	beta	SE	Z	P	SZ GWAS (2022) P	BIP (2021) P	GWAS catalog associations
rs6688934	1	2372397	A	G	0 4920	-0 0999	0 0181	-5 5256	3 28E-08	8 08E-12	0 5576	Nil reported
rs380582	1	6796264	T	C	0 4533	-0 1000	0 0171	-5 8363	5 34E-09	5 51E-04	1 60E-05	Nil reported
rs4950119	1	98491248	A	C	0 2078	-0 1625	0 0216	-7 5396	4 71E-14	8 77E-21	0 2913	Schizophrenia
rs12071594	1	173815111	T	C	0 3360	0 1054	0 0181	5 8245	5 73E-09	5 06E-06	0 004593	Nil reported
rs16851037	1	177271313	G	A	0 2157	-0 1192	0 0211	-5 6482	1 62E-08	4 87E-12	0 5494	Nil reported
rs61833239	1	244010441	T	G	0 1839	-0 1329	0 0243	-5 4643	4 65E-08	2 80E-12	0 3268	Schizophrenia
rs1822616	2	55283878	T	G	0 4990	-0 0950	0 0171	-5 5396	3 03E-08	1 55E-04	4 95E-04	Nil reported
rs1518393	2	58171220	A	C	0 4066	-0 1061	0 0177	-5 9897	2 10E-09	1 87E-15	0 1596	Smoking
rs778370	2	233743363	C	T	0 3251	-0 1013	0 0183	-5 5238	3 32E-08	6 05E-17	0 007424	Nil reported
rs1463221	3	17221017	T	C	0 4404	0 1073	0 0171	6 2590	3 87E-10	5 32E-08	0 01994	Nil reported
rs9881850	3	17554181	C	T	0 4384	0 1048	0 0171	6 1170	9 54E-10	2 07E-08	0 05719	Nil reported
rs17273111	3	17855181	G	A	0 4811	-0 1109	0 0169	-6 5448	5 96E-11	1 16E-09	0 05848	Nil reported
rs1278493	3	135814009	G	A	0 4692	-0 1036	0 0173	-5 9790	2 25E-09	9 52E-10	0 337	BMI, neuroticism, schziophrenia vs bipolar, AF
rs10935184	3	136153468	T	C	0 4473	0 0970	0 0171	5 6577	1 53E-08	1 61E-10	0 9285	Schizophrenia, neuroticism
rs35746395	3	181245320	G	A	0 1153	0 1679	0 0292	5 7475	9 06E-09	6 79E-11	0 9305	Nil reported
rs13107325	4	103188709	C	T	0 0795	-0 2150	0 0338	-6 3591	2 03E-10	2 90E-21	0 0121	>100 assocations (https://www.ebi.ac.uk/gwas/variants/rs13107325) , including schizophrenia and intelligence
rs11740474	5	153680747	A	T	0 4115	-0 0963	0 0173	-5 5540	2 79E-08	1 13E-09	0 7213	Schizophrenia, cognitive ability, educational attainment and ASD
rs34158769	6	26336572	G	A	0 0666	0 1896	0 0334	5 6754	1 38E-08	9 51E-29	1 41E-10	Hip circumference, lung cancer
rs66488313	6	26631568	G	T	0 0686	0 2018	0 0321	6 2794	3 40E-10	8 98E-36	9 28E-14	Lung cancer
rs66462181	6	27091661	T	C	0 0686	0 2023	0 0320	6 3215	2 59E-10	4 26E-36	8 60E-14	Educational attainment
rs13196692	6	27379119	C	T	0 0706	0 2215	0 0316	7 0074	2 43E-12	1 42E-37	1 40E-11	Nil reported
rs13217620	6	27653120	C	T	0 0606	0 2347	0 0325	7 2133	5 46E-13	4 76E-39	9 34E-12	Intelligence, inguinal hernia, lung cancer and smoking
rs13197574	6	28060239	T	C	0 0726	0 2176	0 0312	6 9673	3 23E-12	3 83E-38	4 05E-12	Depression, sarcoidosis, streptococcus seropositivity and intelligence
rs758398	6	28320674	C	T	0 2734	0 1457	0 0195	7 4735	7 81E-14	1 60E-20	0 3615	Nil reported
rs1233578	6	28712247	A	G	0 1511	0 2098	0 0255	8 2297	1 88E-16	3 22E-36	2 42E-05	Intelligence, asthma, PTSD, streptococcus seropositivity, COPD, smoking, BMI, ADHD, ASD
rs9257566	6	29144532	C	T	0 0915	0 2288	0 0291	7 8758	3 38E-15	1 27E-36	3 54E-07	Schizophrenia, breast cancer, streptococcus seropositivity
rs2206853	6	29437033	C	T	0 0746	0 1975	0 0308	6 4135	1 42E-10	1 74E-31	7 90E-10	Serum urate
rs2523761	6	29818726	A	G	0 1332	0 1684	0 0237	7 0944	1 30E-12	3 28E-23	0 01264	General cognitive ability
rs1117487	6	30170970	A	G	0 1829	0 1316	0 0225	5 8602	4 62E-09	1 05E-24	9 04E-07	Nil reported
rs3130662	6	30713808	C	A	0 1352	0 1540	0 0248	6 2023	5 56E-10	6 84E-27	3 89E-07	Sarcoidosis and Gastro-esophageal reflux
rs1793894	6	31196862	G	A	0 1024	0 1425	0 0259	5 5010	3 78E-08	1 31E-26	3 04E-09	Staphylococcus and streptococcus positivity, inguinal hernia
rs3094011	6	31451836	T	C	0 0895	0 1620	0 0269	6 0204	1 74E-09	1 87E-25	7 41E-07	Inguinal hernia
rs67682613	6	31826705	G	A	0 1133	0 1576	0 0254	6 2065	5 42E-10	2 57E-27	3 07E-06	Schizophrenia, lung cancer
rs9267898	6	32202935	T	C	0 4066	0 1070	0 0179	5 9715	2 35E-09	9 41E-07	0 009191	Complement C4 levels
rs9267922	6	32206465	G	A	0 2217	0 1613	0 0216	7 4650	8 33E-14	9 49E-20	0 4373	Nil reported
rs9274623	6	32635998	G	T	0 2028	0 1742	0 0253	6 8840	5 82E-12	1 13E-23	0 002908	Schizophrenia, transmembrane proteins
rs1480380	6	32913246	C	T	0 0775	0 1790	0 0315	5 6778	1 36E-08	4 85E-22	2 94E-05	Psychiatric pleiotropy, streptococcus, sarcoid, glomerulonephritis
rs2097942	7	104725105	G	A	0 4960	-0 1048	0 0169	-6 1862	6 16E-10	1 89E-11	0 6117	Nil reported
rs2040915	7	104979558	G	A	0 4751	0 0973	0 0170	5 7203	1 06E-08	2 35E-09	0 4179	Nil reported
rs37658	7	110999503	A	G	0 4811	0 1100	0 0174	6 3314	2 43E-10	2 48E-10	0 1711	Reaction time and smoking
rs10046758	8	4184170	C	G	0 2197	-0 1155	0 0209	-5 5245	3 30E-08	1 38E-08	0 3829	Schizophrenia
rs7838316	8	27425314	G	A	0 1551	0 1538	0 0237	6 4782	9 28E-11	3 72E-12	0 5084	Smoking initiation
rs7856690	9	37127545	A	T	0 3539	-0 1012	0 0178	-5 6956	1 23E-08	2 15E-07	0 07462	BMI, mean arterial pressure
rs605765	11	30217503	C	T	0 3827	-0 0960	0 0175	-5 4771	4 32E-08	1 16E-06	0 05195	Schizophrenia, morning chronotype
rs1783976	11	57462752	T	C	0 4394	-0 1086	0 0171	-6 3339	2 39E-10	3 28E-08	0 01863	Brain morphology, height
rs118031494	11	133821144	G	A	0 0775	0 2109	0 0329	6 4178	1 38E-10	1 15E-08	0 02606	Nil reported
rs61937595	12	57682956	C	T	0 0905	0 2138	0 0316	6 7600	1 38E-11	1 15E-15	0 6321	Schizophrenia, neuroticism, cognitive function
rs3764002	12	108618630	C	T	0 2594	-0 1151	0 0194	-5 9414	2 83E-09	3 34E-05	3 30E-04	Schizophrenia, BMI, T2DM, adipose, risk taking, vital capacity, anxiety, triglycerides, AST, ALT, sex hormones, osteoarthritis, neuroticism, cognitive function
rs4766428	12	110723245	C	T	0 4533	-0 1347	0 0175	-7 6889	1 48E-14	3 93E-17	0 6004	Schizophrenia, psychiatric disorder pleiotropy, cognitive ability
rs4766500	12	110993791	G	A	0 3638	-0 1364	0 0177	-7 6983	1 38E-14	8 24E-12	0 008453	Body mass
rs12877581	13	74325499	G	C	0 3072	-0 1105	0 0195	-5 6666	1 46E-08	1 80E-09	0 5197	Schizophrenia
rs7048	15	43620073	A	C	0 1093	0 1319	0 0220	5 9847	2 17E-09	1 47E-06	0 003645	Nil reported
rs62039173	16	4463846	G	A	0 3141	0 1038	0 0189	5 5068	3 65E-08	6 30E-06	0 0147	Nil reported
rs56328224	17	43572419	C	T	0 2396	0 1249	0 0216	5 7785	7 54E-09	1 33E-08	0 2086	Medial temporal lobe thickness, covid-19 symptoms
rs62055887	17	43838710	T	C	0 2396	0 1010	0 0175	5 7716	7 85E-09	6 46E-09	0 0749	Nil reported
rs62062288	17	44096553	G	A	0 2366	0 1082	0 0177	6 1226	9 21E-10	1 65E-09	0 05969	Worrying, neurotic disorder, guilt, worry, risk taking, speeding, alcohol use, cortical surface area, brain regions: OFC, dlPFC, STG, motor premotor area
rs2532329	17	44350090	G	A	0 2515	0 1392	0 0218	6 3987	1 57E-10	2 61E-09	0 0637	Nil reported
rs117300236	17	44753350	A	G	0 2843	-0 1251	0 0210	-5 9655	2 44E-09	4 45E-08	0 06363	Nerve thickness
rs28758902	18	53408187	C	T	0 4553	-0 0986	0 0170	-5 7976	6 73E-09	2 57E-14	0 187	BMI, ASD, schizophrenia
rs71367545	18	77576337	G	A	0 1610	-0 1272	0 0212	-5 9900	2 10E-09	1 40E-12	0 75	Smoking
rs7245983	19	19657632	A	C	0 3251	-0 1011	0 0178	-5 6884	1 28E-08	3 09E-16	0 02719	Red blood cell width, prostate cancer
rs4814411	20	1641146	T	C	0 2525	0 1102	0 0195	5 6504	1 60E-08	1 72E-06	0 02404	Neurofibrillary tangles measurement
rs763263	22	42315790	C	T	0 2107	0 1244	0 0210	5 9325	2 98E-09	1 65E-10	0 5834	Nil reported

PTSD = post-traumatic stress disorder; COPD = chronic obstructive pulmonary disease; BMI = body mass index; ADHD = attention deficit hyperactivity disorder; ASD = autism spectrum disorder; OFC = orbitofrontal cortex; dlPFC = dorsolateral prefrontal cortex; STG = superior temportal gyrus;
T2DM = type 2 diabetes mellitus; AST = aspartate aminotransferase; ALT = alanine aminotransferase, AF = atrial fibrilliation. Note that SZ GWAS (2022) P-values derived from summary statistics for European ancestry as per the GenomicSEM procedure, but GWAS catalog has all ancestries.
