# Supplementary material for: Splitting schizophrenia: divergent cognitive and educational outcomes revealed by genomic structural equation modelling
Source: Mol Psychiatry. 2026 Jan 31;31(6):3098–107. doi: 10.1038/s41380-026-03444-3 (PMC13190233; doi:10.1038/s41380-026-03444-3)
Supplement: Supplementary file 5 — Supplemental table 4 [file 41380_2026_3444_MOESM5_ESM.pdf]

**Association results for the 78 independent SNPs that reached genome-wide significance (P < 5x10-8) in GWAS of PSYshared**

| SNP | CHR | BP | A1 | A2 | MAF | beta | SE | Z | P | SZ GWAS (2022) P | BIP GWAS (2021) P | GWAS catalog associations |
|---|---|---|---|---|---|---|---|---|---|---|---|---|
| rs17693963 | 6 | 27710165 | A | C | 0 0865 | 0 1546 | 0 0209 | 7 4121 | 1 24E-13 | 3 49E-38 | 5 84E-14 | Autism spectrum disorder, schizophrenia, bipolar, schizoaffective, pain |
| rs13200214 | 6 | 28017250 | C | T | 0 0726 | 0 1496 | 0 0218 | 6 8691 | 6 46E-12 | 6 22E-38 | 3 17E-12 | **Novel** |
| rs13194504 | 6 | 28630691 | G | A | 0 0686 | 0 1519 | 0 0219 | 6 9336 | 4 10E-12 | 9 63E-38 | 2 20E-12 | Depression, inguinal hernia |
| rs13198809 | 6 | 28323702 | G | A | 0 0686 | 0 1491 | 0 0218 | 6 8459 | 7 60E-12 | 1 01E-37 | 3 38E-12 | Anxiety, depression |
| rs13207082 | 6 | 27251379 | A | G | 0 0686 | 0 1565 | 0 0223 | 7 0184 | 2 24E-12 | 1 26E-36 | 8 63E-13 | Breast cancer, lung cancer, vital capacity, haemoglobin, forced expiratory volume |
| rs13195402 | 6 | 26463575 | G | T | 0 0686 | 0 1741 | 0 0227 | 7 6736 | 1 67E-14 | 5 74E-36 | 5 75E-15 | Mastocytosis, schizophrenia, breast cancer, inguinal hernia, bipolar |
| rs66975207 | 6 | 26942146 | A | C | 0 0686 | 0 1667 | 0 0222 | 7 5197 | 5 49E-14 | 1 89E-35 | 2 36E-14 | Depression, streptococcus seropositivity, urate |
| rs3117145 | 6 | 29046418 | A | T | 0 0696 | 0 1534 | 0 0231 | 6 6490 | 2 95E-11 | 1 12E-33 | 1 54E-11 | **Novel** |
| rs1233393 | 6 | 29549653 | C | G | 0 0716 | 0 1439 | 0 0213 | 6 7679 | 1 31E-11 | 2 44E-31 | 5 76E-12 | Depression, chronic obstructive pulmonary disease, smoking, brain morphology, triglycerides |
| rs17598658 | 6 | 26175866 | A | C | 0 0656 | 0 1601 | 0 0232 | 6 8983 | 5 26E-12 | 3 08E-29 | 2 78E-12 | Cognitive aspects of educational attainment, breast cancer |
| rs1264349 | 6 | 30796659 | A | G | 0 0805 | 0 1281 | 0 0197 | 6 4996 | 8 05E-11 | 2 19E-26 | 4 30E-11 | Inguinal hernia, urinary tract infection |
| rs3130247 | 6 | 30515043 | T | C | 0 0785 | 0 1246 | 0 0200 | 6 2426 | 4 30E-10 | 4 10E-26 | 2 22E-10 | Streptococcus seropositivity, haemoglobin |
| rs532086 | 6 | 31881309 | T | C | 0 1074 | 0 1172 | 0 0180 | 6 5060 | 7 72E-11 | 1 78E-25 | 3 83E-11 | Inguinal hernia, glomerular filtration, BMI, staphylococcus and streptococcus seropositivity |
| rs3095311 | 6 | 31051675 | A | G | 0 0855 | 0 1230 | 0 0191 | 6 4533 | 1 09E-10 | 1 61E-24 | 6 16E-11 | Inguinal hernia |
| rs12668848 | 7 | 2020995 | G | A | 0 4205 | 0 0728 | 0 0123 | 5 9109 | 3 40E-09 | 3 18E-24 | 1 90E-09 | Schizophrenia, OCD, 'psychiatric pleiotropy', bipolar |
| rs1541269 | 6 | 30103360 | G | A | 0 0964 | 0 1240 | 0 0189 | 6 5512 | 5 71E-11 | 1 53E-22 | 2 77E-11 | **Novel** |
| rs17526722 | 6 | 25918855 | G | A | 0 0646 | 0 1304 | 0 0235 | 5 5558 | 2 76E-08 | 3 13E-22 | 1 76E-08 | **Novel** |
| rs4702 | 15 | 91426560 | G | A | 0 4374 | 0 0731 | 0 0126 | 5 8188 | 5 93E-09 | 2 79E-21 | 3 52E-09 | Insomnia, risky sexual behaviour, bipolar, schizophrenia, intelligence, educational attainment, 'neuropsychiatric disorders', trauma, depression, lean mass'psychiatric pleiotropy' breast carcinoma, autism, ADHD, being emotionally hurt, age at sexual intercourse, cortical thickness, brain measurement, opioiduse, neuroticism, cannabis use, alcohol use, body height. |
| rs2442729 | 6 | 31319226 | C | T | 0 1372 | 0 1067 | 0 0175 | 6 1000 | 1 06E-09 | 4 99E-21 | 5 60E-10 | **Novel** |
| rs2159100 | 12 | 2346393 | C | T | 0 3161 | -0 0996 | 0 0127 | -7 8412 | 4 46E-15 | 1 19E-19 | 1 87E-15 | Schizophrenia, bipolar, anorexia |
| rs2577831 | 3 | 52628056 | C | A | 0 4861 | 0 0865 | 0 0120 | 7 1819 | 6 88E-13 | 2 31E-18 | 3 83E-13 | Schizophrenia, lean mass, brain morphology, erythrocyte count, serum gamma glutamyl transferase |
| rs7612624 | 3 | 52893426 | T | C | 0 4066 | -0 0854 | 0 0124 | -6 8654 | 6 63E-12 | 1 46E-15 | 3 31E-12 | **Novel** |
| rs7622851 | 3 | 52333671 | C | G | 0 4821 | 0 0802 | 0 0121 | 6 6526 | 2 88E-11 | 1 30E-14 | 1 83E-11 | BMI |
| rs2074473 | 6 | 30154199 | T | C | 0 4334 | 0 0737 | 0 0124 | 5 9221 | 3 18E-09 | 2 56E-12 | 1 60E-09 | **Novel** |
| rs748455 | 15 | 85149575 | T | C | 0 2903 | 0 0862 | 0 0133 | 6 4569 | 1 07E-10 | 1 50E-11 | 5 01E-11 | Schizophrenia, hypertrophic cardiomyopathy, bipolar |
| rs12672003 | 7 | 24647222 | A | G | 0 1084 | -0 1175 | 0 0201 | -5 8475 | 4 99E-09 | 7 46E-10 | 2 72E-09 | Bipolar |
| rs12902672 | 15 | 84716986 | G | A | 0 2724 | 0 0818 | 0 0136 | 6 0153 | 1 80E-09 | 1 97E-09 | 1 25E-09 | **Novel** |
| rs9834970 | 3 | 36856030 | T | C | 0 4891 | -0 1059 | 0 0120 | -8 7933 | 1 45E-18 | 2 15E-09 | 6 63E-19 | Bipolar, schizophrenia, autism, depression, adhd, ocd, Tourette's, anorexia, response to lithium, peptic ulcers |
| rs7201930 | 16 | 9958655 | T | C | 0 3380 | -0 0748 | 0 0135 | -5 5524 | 2 82E-08 | 8 53E-09 | 1 89E-08 | Body height |
| rs10737496 | 1 | 163745389 | C | T | 0 4801 | 0 0692 | 0 0122 | 5 6821 | 1 33E-08 | 1 34E-07 | 7 17E-09 | Bipolar |
| rs73560982 | 8 | 34214305 | T | C | 0 0676 | -0 1482 | 0 0259 | -5 7183 | 1 08E-08 | 3 91E-07 | 6 58E-09 | **Novel** |
| rs13417268 | 2 | 169481837 | C | G | 0 2306 | 0 0789 | 0 0143 | 5 5362 | 3 09E-08 | 1 08E-06 | 2 05E-08 | Bipolar, schizophrenia |
| rs2693698 | 14 | 99719219 | A | G | 0 4662 | -0 0677 | 0 0122 | -5 5560 | 2 76E-08 | 1 49E-06 | 1 96E-08 | Schizophrenia, 'psychiatric pleiotropy' and thalamic volume |
| rs67712855 | 20 | 43682551 | T | G | 0 3062 | 0 0864 | 0 0133 | 6 4763 | 9 40E-11 | 2 35E-06 | 4 22E-11 | Bipolar |
| rs12932628 | 16 | 89632725 | G | T | 0 4563 | -0 0728 | 0 0127 | -5 7318 | 9 94E-09 | 5 18E-06 | 6 71E-09 | Bipolar |
| rs472913 | 1 | 61095558 | G | C | 0 4274 | -0 0718 | 0 0121 | -5 9549 | 2 60E-09 | 5 71E-06 | 1 89E-09 | Bipolar |
| rs10255167 | 7 | 140676153 | G | A | 0 2177 | -0 0848 | 0 0153 | -5 5446 | 2 95E-08 | 8 17E-06 | 1 60E-08 | Bipolar |
| rs115694474 | 3 | 70488788 | T | A | 0 1789 | 0 0845 | 0 0154 | 5 4819 | 4 21E-08 | 1 30E-05 | 2 35E-08 | Bipolar |
| rs3088186 | 8 | 10226355 | C | T | 0 2783 | -0 0724 | 0 0131 | -5 5319 | 3 17E-08 | 1 38E-05 | 2 08E-08 | Schizophrenia, bipolar |
| rs11764361 | 7 | 105043229 | A | G | 0 3022 | 0 0785 | 0 0135 | 5 8271 | 5 64E-09 | 1 79E-05 | 3 47E-09 | Bipolar |
| rs5758064 | 22 | 41153879 | T | C | 0 4801 | 0 0669 | 0 0121 | 5 5526 | 2 81E-08 | 1 86E-05 | 2 01E-08 | Bipolar |
| rs6865469 | 5 | 78849505 | G | T | 0 2664 | -0 0743 | 0 0133 | -5 5684 | 2 57E-08 | 4 17E-05 | 1 65E-08 | Bipolar |
| rs72809838 | 2 | 97428320 | G | A | 0 2952 | 0 0845 | 0 0131 | 6 4589 | 1 05E-10 | 8 82E-05 | 6 06E-11 | **Novel** |
| rs11856299 | 15 | 83534421 | C | T | 0 2197 | 0 0794 | 0 0143 | 5 5727 | 2 51E-08 | 1 10E-04 | 1 53E-08 | **Novel** |
| rs1487445 | 6 | 98565211 | C | T | 0 4930 | -0 0947 | 0 0120 | -7 8617 | 3 79E-15 | 2 29E-04 | 1 48E-15 | Educational attainement, mathematical ability, cortical thickness, brain measurement, bipolar, risky sexual behaviour, serum alanine aminotransferase |
| rs61847719 | 10 | 62066170 | T | G | 0 0626 | -0 1597 | 0 0261 | -6 1312 | 8 72E-10 | 4 42E-04 | 4 53E-10 | **Novel** |
| rs62581014 | 9 | 141066490 | C | T | 0 3807 | -0 0832 | 0 0152 | -5 4910 | 4 00E-08 | 4 92E-04 | 2 77E-08 | Bipolar |
| rs6954854 | 7 | 21492589 | G | A | 0 4284 | 0 0744 | 0 0122 | 6 1117 | 9 86E-10 | 6 16E-04 | 5 94E-10 | Bipolar |
| rs4672 | 11 | 64009879 | G | A | 0 0736 | -0 1299 | 0 0223 | -5 8258 | 5 69E-09 | 9 50E-04 | 3 42E-09 | Cognitive performance, bipolar |
| rs112481526 | 4 | 123076007 | A | G | 0 2883 | -0 0806 | 0 0136 | -5 9221 | 3 18E-09 | 9 80E-04 | 1 86E-09 | Bipolar |
| rs10994415 | 10 | 62322034 | T | C | 0 0736 | -0 1508 | 0 0226 | -6 6867 | 2 28E-11 | 0 001014 | 1 14E-11 | Bipolar |
| rs17388568 | 4 | 123329362 | G | A | 0 2922 | -0 0747 | 0 0133 | -5 5969 | 2 18E-08 | 0 001492 | 1 39E-08 | Type 1 diabetes, ulcerative colits, allergic disease, eosinophil |
| rs10455979 | 6 | 166995260 | C | G | 0 4602 | -0 0710 | 0 0123 | -5 7673 | 8 05E-09 | 0 001721 | 4 22E-09 | Bipolar |
| rs17183814 | 2 | 166152389 | G | A | 0 0567 | 0 1314 | 0 0240 | 5 4798 | 4 26E-08 | 0 002195 | 2 68E-08 | Bipolar, ADHD, educational attainment |
| rs237460 | 20 | 48033127 | C | T | 0 4324 | -0 0706 | 0 0122 | -5 7977 | 6 72E-09 | 0 002226 | 4 25E-09 | Bipolar |
| rs4331993 | 6 | 152793572 | T | A | 0 3698 | -0 0699 | 0 0126 | -5 5572 | 2 74E-08 | 0 002537 | 1 98E-08 | Bipolar |
| rs61554907 | 17 | 38220432 | G | T | 0 1074 | -0 1109 | 0 0200 | -5 5541 | 2 79E-08 | 0 00281 | 1 64E-08 | Bipolar |
| rs113779084 | 7 | 11871787 | G | A | 0 2883 | -0 0964 | 0 0132 | -7 2942 | 3 00E-13 | 0 003394 | 1 42E-13 | Bipolar, educational attainment |
| rs28455634 | 16 | 9230816 | G | A | 0 3966 | 0 0802 | 0 0128 | 6 2509 | 4 08E-10 | 0 005266 | 2 63E-10 | Bipolar |
| rs678397 | 11 | 66324583 | T | C | 0 4503 | 0 0696 | 0 0121 | 5 7750 | 7 70E-09 | 0 005464 | 5 46E-09 | Bipolar |
| rs62489493 | 8 | 9763581 | C | G | 0 1262 | -0 1145 | 0 0175 | -6 5471 | 5 87E-11 | 0 008255 | 2 64E-11 | Bipolar |
| rs6032110 | 20 | 43944323 | A | G | 0 4920 | 0 0733 | 0 0122 | 6 0179 | 1 77E-09 | 0 01303 | 1 01E-09 | Bipolar |
| rs174592 | 11 | 61618608 | A | G | 0 3867 | -0 0919 | 0 0126 | -7 3149 | 2 57E-13 | 0 01584 | 9 92E-14 | Balding, cholesterol, 'psychiatric pleiotropy', triglycerides, bipolar, multiple metabolite levels, platelets, |
| rs12289486 | 11 | 79092527 | C | T | 0 1054 | -0 1055 | 0 0193 | -5 4628 | 4 69E-08 | 0 04813 | 3 30E-08 | Bipolar |
| rs475805 | 11 | 65848738 | G | A | 0 2266 | -0 0856 | 0 0145 | -5 8950 | 3 75E-09 | 0 06589 | 1 99E-09 | Bipolar, serum gamma glutamyl transferase |
| rs427037 | 6 | 32212264 | A | G | 0 2346 | -0 0928 | 0 0150 | -6 1763 | 6 56E-10 | 0 06918 | 3 54E-10 | Rheumatoid arthritis, irritable bowel syndrome |
| rs11870683 | 17 | 38129841 | T | A | 0 3380 | 0 0732 | 0 0133 | 5 4822 | 4 20E-08 | 0 08545 | 2 79E-08 | Bipolar |
| rs12575685 | 11 | 70517927 | G | A | 0 2913 | -0 0833 | 0 0131 | -6 3619 | 1 99E-10 | 0 1038 | 1 24E-10 | Bipolar |
| rs28565152 | 5 | 7542911 | G | A | 0 2187 | -0 0857 | 0 0145 | -5 9044 | 3 54E-09 | 0 1376 | 1 96E-09 | Depression, bipolar |
| rs6887473 | 5 | 80961069 | G | A | 0 2674 | 0 0770 | 0 0136 | 5 6602 | 1 51E-08 | 0 2455 | 8 81E-09 | Bipolar |
| rs12868163 | 13 | 113878441 | C | T | 0 2316 | 0 0835 | 0 0144 | 5 8070 | 6 36E-09 | 0 2577 | 4 20E-09 | **Novel** |
| rs13044225 | 20 | 60865815 | A | G | 0 4523 | -0 0699 | 0 0123 | -5 6746 | 1 39E-08 | 0 2932 | 8 50E-09 | Platelet volume, bipolar |
| rs4447398 | 15 | 42904904 | A | C | 0 1084 | 0 1050 | 0 0179 | 5 8697 | 4 37E-09 | 0 3577 | 2 61E-09 | Bipolar, depression |
| rs72841199 | 5 | 169284998 | A | G | 0 4334 | 0 0799 | 0 0122 | 6 5632 | 5 27E-11 | 0 4229 | 2 90E-11 | **Novel** |
| rs6584972 | 10 | 111901567 | G | A | 0 1889 | -0 1000 | 0 0165 | -6 0758 | 1 23E-09 | 0 4666 | 6 55E-10 | **Novel** |
| rs228768 | 17 | 42191893 | G | T | 0 2992 | 0 0822 | 0 0132 | 6 2226 | 4 89E-10 | 0 503 | 2 83E-10 | Bipolar |
| rs2273738 | 10 | 111648659 | C | T | 0 1561 | -0 1171 | 0 0176 | -6 6447 | 3 04E-11 | 0 5737 | 1 63E-11 | Bipolar |
| rs6992333 | 8 | 144993377 | A | G | 0 4324 | -0 0769 | 0 0130 | -5 9327 | 2 98E-09 | 0 9012 | 1 62E-09 | Platelet count, whole brain water diffusivity, bipolar disorder, brain connectivity |
