# Supplementary material for: Splitting schizophrenia: divergent cognitive and educational outcomes revealed by genomic structural equation modelling
Source: Mol Psychiatry. 2026 Jan 31;31(6):3098–107. doi: 10.1038/s41380-026-03444-3 (PMC13190233; doi:10.1038/s41380-026-03444-3)
Supplement: Supplementary file 6 — Supplemental table 5 [file 41380_2026_3444_MOESM6_ESM.pdf]

Comparison	Correlation	StandardError	ZScore	PValue	PValueFDR
SZ vs EA	0.01	0.019	0.74	0.46	0.46
SZ vs IQ	-0.22	0.022	-9.79	1.2E-22	3.0E-22
SZ vs PSYshared	0.68	0.028	24.20	2.1E-129	1.6E-128
SZ vs SZsp	0.83	0.038	22.02	1.7E-107	5.1E-107
BD vs EA	0.11	0.019	5.92	3.3E-09	4.9E-09
BD vs IQ	-0.07	0.022	-3.36	7.9E-04	9.1E-04
BD vs PSYshared	0.99	0.041	24.11	2.0E-128	9.9E-128
BD vs SZ	0.68	0.030	22.47	8.1E-112	3.0E-111
BD vs SZsp	0.14	0.031	4.57	4.8E-06	6.5E-06
SZsp vs EA	-0.06	0.023	-2.45	1.4E-02	1.5E-02
SZsp vs IQ	-0.24	0.027	-9.06	1.3E-19	2.8E-19
PSYshared vs EA	0.11	0.018	5.97	2.3E-09	3.9E-09
PSYshared vs IQ	-0.07	0.019	-3.74	1.8E-04	2.3E-04
PSYshared vs SZsp	0.17	0.027	6.04	1.5E-09	2.8E-09
EA vs IQ	0.73	0.026	28.08	1.9E-173	2.8E-172
