# Supplementary material for: Splitting schizophrenia: divergent cognitive and educational outcomes revealed by genomic structural equation modelling
Source: Mol Psychiatry. 2026 Jan 31;31(6):3098–107. doi: 10.1038/s41380-026-03444-3 (PMC13190233; doi:10.1038/s41380-026-03444-3)
Supplement: Supplementary file 7 — Supplemental table 6 [file 41380_2026_3444_MOESM7_ESM.pdf]

	N	Age Mean (SD)	% female	Years in Education, Mean (SD)
**Whole cohort**	381,688	56.8 (8)	54.0	14.0 (5)
**MHQ cohort**	125,063	56.0 (8)	56.2	15.7 (5)

	N	Age Mean (SD)	% female	Fluid Intelligence Mean (SD)
**Whole cohort**	47,307	55.7 (7.6)	51.1	6.7 (2.1)
