# Supplementary material for: Splitting schizophrenia: divergent cognitive and educational outcomes revealed by genomic structural equation modelling
Source: Mol Psychiatry. 2026 Jan 31;31(6):3098–107. doi: 10.1038/s41380-026-03444-3 (PMC13190233; doi:10.1038/s41380-026-03444-3)
Supplement: Supplementary file 8 — Supplemental table 7 [file 41380_2026_3444_MOESM8_ESM.pdf]

| PGS | Population | Outcome | N | Regression type | Beta | SE | Lower CI (95%) | Upper CI (95%) | P-value | Model Covariates |
|---|---|---|---|---|---|---|---|---|---|---|
| SZ | Whole UKB | Education years | 381,688 | Linear | -0.00215 | 0.00778 | -0.01741 | 0.01311 | 0.782 | year of birth, sex, PCs 1-6 |
| SZ | MHQ | Education years | 125,063 | Linear | 0.03079 | 0.01269 | 0.0059176 | 0.0556624 | 0.01525 | year of birth, sex, PCs 1-6 |
| Bipolar | Whole UKB | Education years | 381,688 | Linear | 0.14250 | 0.00787 | 0.12708 | 0.15792 | < 2e-16 | year of birth, sex, PCs 1-6 |
| Bipolar | MHQ | Education years | 125,063 | Linear | 0.1268 | 0.01281 | 0.1016924 | 0.1519076 | < 2e-16 | year of birth, sex, PCs 1-6 |
| SZspecific | Whole UKB | Education years | 381,688 | Linear | -0.12880 | 0.00843 | -0.14531 | -0.11229 | < 2e-16 | year of birth, sex, PCs 1-6 |
| SZspecific | MHQ | Education years | 125,063 | Linear | -0.08189 | 0.01369 | -0.1087224 | -0.0550576 | 2.23E-09 | year of birth, sex, PCs 1-6 |
| PSYshared | Whole UKB | Education years | 381,688 | Linear | 0.13780 | 0.00794 | 0.12223 | 0.15337 | < 2e-16 | year of birth, sex, PCs 1-6 |
| PSYshared | MHQ | Education years | 125,063 | Linear | 0.1262 | 0.01292 | 0.1008768 | 0.1515232 | < 2e-16 | year of birth, sex, PCs 1-6 |
| SZ | Whole UKB | Fluid intelligence | 47,307 | Linear | -0.164902 | 0.009371 | -0.1832692 | -0.14653484 | < 2e-16 | year of birth, sex, PCs 1-6 |
| Bipolar | Whole UKB | Fluid intelligence | 47,307 | Linear | -0.070239 | 0.00942 | -0.0887022 | -0.0517758 | 9.07E-14 | year of birth, sex, PCs 1-6 |
| SZspecific | Whole UKB | Fluid intelligence | 47,307 | Linear | -0.094919 | 0.010116 | -0.1147464 | -0.07509164 | < 2e-16 | year of birth, sex, PCs 1-6 |
| PSYshared | Whole UKB | Fluid intelligence | 47,307 | Linear | -0.075151 | 0.009525 | -0.09382 | -0.056482 | 3.10E-15 | year of birth, sex, PCs 1-6 |
