# Supplementary material for: Splitting schizophrenia: divergent cognitive and educational outcomes revealed by genomic structural equation modelling
Source: Mol Psychiatry. 2026 Jan 31;31(6):3098–107. doi: 10.1038/s41380-026-03444-3 (PMC13190233; doi:10.1038/s41380-026-03444-3)
Supplement: Supplementary file 9 — Supplemental table 8a [file 41380_2026_3444_MOESM9_ESM.pdf]

Supplementary Table 8a. MAGMA Gene-set analysis

Latent variable	Gene set	N genes	Beta	Beta STD	SE	P	Pbonferroni
**SZspecific**	STARK_PREFRONTAL_CORTEX_22Q11_DELETION_UP	186	0 3141	0 0311	0 0697	0 000003	0 0569
**SZspecific**	GOCC_L_TYPE_VOLTAGE_GATED_CALCIUM_CHANNEL_COMPLEX	12	1 2230	0 0309	0 3076	0 000035	0 5983
**SZspecific**	KEGG_CARDIAC_MUSCLE_CONTRACTION	68	0 4172	0 0251	0 1114	0 000091	1 0000
**SZspecific**	GOCC_DENDRITIC_TREE	572	0 1469	0 0252	0 0395	0 000102	1 0000
**SZspecific**	GOBP_CALCIUM_ION_TRANSMEMBRANE_TRANSPORT_VIA_HIGH_VOLTAGE_GATED_CALCIUM_CHANNEL	15	0 9704	0 0274	0 2645	0 000122	1 0000
**SZspecific**	GOCC_MEMBRANE_PROTEIN_COMPLEX	1105	0 1002	0 0236	0 0279	0 000163	1 0000
**SZspecific**	MIKKELSEN_DEDIFFERENTIATED_STATE_DN	7	1 3047	0 0252	0 3788	0 000287	1 0000
**SZspecific**	GOBP_POSITIVE_REGULATION_OF_SKELETAL_MUSCLE_CELL_DIFFERENTIATION	6	1 3763	0 0246	0 4120	0 000419	1 0000
**SZspecific**	REACTOME_THE_CITRIC_ACID_TCA_CYCLE_AND_RESPIRATORY_ELECTRON_TRANSPORT	157	0 2146	0 0195	0 0680	0 000797	1 0000
**SZspecific**	REACTOME_REDUCTION_OF_CYTOSOLIC_CA_LEVELS	11	0 9972	0 0241	0 3178	0 000853	1 0000
**PSYshared**	GOCC_SYNAPSE	1364	0 1426	0 0370	0 0274	9 32E-08	0 0016
**PSYshared**	GOBP_REGULATION_OF_TRANS_SYNAPTIC_SIGNALING	443	0 2384	0 0362	0 0465	1 49E-07	0 0025
**PSYshared**	GOBP_SYNAPTIC_SIGNALING	713	0 1871	0 0358	0 0371	2 35E-07	0 0040
**PSYshared**	GOMF_VOLTAGE_GATED_CALCIUM_CHANNEL_ACTIVITY_INVOLVED_IN_CARDIAC_MUSCLE_CELL_ACTION_PO	5	2 5472	0 0416	0 5162	4 05E-07	0 0069
**PSYshared**	GOBP_RESPONSE_TO_METAL_ION	348	0 2405	0 0324	0 0527	2 54E-06	0 0432
**PSYshared**	GOCC_NEURON_PROJECTION	1254	0 1307	0 0326	0 0289	3 03E-06	0 0516
**PSYshared**	GOBP_NEUROGENESIS	1562	0 1159	0 0320	0 0262	4 80E-06	0 0817
**PSYshared**	GOCC_DENDRITIC_TREE	572	0 1854	0 0319	0 0419	4 88E-06	0 0829
**PSYshared**	REACTOME_NEURONAL_SYSTEM	383	0 2248	0 0318	0 0523	8 73E-06	0 1483
**PSYshared**	GOBP_GENERATION_OF_NEURONS	1348	0 1175	0 0303	0 0281	1 51E-05	0 2561
