# Supplementary material for: Splitting schizophrenia: divergent cognitive and educational outcomes revealed by genomic structural equation modelling
Source: Mol Psychiatry. 2026 Jan 31;31(6):3098–107. doi: 10.1038/s41380-026-03444-3 (PMC13190233; doi:10.1038/s41380-026-03444-3)
Supplement: Supplementary file 10 — Supplemental table 8b [file 41380_2026_3444_MOESM10_ESM.pdf]

SZ-specific Top 100 Genes		
Gene	Chromosome	Gene Based P-value
ZSCAN31	6	9 10E-15
DPYD	1	7 80E-13
TBC1D5	3	1 54E-12
ZKSCAN3	6	1 80E-12
HIST1H4L	6	3 50E-12
PBX2	6	7 03E-12
ZKSCAN4	6	7 65E-12
HIST1H2BN	6	9 68E-12
ZSCAN12	6	2 45E-11
GPSM3	6	7 41E-11
SCAND3	6	7 97E-11
PGBD1	6	9 36E-11
RP11-691N7.6	11	1 03E-10
TMX2-CTNND1	11	1 06E-10
ZDHHC5	11	2 01E-10
ARL17B	17	2 23E-10
AGER	6	2 36E-10
CTNND1	11	3 29E-10
TMX2	11	3 78E-10
MAPT	17	3 89E-10
RNF5	6	4 86E-10
MSH5-SAPCD1	6	4 93E-10
HIST1H2BL	6	5 00E-10
MSH5	6	5 06E-10
NOTCH4	6	6 12E-10
KANSL1	17	6 30E-10
ZFP57	6	7 56E-10
STH	17	1 20E-09
CRHR1	17	1 21E-09
PRRC2A	6	1 27E-09
TRIM27	6	1 30E-09
PPT2	6	1 38E-09
HIST1H1B	6	1 73E-09
SPPL2C	17	1 95E-09
PPT2-EGFL8	6	2 08E-09
ZSCAN9	6	2 91E-09
OR2B2	6	3 96E-09
IFT81	12	4 62E-09
TCTN1	12	5 75E-09
LSM 2.00	6	8 13E-09
TCF4	18	8 43E-09

PSY-shared Top 100 Genes		
Gene	Chromosome	Gene Based P-value
TMEM258	11	2 05E-12
FADS2	11	2 23E-12
FADS1	11	3 26E-12
HIST1H4L	6	6 20E-12
BTN2A1	6	7 21E-12
HIST1H1B	6	1 32E-11
GLT8D1	3	1 48E-11
RP5-966M1.6	3	1 55E-11
HIST1H2BN	6	2 18E-11
CACNA1C	12	3 01E-11
ITIH4	3	3 34E-11
MYRF	11	5 04E-11
TMEM110-MUSTN1	3	6 65E-11
FAM196B	5	8 34E-11
TMEM110	3	8 40E-11
TRIM26	6	1 17E-10
GNL3	3	1 31E-10
NEK4	3	1 34E-10
PBRM1	3	1 92E-10
RP11-451M19.3	10	2 68E-10
SMIM4	3	3 82E-10
MSRA	8	4 44E-10
BTN3A2	6	5 55E-10
NMB	15	7 81E-10
STAB1	3	8 12E-10
ITIH3	3	1 02E-09
SFTA2	6	1 35E-09
ZNF592	15	1 54E-09
UBTF	17	2 29E-09
MUSTN1	3	2 71E-09
DOCK2	5	2 86E-09
NT5DC2	3	3 00E-09
ADD3	10	3 17E-09
WDR73	15	3 65E-09
HIST1H2AL	6	3 82E-09
TLR9	3	5 22E-09
RBPJL	20	5 39E-09
SFMBT1	3	5 76E-09
TRIM31	6	6 19E-09
RPS6KA2	6	8 42E-09
BCL11B	14	8 91E-09

WNT3	17	9 27E-09
OR5V1	6	9 28E-09
SRPK2	7	9 80E-09
HIST1H2AL	6	1 05E-08
PPP1CC	12	1 05E-08
NSF	17	1 12E-08
VWA7	6	1 12E-08
OR2J2	6	1 13E-08
IGSF9B	11	1 76E-08
ZCCHC7	9	1 79E-08
TRIM26	6	1 81E-08
EGFL8	6	2 22E-08
DARS2	1	2 47E-08
CENPM	22	3 15E-08
ATP2A2	12	3 68E-08
NEU1	6	4 16E-08
DNAJA3	16	4 53E-08
PPTC7	12	5 29E-08
INHBE	12	6 56E-08
TRIM31	6	7 59E-08
CLP 1	11	7 62E-08
GATAD2A	19	9 23E-08
ZSCAN23	6	1 04E-07
HLA-DQB1	6	1 06E-07
AGPAT1	6	1 21E-07
LCMT2	15	1 21E-07
SIRPB1	20	1 30E-07
ZSCAN16	6	1 43E-07
BTN3A2	6	1 60E-07
TRIM38	6	1 73E-07
VPS29	12	1 80E-07
VARS	6	2 06E-07
RP4-576H24.4	20	2 06E-07
PPP1R16B	20	2 23E-07
ZKSCAN8	6	2 26E-07
FXR1	3	2 59E-07
BAG6	6	2 67E-07
BTBD18	11	2 93E-07
GPN3	12	3 36E-07
ZBTB37	1	3 46E-07
WBP2NL	22	3 60E-07
KCNMA1	10	3 60E-07
VRK2	2	3 65E-07

TRANK1	3	9 03E-09
SP4	7	1 02E-08
KCNB1	20	1 04E-08
ITIH1	3	1 43E-08
ZSCAN2	15	1 78E-08
PACS1	11	1 80E-08
CACNB2	10	1 81E-08
CUL4A	13	2 50E-08
NT5C	17	3 10E-08
SSBP2	5	3 31E-08
PLEC	8	3 38E-08
SEC11A	15	3 56E-08
OSBPL2	20	4 12E-08
SUMO2	17	4 15E-08
PARP10	8	4 42E-08
GRIN2A	16	4 62E-08
WFDC5	20	4 96E-08
PGBD1	6	5 79E-08
ZKSCAN8	6	5 98E-08
DPCR1	6	6 18E-08
ZKSCAN4	6	6 27E-08
PALM2-AKAP2	9	6 28E-08
AKAP2	9	6 28E-08
STK4	20	6 43E-08
FEN1	11	6 54E-08
HIST1H2AJ	6	7 80E-08
ALAS1	3	8 75E-08
OR2B2	6	8 78E-08
SPCS1	3	9 17E-08
ZSCAN16	6	1 23E-07
CERS6	2	1 29E-07
C17orf53	17	1 30E-07
HIST1H3I	6	1 57E-07
ASB16	17	1 73E-07
LRRC57	15	1 78E-07
ALOX5AP	13	2 22E-07
GABBR1	6	2 23E-07
HIST1H2BL	6	2 31E-07
MAD1L1	7	2 51E-07
RBMS3	3	2 79E-07
MACROD1	11	3 20E-07
SYNE1	6	3 34E-07
TRPT1	11	3 46E-07

PLCH2	1	3 66E-07
HIST1H3I	6	3 98E-07
TBC1D15	12	3 99E-07
DSTYK	1	4 15E-07
BTN2A1	6	4 17E-07
STAU1	20	4 35E-07
ARNTL	11	4 56E-07
HIST1H3J	6	5 66E-07
KLHL20	1	6 59E-07
PLCL2	3	7 40E-07
MED19	11	7 48E-07
GALNT10	5	8 62E-07
PLEKHM1	17	9 66E-07
NAGA	22	1 03E-06
KCNJ13	2	1 09E-06
CORO7	16	1 09E-06

PPP1R18	6	3 47E-07
GRINA	8	3 57E-07
HTR6	1	3 91E-07
STARD9	15	4 06E-07
FKBP2	11	4 21E-07
ZSCAN31	6	4 30E-07
MSH5-SAPCD1	6	4 49E-07
STARD7	2	4 55E-07
MSH5	6	4 71E-07
XPNPEP1	10	5 11E-07
PACSIN2	22	5 19E-07
CNTN5	11	5 29E-07
MRPS33	7	5 59E-07
TMUB2	17	5 79E-07
FURIN	15	5 91E-07
UBE2E3	2	6 19E-07
