# Supplementary material for: Splitting schizophrenia: divergent cognitive and educational outcomes revealed by genomic structural equation modelling
Source: Mol Psychiatry. 2026 Jan 31;31(6):3098–107. doi: 10.1038/s41380-026-03444-3 (PMC13190233; doi:10.1038/s41380-026-03444-3)
Supplement: Supplementary file 11 — Supplemental table 9 [file 41380_2026_3444_MOESM11_ESM.pdf]

MR estimates when the exposures are genetic components of psychotic disorders and the outcomes are years of education (Lee et al.2018) and IQ (Savage et al., 2018)

| Exposure | Outcome | method | nsnp | b | lower_b | upper_b | se | OR | lower_OR | upper_OR | pval |
|---|---|---|---|---|---|---|---|---|---|---|---|
| Schizophrenia | EA | Inverse variance weighted | 146 | -0 00094 | -0 01537 | 0 01349 | 0 00736 | 0 99906 | 0 98475 | 1 01358 | 0 89863 |
| | EA | Weighted median | 146 | 0 00414 | -0 00512 | 0 01340 | 0 00472 | 1 00415 | 0 99489 | 1 01349 | 0 38111 |
| | EA | Penalised weighted median | 146 | 0 00283 | -0 00672 | 0 01239 | 0 00487 | 1 00284 | 0 99330 | 1 01246 | 0 56116 |
| | EA | MR Egger | 146 | 0 03408 | -0 02291 | 0 09107 | 0 02908 | 1 03467 | 0 97735 | 1 09534 | 0 24309 |
| | EA | Constrained Maximum Likelihood | 120 | 0 01254 | 0 00685 | 0 01823 | 0 00290 | 1 01262 | 1 00687 | 1 01839 | 0 00002 |
| | IQ | Inverse variance weighted | 145 | -0 04382 | -0 06398 | -0 02366 | 0 01028 | 0 95713 | 0 93803 | 0 97662 | 0 00002 |
| | IQ | Weighted median | 145 | -0 02662 | -0 04163 | -0 01160 | 0 00766 | 0 97373 | 0 95922 | 0 98847 | 0 00051 |
| | IQ | Penalised weighted median | 145 | -0 02310 | -0 03903 | -0 00717 | 0 00813 | 0 97716 | 0 96172 | 0 99285 | 0 00448 |
| | IQ | MR Egger | 145 | -0 06467 | -0 14718 | 0 01784 | 0 04210 | 0 93738 | 0 86314 | 1 01800 | 0 12671 |
| | IQ | Constrained Maximum Likelihood | 117 | -0 03045 | -0 04371 | -0 01720 | 0 00676 | 0 97000 | 0 95723 | 0 98295 | 0 00001 |
| Bipolar disorder | EA | Inverse variance weighted | 44 | 0 04889 | 0 02385 | 0 07393 | 0 01278 | 1 05010 | 1 02414 | 1 07673 | 0 00013 |
| | EA | Weighted median | 44 | 0 02400 | 0 00611 | 0 04189 | 0 00913 | 1 02429 | 1 00613 | 1 04278 | 0 00855 |
| | EA | Penalised weighted median | 44 | 0 02036 | 0 00220 | 0 03853 | 0 00927 | 1 02057 | 1 00220 | 1 03928 | 0 02803 |
| | EA | MR Egger | 44 | 0 05553 | -0 07006 | 0 18113 | 0 06408 | 1 05710 | 0 93233 | 1 19857 | 0 39108 |
| | EA | Constrained Maximum Likelihood | 44 | 0 06240 | 0 05195 | 0 07285 | 0 00533 | 1 06438 | 1 05332 | 1 07557 | 1 23E-31 |
| | IQ | Inverse variance weighted | 45 | 0 01695 | -0 02204 | 0 05593 | 0 01989 | 1 01709 | 0 97820 | 1 05753 | 0 39430 |
| | IQ | Weighted median | 45 | 0 02207 | -0 00300 | 0 04713 | 0 01279 | 1 02231 | 0 99701 | 1 04826 | 0 08440 |
| | IQ | Penalised weighted median | 45 | 0 02370 | -0 00212 | 0 04952 | 0 01317 | 1 02398 | 0 99788 | 1 05077 | 0 07206 |
| | IQ | MR Egger | 45 | -0 01864 | -0 22181 | 0 18452 | 0 10365 | 0 98153 | 0 80107 | 1 20264 | 0 85810 |
| | IQ | Constrained Maximum Likelihood | 45 | 0 01259 | -0 00366 | 0 02884 | 0 00829 | 1 01267 | 0 99634 | 1 02926 | 0 12893 |
| SZspecific | EA | Inverse variance weighted | 29 | -0 01566 | -0 03300 | 0 00169 | 0 00885 | 0 98447 | 0 96754 | 1 00169 | 0 07688 |
| | EA | Weighted median | 29 | -0 01430 | -0 02587 | -0 00273 | 0 00590 | 0 98580 | 0 97446 | 0 99727 | 0 01543 |
| | EA | Penalised weighted median | 29 | -0 01357 | -0 02504 | -0 00210 | 0 00585 | 0 98652 | 0 97527 | 0 99790 | 0 02043 |
| | EA | MR Egger | 29 | -0 04310 | -0 12299 | 0 03679 | 0 04076 | 0 95781 | 0 88427 | 1 03748 | 0 29969 |
| | EA | Constrained Maximum Likelihood | 24 | -0 01337 | -0 02103 | -0 00571 | 0 00391 | 0 98672 | 0 97919 | 0 99431 | 0 00063 |
| | IQ | Inverse variance weighted | 30 | -0 04179 | -0 06732 | -0 01625 | 0 01303 | 0 95907 | 0 93489 | 0 98388 | 0 00134 |
| | IQ | Weighted median | 30 | -0 04253 | -0 06039 | -0 02467 | 0 00911 | 0 95837 | 0 94140 | 0 97564 | 3 06E-06 |
| | IQ | Penalised weighted median | 30 | -0 05336 | -0 07101 | -0 03571 | 0 00900 | 0 94804 | 0 93146 | 0 96492 | 3 09E-09 |
| | IQ | MR Egger | 30 | -0 12183 | -0 22707 | -0 01658 | 0 05370 | 0 88530 | 0 79686 | 0 98356 | 0 03119 |
| | IQ | Constrained Maximum Likelihood | 23 | -0 05972 | -0 07228 | -0 04716 | 0 00641 | 0 94203 | 0 93027 | 0 95393 | 1 16E-20 |
| PSYshared | EA | Inverse variance weighted | 37 | 0 03698 | 0 01612 | 0 05784 | 0 01064 | 1 03767 | 1 01625 | 1 05955 | 0 00051 |
| | EA | Weighted median | 37 | 0 01755 | 0 00385 | 0 03125 | 0 00699 | 1 01770 | 1 00385 | 1 03174 | 0 01207 |
| | EA | Penalised weighted median | 37 | 0 01494 | 0 00114 | 0 02875 | 0 00704 | 1 01506 | 1 00114 | 1 02917 | 0 03386 |
| | EA | MR Egger | 37 | 0 01861 | -0 08496 | 0 12218 | 0 05284 | 1 01879 | 0 91855 | 1 12996 | 0 72681 |
| | EA | Constrained Maximum Likelihood | 36 | 0 04278 | 0 03305 | 0 05252 | 0 00497 | 1 04371 | 1 03360 | 1 05392 | 7 11E-18 |
| | IQ | Inverse variance weighted | 38 | 0 00642 | -0 02715 | 0 03998 | 0 01713 | 1 00644 | 0 97321 | 1 04079 | 0 70797 |
| | IQ | Weighted median | 38 | -0 00147 | -0 02293 | 0 01999 | 0 01095 | 0 99853 | 0 97733 | 1 02019 | 0 89309 |
| | IQ | Penalised weighted median | 38 | -0 00792 | -0 02982 | 0 01397 | 0 01117 | 0 99211 | 0 97062 | 1 01407 | 0 47810 |
| | IQ | MR Egger | 38 | -0 03482 | -0 20774 | 0 13810 | 0 08823 | 0 96578 | 0 81242 | 1 14809 | 0 69542 |
| | IQ | Constrained Maximum Likelihood | 36 | 0 00778 | -0 00684 | 0 02240 | 0 00746 | 1 00781 | 0 99319 | 1 02265 | 0 29677 |

**Heterogeneity tests**

| Exposure | Outcome | Method | Q | Q_df | Q_pval |
|---|---|---|---|---|---|
| **Schizophrenia** | **EA** | MR Egger | 131997 | 144 | 478E-189 |
| **Schizophrenia** | **EA** | Inverse variance weighted | 133417 | 145 | 257E-191 |
| **Schizophrenia** | **IQ** | MR Egger | 97272 | 143 | 187E-123 |
| **Schizophrenia** | **IQ** | Inverse variance weighted | 97449 | 144 | 229E-123 |
| **Bipolar** | **EA** | MR Egger | 37074 | 42 | 330E-54 |
| **Bipolar** | **EA** | Inverse variance weighted | 37084 | 43 | 946E-54 |
| **Bipolar** | **IQ** | MR Egger | 34715 | 43 | 344E-49 |
| **Bipolar** | **IQ** | Inverse variance weighted | 34814 | 44 | 639E-49 |
| **SZspecific** | **EA** | MR Egger | 23193 | 27 | 181E-34 |
| **SZspecific** | **EA** | Inverse variance weighted | 23602 | 28 | 869E-35 |
| **SZspecific** | **IQ** | MR Egger | 20212 | 28 | 272E-28 |
| **SZspecific** | **IQ** | Inverse variance weighted | 21911 | 29 | 445E-31 |
| **PSYshared** | **EA** | MR Egger | 31170 | 35 | 409E-46 |
| **PSYshared** | **EA** | Inverse variance weighted | 31282 | 36 | 747E-46 |
| **PSYshared** | **IQ** | MR Egger | 31730 | 36 | 101E-46 |
| **PSYshared** | **IQ** | Inverse variance weighted | 31930 | 37 | 124E-46 |

| | Min_F | Mean_F | Max_F | I_squared |
|---|---|---|---|---|
| **Schizophrenia_EA** | 29 46 | 44 47 | 175 28 | 0 98 |
| **Schizophrenia_IQ** | 29 46 | 44 82 | 175 28 | 0 98 |
| **Bipolar_EA** | 29 75 | 38 50 | 79 65 | 0 97 |
| **Bipolar_IQ** | 29 75 | 38 45 | 79 65 | 0 97 |
| **SZspecific_EA** | 29 86 | 36 54 | 59 26 | 0 97 |
| **SZspecific_IQ** | 30 00 | 38 19 | 67 73 | 0 97 |
| **PSYshared_EA** | 30 03 | 39 05 | 77 32 | 0 97 |
| **PSYshared_IQ** | 30 03 | 39 08 | 77 32 | 0 97 |

**Tests for pleiotropy**

| Exposure | Outcome | Egger Intercept | SE | pval |
|---|---|---|---|---|
| **Schizophrenia** | **EA** | -00024 | 00019 | 02152 |
| **Schizophrenia** | **IQ** | 00014 | 00028 | 06103 |
| **Bipolar** | **EA** | -00005 | 00043 | 09162 |
| **Bipolar** | **IQ** | 00024 | 00069 | 07281 |
| **SZspecifc** | **EA** | 00034 | 00049 | 04960 |
| **SZspecifc** | **IQ** | 00102 | 00066 | 01362 |
| **PSYshared** | **EA** | 00016 | 00046 | 07247 |
| **PSYshared** | **IQ** | 00037 | 0 0077 | 0 6365 |
