# Supplementary material for: Splitting schizophrenia: divergent cognitive and educational outcomes revealed by genomic structural equation modelling
Source: Mol Psychiatry. 2026 Jan 31;31(6):3098–107. doi: 10.1038/s41380-026-03444-3 (PMC13190233; doi:10.1038/s41380-026-03444-3)
Supplement: Supplementary file 12 — Supplemental table 10 [file 41380_2026_3444_MOESM12_ESM.pdf]

Exposure	Outcome	SNP	b	se	p
Schizophrenia	EA	rs1000237	-0.0022569	0.00734153	0.75852432
Schizophrenia	EA	rs10035564	-0.0015558	0.00739353	0.83333466
Schizophrenia	EA	rs10086619	-0.0008128	0.0074071	0.9126251
Schizophrenia	EA	rs10108980	-0.0008682	0.00740536	0.90667341
Schizophrenia	EA	rs10117	-0.0008217	0.00740739	0.91167297
Schizophrenia	EA	rs10861176	-0.0013859	0.00738533	0.85114972
Schizophrenia	EA	rs10876446	-0.0005119	0.00738828	0.94475719
Schizophrenia	EA	rs11027839	-0.0007624	0.00740401	0.91798591
Schizophrenia	EA	rs11136325	-0.00113	0.00740362	0.87869601
Schizophrenia	EA	rs11165867	-0.0013131	0.00739869	0.8591355
Schizophrenia	EA	rs11191580	-0.0011252	0.00742439	0.87953361
Schizophrenia	EA	rs11210892	0.00089306	0.00719093	0.90116293
Schizophrenia	EA	rs11223774	-0.0009855	0.00740324	0.89410088
Schizophrenia	EA	rs113264400	-0.0007735	0.00740121	0.91676117
Schizophrenia	EA	rs11534045	-0.0007726	0.00741024	0.9169573
Schizophrenia	EA	rs11587347	-0.0006484	0.00740906	0.93026599
Schizophrenia	EA	rs11664298	0.00034459	0.00731949	0.96245124
Schizophrenia	EA	rs11693094	-0.001274	0.00739985	0.8633035
Schizophrenia	EA	rs117178087	-0.0008994	0.00740333	0.90330628
Schizophrenia	EA	rs11941714	-0.0008537	0.00740368	0.90819926
Schizophrenia	EA	rs1198588	-0.0025115	0.00735771	0.73284336
Schizophrenia	EA	rs12129573	-0.0005493	0.00742119	0.94100084
Schizophrenia	EA	rs12138231	-0.0004978	0.00738991	0.94629713
Schizophrenia	EA	rs12151767	-0.0012172	0.00741009	0.86952425
Schizophrenia	EA	rs12285419	-0.0012167	0.00741647	0.86968479
Schizophrenia	EA	rs12293670	-0.0015346	0.00740174	0.8357511
Schizophrenia	EA	rs12303743	-0.0010073	0.00740696	0.89183037
Schizophrenia	EA	rs12489270	0.00033478	0.00729029	0.96337264
Schizophrenia	EA	rs12652777	-0.0009823	0.00740443	0.89446061
Schizophrenia	EA	rs12712510	-0.0008125	0.00741016	0.91268901
Schizophrenia	EA	rs12771371	-0.0008444	0.00740328	0.90919733
Schizophrenia	EA	rs12833624	-0.00113	0.00739982	0.87863343
Schizophrenia	EA	rs12877581	-0.001251	0.00739927	0.86573929
Schizophrenia	EA	rs12883788	-0.0002968	0.00738928	0.96796357
Schizophrenia	EA	rs13016542	-0.0015325	0.00739094	0.83573885
Schizophrenia	EA	rs13107325	0.00055401	0.00734997	0.93991606
Schizophrenia	EA	rs13195636	-0.0005339	0.00751236	0.9433414
Schizophrenia	EA	rs13233308	-0.0006132	0.00739433	0.933909
Schizophrenia	EA	rs132582	5.88E-05	0.00731662	0.99358572
Schizophrenia	EA	rs1427633	-0.0008243	0.00740255	0.91133993
Schizophrenia	EA	rs1430894	-0.001035	0.00740734	0.88888052
Schizophrenia	EA	rs145071536	-0.0014887	0.00739853	0.84052593
Schizophrenia	EA	rs1451488	-0.0012401	0.00742008	0.86726541
Schizophrenia	EA	rs149165	-0.0012035	0.00739689	0.8707488
Schizophrenia	EA	rs1593304	-0.0010586	0.00740435	0.88631137
Schizophrenia	EA	rs1604060	-0.0005371	0.00739086	0.94206597
Schizophrenia	EA	rs1615350	-0.0033397	0.00708102	0.63718205

Schizophrenia	EA	rs167924	-0.0010917	0.0074023	0.88275316
Schizophrenia	EA	rs16851048	-0.0012082	0.00740804	0.87044124
Schizophrenia	EA	rs16867571	-0.0019204	0.0073406	0.793621
Schizophrenia	EA	rs17016552	-0.0009223	0.00740482	0.90087448
Schizophrenia	EA	rs17194490	-0.0017585	0.00736541	0.81130339
Schizophrenia	EA	rs17731	-0.0009687	0.0074059	0.89593246
Schizophrenia	EA	rs187557	-0.0015277	0.00736751	0.83572995
Schizophrenia	EA	rs1881046	-0.0002709	0.00735918	0.97063893
Schizophrenia	EA	rs1901512	-0.0011353	0.00740529	0.87815028
Schizophrenia	EA	rs1915019	-0.0012409	0.00739571	0.8667461
Schizophrenia	EA	rs2053079	-0.0006212	0.00739819	0.93308482
Schizophrenia	EA	rs2078266	-0.0006374	0.00739623	0.93132175
Schizophrenia	EA	rs215412	-0.0012653	0.00740077	0.86424939
Schizophrenia	EA	rs217336	-0.0009354	0.00740553	0.89948359
Schizophrenia	EA	rs2238057	-0.0007226	0.00743626	0.92259335
Schizophrenia	EA	rs2252074	-0.0002754	0.00739805	0.97031009
Schizophrenia	EA	rs2332700	-0.0015823	0.00739625	0.83059967
Schizophrenia	EA	rs2333321	-0.0001668	0.00737052	0.98194609
Schizophrenia	EA	rs2381411	-0.0007096	0.00739974	0.9236055
Schizophrenia	EA	rs2455415	-0.0008899	0.00740449	0.90434124
Schizophrenia	EA	rs2456020	-0.0004462	0.00741061	0.95198405
Schizophrenia	EA	rs2514218	-0.0001566	0.00738927	0.98309427
Schizophrenia	EA	rs2710323	-0.0010144	0.00743312	0.89145401
Schizophrenia	EA	rs2815731	-0.001203	0.00740688	0.87097268
Schizophrenia	EA	rs2909457	0.00017428	0.00728365	0.98091069
Schizophrenia	EA	rs2999392	-0.0007039	0.00739819	0.92419859
Schizophrenia	EA	rs308697	-0.0014993	0.00737607	0.83892725
Schizophrenia	EA	rs35351411	-0.000908	0.00741676	0.902559
Schizophrenia	EA	rs35734242	-0.0013027	0.00739196	0.86011099
Schizophrenia	EA	rs3739118	-0.0007137	0.00740236	0.92319565
Schizophrenia	EA	rs3770754	-0.0007348	0.00740246	0.92092922
Schizophrenia	EA	rs3791710	-0.0013689	0.00738596	0.85296474
Schizophrenia	EA	rs3795310	-0.0006236	0.00739722	0.93281132
Schizophrenia	EA	rs3802924	-0.0001208	0.00736949	0.98692286
Schizophrenia	EA	rs3814883	-0.0010077	0.0074201	0.89197497
Schizophrenia	EA	rs3824451	-0.0007493	0.00740019	0.91934635
Schizophrenia	EA	rs4129585	0.00079309	0.00730417	0.91353491
Schizophrenia	EA	rs4575535	-0.0011619	0.0074021	0.87526782
Schizophrenia	EA	rs4632195	5.89E-05	0.0073001	0.99356443
Schizophrenia	EA	rs4636654	-0.0003238	0.0073644	0.96493064
Schizophrenia	EA	rs4653164	-0.0009637	0.00740358	0.89643048
Schizophrenia	EA	rs4702	-0.0009662	0.0074388	0.89665779
Schizophrenia	EA	rs4766428	-0.0006979	0.00742646	0.92512696
Schizophrenia	EA	rs4779050	-0.0005904	0.00740115	0.93641421
Schizophrenia	EA	rs4812325	-0.0016862	0.00739796	0.81970442
Schizophrenia	EA	rs4921741	-0.0012938	0.00739146	0.86105107
Schizophrenia	EA	rs498591	-0.0012007	0.00740009	0.8711057
Schizophrenia	EA	rs500102	-0.0011431	0.00740237	0.87727103

Schizophrenia	EA	rs505061	-0.0009805	0.00740812	0.89470093
Schizophrenia	EA	rs56205728	-0.0012428	0.00740393	0.86669348
Schizophrenia	EA	rs56335113	-0.001272	0.00740611	0.86363511
Schizophrenia	EA	rs57433322	0.00014521	0.00730632	0.98414369
Schizophrenia	EA	rs5751191	-0.0001086	0.00738245	0.98825915
Schizophrenia	EA	rs58120505	-0.001968	0.00741534	0.79070011
Schizophrenia	EA	rs60135207	0.00033162	0.00724886	0.96351166
Schizophrenia	EA	rs6125656	-0.0009604	0.00740518	0.89680421
Schizophrenia	EA	rs61857878	-0.0002383	0.00736309	0.97418631
Schizophrenia	EA	rs61937595	-0.000548	0.00742115	0.94113349
Schizophrenia	EA	rs62018952	-0.0005273	0.00739233	0.9431341
Schizophrenia	EA	rs62183855	-0.0007861	0.00740452	0.9154476
Schizophrenia	EA	rs634940	-0.0012843	0.00740408	0.86229605
Schizophrenia	EA	rs6482437	-0.0016544	0.00738594	0.82276121
Schizophrenia	EA	rs6520064	-0.0011262	0.00739915	0.87901883
Schizophrenia	EA	rs6538539	-0.0006077	0.00740288	0.9345769
Schizophrenia	EA	rs6546857	-0.0001942	0.00735882	0.97894474
Schizophrenia	EA	rs6549963	-0.0008803	0.00740351	0.90535291
Schizophrenia	EA	rs6673880	-0.001161	0.00741158	0.87552295
Schizophrenia	EA	rs6715366	-0.000999	0.00740396	0.89266969
Schizophrenia	EA	rs6798742	-0.0009239	0.0074102	0.90078221
Schizophrenia	EA	rs6943762	-0.0012375	0.00742032	0.8675494
Schizophrenia	EA	rs6974218	-0.0015759	0.00737574	0.83081198
Schizophrenia	EA	rs6984242	-0.0008805	0.00740821	0.90538998
Schizophrenia	EA	rs708228	-2.01E-05	0.00732599	0.99781628
Schizophrenia	EA	rs7112616	-0.0015837	0.00737192	0.82989727
Schizophrenia	EA	rs713692	-0.0014444	0.00738522	0.8449427
Schizophrenia	EA	rs7251	-0.0010186	0.00741274	0.89070362
Schizophrenia	EA	rs72802868	-0.0020998	0.00733896	0.77478702
Schizophrenia	EA	rs728055	0.0006483	0.00727525	0.92899478
Schizophrenia	EA	rs72943392	-0.0010631	0.00740276	0.88580467
Schizophrenia	EA	rs72986630	-0.0015834	0.00737216	0.8299342
Schizophrenia	EA	rs73229090	-0.0008115	0.00741887	0.91290356
Schizophrenia	EA	rs73292401	-0.0015508	0.00737743	0.8335002
Schizophrenia	EA	rs7515363	-0.0009744	0.00740717	0.89534267
Schizophrenia	EA	rs7575796	-0.0013316	0.00739051	0.85701704
Schizophrenia	EA	rs7634476	-0.0007636	0.00740909	0.9179116
Schizophrenia	EA	rs7647398	-0.0009778	0.00741496	0.89508743
Schizophrenia	EA	rs76838079	-0.0010319	0.00740808	0.88921647
Schizophrenia	EA	rs778371	-0.0022884	0.00734644	0.75541883
Schizophrenia	EA	rs7798283	-0.0005535	0.00739029	0.94029913
Schizophrenia	EA	rs7830315	-0.0013747	0.00738464	0.85232065
Schizophrenia	EA	rs79210963	-0.0026246	0.00719289	0.71520017
Schizophrenia	EA	rs79445414	-0.0008208	0.00740617	0.91175898
Schizophrenia	EA	rs8055219	-0.0017461	0.00736148	0.81250144
Schizophrenia	EA	rs9304548	-0.0009887	0.00740466	0.89378046
Schizophrenia	EA	rs9318627	-0.0009303	0.00741337	0.90013368
Schizophrenia	EA	rs9461916	-0.0012119	0.00740105	0.86992716

Schizophrenia	EA	rs9636107	-0.000412	0.00741068	0.95566381
Schizophrenia	EA	rs9687282	-0.0009082	0.00740499	0.90238417
Schizophrenia	EA	rs9876421	-0.0015451	0.0073892	0.8343723
Schizophrenia	EA	All	-0.0009377	0.00736112	0.8986305
Schizophrenia	IQ	rs1000237	-0.0448775	0.01033796	1.42E-05
Schizophrenia	IQ	rs10035564	-0.0442749	0.01035653	1.91E-05
Schizophrenia	IQ	rs10086619	-0.0434214	0.01034084	2.68E-05
Schizophrenia	IQ	rs10108980	-0.0431505	0.01032128	2.91E-05
Schizophrenia	IQ	rs10117	-0.0433617	0.0103412	2.75E-05
Schizophrenia	IQ	rs10861176	-0.0444063	0.01032188	1.69E-05
Schizophrenia	IQ	rs10873538	-0.0455632	0.01023243	8.48E-06
Schizophrenia	IQ	rs10876446	-0.0451835	0.01022203	9.86E-06
Schizophrenia	IQ	rs11027839	-0.0440969	0.01034421	2.02E-05
Schizophrenia	IQ	rs11136325	-0.0435038	0.01034366	2.60E-05
Schizophrenia	IQ	rs11165867	-0.0443255	0.01034089	1.82E-05
Schizophrenia	IQ	rs11191580	-0.0439862	0.01038224	2.27E-05
Schizophrenia	IQ	rs11210892	-0.0425209	0.01028717	3.57E-05
Schizophrenia	IQ	rs11223774	-0.0443487	0.01032279	1.74E-05
Schizophrenia	IQ	rs113264400	-0.0441896	0.01033954	1.92E-05
Schizophrenia	IQ	rs11534045	-0.0434875	0.01035394	2.67E-05
Schizophrenia	IQ	rs11587347	-0.0451711	0.01027993	1.11E-05
Schizophrenia	IQ	rs11664298	-0.0427593	0.01030959	3.36E-05
Schizophrenia	IQ	rs11693094	-0.0438674	0.0103515	2.26E-05
Schizophrenia	IQ	rs117178087	-0.0433579	0.01032863	2.69E-05
Schizophrenia	IQ	rs11941714	-0.0438394	0.01032717	2.19E-05
Schizophrenia	IQ	rs1198588	-0.0453451	0.01034234	1.16E-05
Schizophrenia	IQ	rs12129573	-0.0438729	0.01038151	2.38E-05
Schizophrenia	IQ	rs12138231	-0.0433995	0.01033715	2.69E-05
Schizophrenia	IQ	rs12151767	-0.0449362	0.01030736	1.30E-05
Schizophrenia	IQ	rs12285419	-0.0432738	0.01035907	2.95E-05
Schizophrenia	IQ	rs12293670	-0.0449926	0.01031589	1.29E-05
Schizophrenia	IQ	rs12303743	-0.0442827	0.01033279	1.82E-05
Schizophrenia	IQ	rs12489270	-0.0427655	0.01029589	3.27E-05
Schizophrenia	IQ	rs12652777	-0.0432573	0.01032431	2.79E-05
Schizophrenia	IQ	rs12712510	-0.0441332	0.01035041	2.01E-05
Schizophrenia	IQ	rs12771371	-0.0442206	0.01033419	1.88E-05
Schizophrenia	IQ	rs12833624	-0.0448761	0.01026462	1.23E-05
Schizophrenia	IQ	rs12877581	-0.0442446	0.01033704	1.87E-05
Schizophrenia	IQ	rs12883788	-0.041649	0.01014788	4.06E-05
Schizophrenia	IQ	rs13016542	-0.0441723	0.01035422	1.99E-05
Schizophrenia	IQ	rs13107325	-0.0400831	0.01001402	6.26E-05
Schizophrenia	IQ	rs13195636	-0.042018	0.01041804	5.50E-05
Schizophrenia	IQ	rs13233308	-0.0433978	0.01033407	2.68E-05
Schizophrenia	IQ	rs132582	-0.0425613	0.0102446	3.26E-05
Schizophrenia	IQ	rs1430894	-0.0441297	0.01034539	1.99E-05
Schizophrenia	IQ	rs145071536	-0.0440887	0.01035678	2.07E-05
Schizophrenia	IQ	rs1451488	-0.0459718	0.01022169	6.88E-06
Schizophrenia	IQ	rs149165	-0.0438242	0.01034481	2.27E-05

Schizophrenia	IQ	rs1593304	-0.043552	0.01034578	2.56E-05
Schizophrenia	IQ	rs1604060	-0.043413	0.01033414	2.66E-05
Schizophrenia	IQ	rs1615350	-0.0463258	0.01012896	4.79E-06
Schizophrenia	IQ	rs167924	-0.0444648	0.01031753	1.64E-05
Schizophrenia	IQ	rs16851048	-0.044102	0.01035556	2.06E-05
Schizophrenia	IQ	rs16867571	-0.044664	0.01031628	1.49E-05
Schizophrenia	IQ	rs17016552	-0.0443615	0.0103271	1.74E-05
Schizophrenia	IQ	rs17194490	-0.0447621	0.01031402	1.43E-05
Schizophrenia	IQ	rs17731	-0.0445708	0.01031067	1.54E-05
Schizophrenia	IQ	rs187557	-0.0445712	0.01030905	1.54E-05
Schizophrenia	IQ	rs1881046	-0.0431979	0.0103164	2.82E-05
Schizophrenia	IQ	rs1901512	-0.0443359	0.01033657	1.79E-05
Schizophrenia	IQ	rs1915019	-0.0437135	0.01034659	2.39E-05
Schizophrenia	IQ	rs2053079	-0.0435142	0.01034177	2.58E-05
Schizophrenia	IQ	rs215412	-0.0433381	0.01033757	2.76E-05
Schizophrenia	IQ	rs217336	-0.0438296	0.01034685	2.28E-05
Schizophrenia	IQ	rs2238057	-0.0429542	0.01037559	3.47E-05
Schizophrenia	IQ	rs2252074	-0.0429475	0.01034254	3.29E-05
Schizophrenia	IQ	rs2332700	-0.0439889	0.01036428	2.19E-05
Schizophrenia	IQ	rs2333321	-0.0430559	0.01032694	3.06E-05
Schizophrenia	IQ	rs2381411	-0.0436162	0.01034361	2.48E-05
Schizophrenia	IQ	rs2455415	-0.0439908	0.01034379	2.11E-05
Schizophrenia	IQ	rs2456020	-0.0447509	0.01034698	1.53E-05
Schizophrenia	IQ	rs2514218	-0.0443649	0.01035741	1.84E-05
Schizophrenia	IQ	rs2710323	-0.0418099	0.01028133	4.77E-05
Schizophrenia	IQ	rs2815731	-0.0442801	0.01034382	1.86E-05
Schizophrenia	IQ	rs2909457	-0.0420477	0.01012525	3.28E-05
Schizophrenia	IQ	rs2999392	-0.0438969	0.01034504	2.20E-05
Schizophrenia	IQ	rs308697	-0.044422	0.01032367	1.69E-05
Schizophrenia	IQ	rs35351411	-0.0431257	0.01034393	3.06E-05
Schizophrenia	IQ	rs35734242	-0.0450555	0.01024648	1.10E-05
Schizophrenia	IQ	rs3739118	-0.043927	0.01034776	2.19E-05
Schizophrenia	IQ	rs3770754	-0.044214	0.01033838	1.90E-05
Schizophrenia	IQ	rs3791710	-0.0440726	0.01034096	2.03E-05
Schizophrenia	IQ	rs3795310	-0.0430941	0.01031604	2.95E-05
Schizophrenia	IQ	rs3802924	-0.0422691	0.0102386	3.65E-05
Schizophrenia	IQ	rs3814883	-0.0438508	0.01036805	2.34E-05
Schizophrenia	IQ	rs3824451	-0.0438697	0.01034585	2.23E-05
Schizophrenia	IQ	rs4129585	-0.0417	0.0102488	4.73E-05
Schizophrenia	IQ	rs4575535	-0.0437688	0.01034798	2.34E-05
Schizophrenia	IQ	rs4632195	-0.0425181	0.01022195	3.19E-05
Schizophrenia	IQ	rs4636654	-0.0429009	0.01028297	3.02E-05
Schizophrenia	IQ	rs4653164	-0.0448411	0.0102753	1.28E-05
Schizophrenia	IQ	rs4702	-0.0434505	0.01039231	2.90E-05
Schizophrenia	IQ	rs4766428	-0.0434659	0.01037506	2.80E-05
Schizophrenia	IQ	rs4779050	-0.0436169	0.01035277	2.52E-05
Schizophrenia	IQ	rs4812325	-0.0442234	0.0103678	1.99E-05
Schizophrenia	IQ	rs4921741	-0.0439484	0.01034712	2.16E-05

Schizophrenia	IQ	rs498591	-0.0443677	0.01033197	1.75E-05
Schizophrenia	IQ	rs500102	-0.04376	0.01034751	2.35E-05
Schizophrenia	IQ	rs505061	-0.0444828	0.01032684	1.65E-05
Schizophrenia	IQ	rs56205728	-0.044067	0.01031634	1.94E-05
Schizophrenia	IQ	rs56335113	-0.0445756	0.01033339	1.61E-05
Schizophrenia	IQ	rs57433322	-0.0438355	0.01035614	2.31E-05
Schizophrenia	IQ	rs5751191	-0.0419267	0.01022867	4.15E-05
Schizophrenia	IQ	rs58120505	-0.0439616	0.01040369	2.38E-05
Schizophrenia	IQ	rs60135207	-0.0423206	0.01018903	3.27E-05
Schizophrenia	IQ	rs6125656	-0.0431366	0.01031642	2.90E-05
Schizophrenia	IQ	rs61857878	-0.0444002	0.01032546	1.71E-05
Schizophrenia	IQ	rs61937595	-0.0433809	0.01036493	2.85E-05
Schizophrenia	IQ	rs62018952	-0.0428971	0.01030103	3.12E-05
Schizophrenia	IQ	rs62183855	-0.0441468	0.01034235	1.97E-05
Schizophrenia	IQ	rs634940	-0.043511	0.01035131	2.63E-05
Schizophrenia	IQ	rs6482437	-0.0438587	0.01035898	2.30E-05
Schizophrenia	IQ	rs6520064	-0.0441422	0.01033838	1.96E-05
Schizophrenia	IQ	rs6538539	-0.0442287	0.01034695	1.92E-05
Schizophrenia	IQ	rs6546857	-0.0425513	0.01024838	3.30E-05
Schizophrenia	IQ	rs6549963	-0.0443334	0.01032424	1.75E-05
Schizophrenia	IQ	rs6673880	-0.0435824	0.01035819	2.58E-05
Schizophrenia	IQ	rs6715366	-0.0431593	0.0103135	2.86E-05
Schizophrenia	IQ	rs6798742	-0.0433079	0.01034313	2.83E-05
Schizophrenia	IQ	rs6943762	-0.0441488	0.01036676	2.06E-05
Schizophrenia	IQ	rs6974218	-0.0436407	0.01034908	2.48E-05
Schizophrenia	IQ	rs6984242	-0.0440345	0.01034913	2.09E-05
Schizophrenia	IQ	rs708228	-0.0428978	0.01028774	3.05E-05
Schizophrenia	IQ	rs7112616	-0.0439368	0.01034895	2.18E-05
Schizophrenia	IQ	rs713692	-0.0439956	0.0103478	2.12E-05
Schizophrenia	IQ	rs7251	-0.0454896	0.0102365	8.84E-06
Schizophrenia	IQ	rs72802868	-0.0453913	0.01025575	9.60E-06
Schizophrenia	IQ	rs728055	-0.0438947	0.01036374	2.28E-05
Schizophrenia	IQ	rs72943392	-0.0444122	0.01031854	1.68E-05
Schizophrenia	IQ	rs72986630	-0.0440971	0.01034449	2.02E-05
Schizophrenia	IQ	rs73229090	-0.044314	0.01035547	1.88E-05
Schizophrenia	IQ	rs73292401	-0.0450092	0.01027276	1.18E-05
Schizophrenia	IQ	rs7515363	-0.0437228	0.01034856	2.39E-05
Schizophrenia	IQ	rs7575796	-0.0448352	0.01027924	1.29E-05
Schizophrenia	IQ	rs7634476	-0.0424885	0.01026695	3.50E-05
Schizophrenia	IQ	rs7647398	-0.0431639	0.01034305	3.00E-05
Schizophrenia	IQ	rs76838079	-0.0438027	0.01034803	2.31E-05
Schizophrenia	IQ	rs778371	-0.0450489	0.01033219	1.30E-05
Schizophrenia	IQ	rs7798283	-0.042922	0.01029058	3.03E-05
Schizophrenia	IQ	rs7830315	-0.0441345	0.01033786	1.96E-05
Schizophrenia	IQ	rs79210963	-0.04477	0.01029217	1.36E-05
Schizophrenia	IQ	rs79445414	-0.0426786	0.0102511	3.14E-05
Schizophrenia	IQ	rs8055219	-0.0440712	0.01035054	2.06E-05
Schizophrenia	IQ	rs9304548	-0.0440729	0.01034244	2.03E-05
Schizophrenia	IQ	rs9318627	-0.0428202	0.01031351	3.30E-05

Schizophrenia	IQ	rs9461916	-0.0442573	0.01033748	1.86E-05
Schizophrenia	IQ	rs9636107	-0.0433837	0.01036701	2.85E-05
Schizophrenia	IQ	rs9687282	-0.0441081	0.01034282	2.00E-05
Schizophrenia	IQ	rs9876421	-0.0441371	0.01035415	2.02E-05
Schizophrenia	IQ	All	-0.043818	0.01028459	2.04E-05
Bipolar	EA	rs10043984	0.04953558	0.01301918	0.00014191
Bipolar	EA	rs10255167	0.04951997	0.01303685	0.0001456
Bipolar	EA	rs10737496	0.04753108	0.0129621	0.00024548
Bipolar	EA	rs10866641	0.04792993	0.01306032	0.00024266
Bipolar	EA	rs10994415	0.05027677	0.01303586	0.00011488
Bipolar	EA	rs112481526	0.04723049	0.01293981	0.00026223
Bipolar	EA	rs113779084	0.04717919	0.01305053	0.00030021
Bipolar	EA	rs11764361	0.0484539	0.01305219	0.00020537
Bipolar	EA	rs12575685	0.04961698	0.01306241	0.0001456
Bipolar	EA	rs12668848	0.04785357	0.0130156	0.00023634
Bipolar	EA	rs12932628	0.05131717	0.01275051	5.70E-05
Bipolar	EA	rs13044225	0.04883972	0.01304736	0.00018164
Bipolar	EA	rs13195402	0.05247621	0.01291387	4.83E-05
Bipolar	EA	rs1487445	0.0393579	0.01064537	0.000218
Bipolar	EA	rs17183814	0.04888818	0.01305069	0.00017966
Bipolar	EA	rs174592	0.05164275	0.01291733	6.39E-05
Bipolar	EA	rs2126180	0.04751466	0.01298165	0.00025208
Bipolar	EA	rs2273738	0.04664681	0.01292461	0.00030721
Bipolar	EA	rs228768	0.04755187	0.01300586	0.00025599
Bipolar	EA	rs2336147	0.05083653	0.01300969	9.32E-05
Bipolar	EA	rs237460	0.04886599	0.0130546	0.00018169
Bipolar	EA	rs2693698	0.05005366	0.01297727	0.00011477
Bipolar	EA	rs28455634	0.05016446	0.01302086	0.00011686
Bipolar	EA	rs28565152	0.04765565	0.01300724	0.00024852
Bipolar	EA	rs2953928	0.04948184	0.0130425	0.0001483
Bipolar	EA	rs35306827	0.05176318	0.0126793	4.46E-05
Bipolar	EA	rs35958438	0.05021063	0.01296402	0.00010747
Bipolar	EA	rs4447398	0.04924454	0.01305274	0.00016147
Bipolar	EA	rs4619651	0.04932134	0.01308585	0.00016387
Bipolar	EA	rs4790841	0.04742535	0.01293578	0.00024617
Bipolar	EA	rs5758064	0.04780042	0.01298373	0.00023181
Bipolar	EA	rs61554907	0.05106907	0.01281808	6.77E-05
Bipolar	EA	rs62581014	0.04898555	0.0130606	0.00017639
Bipolar	EA	rs67712855	0.04582145	0.01271723	0.00031445
Bipolar	EA	rs6887473	0.04938	0.01304277	0.00015309
Bipolar	EA	rs6946056	0.04812987	0.01301405	0.00021705
Bipolar	EA	rs6954854	0.05036519	0.01297763	0.00010406
Bipolar	EA	rs696366	0.05198882	0.01252745	3.32E-05
Bipolar	EA	rs6992333	0.05097121	0.01290372	7.81E-05
Bipolar	EA	rs7108878	0.04680007	0.01280942	0.00025862
Bipolar	EA	rs7201930	0.04941173	0.01304079	0.00015125
Bipolar	EA	rs748455	0.05060896	0.01297921	9.65E-05
Bipolar	EA	rs7707252	0.04889775	0.01303623	0.00017619
Bipolar	EA	rs9834970	0.04641853	0.0131057	0.00039732
Bipolar	EA	All	0.04888991	0.01277512	0.00012973

Bipolar	IQ	rs10043984	0.01704577	0.02031312	0.40138482
Bipolar	IQ	rs10255167	0.01629495	0.02031501	0.42248729
Bipolar	IQ	rs10737496	0.01423098	0.02010308	0.47900686
Bipolar	IQ	rs10866641	0.01635439	0.02037501	0.42216599
Bipolar	IQ	rs10994415	0.01651819	0.02042357	0.41864129
Bipolar	IQ	rs112481526	0.01611693	0.0203133	0.42753439
Bipolar	IQ	rs113779084	0.01226637	0.02005086	0.54069467
Bipolar	IQ	rs11764361	0.01846448	0.02027969	0.36256357
Bipolar	IQ	rs12575685	0.01928348	0.02022391	0.34033731
Bipolar	IQ	rs12668848	0.01834668	0.02028062	0.36565567
Bipolar	IQ	rs12932628	0.01733376	0.0201611	0.38991982
Bipolar	IQ	rs13044225	0.01806244	0.02028767	0.37329616
Bipolar	IQ	rs13195402	0.02368027	0.01963306	0.22776264
Bipolar	IQ	rs1487445	0.00185733	0.01658711	0.91084381
Bipolar	IQ	rs17183814	0.01925399	0.02010576	0.3382465
Bipolar	IQ	rs174592	0.01345825	0.02023467	0.50598119
Bipolar	IQ	rs2126180	0.01630186	0.02032748	0.42257509
Bipolar	IQ	rs2273738	0.0142531	0.02028502	0.48227922
Bipolar	IQ	rs228768	0.01683756	0.02037126	0.40850047
Bipolar	IQ	rs2336147	0.02452514	0.01933266	0.20458885
Bipolar	IQ	rs237460	0.01728506	0.02032715	0.39513402
Bipolar	IQ	rs2693698	0.01781534	0.02028531	0.37981414
Bipolar	IQ	rs28455634	0.01582845	0.02034573	0.43658446
Bipolar	IQ	rs28565152	0.02184461	0.01964928	0.26625618
Bipolar	IQ	rs2953928	0.02079151	0.01983749	0.29459642
Bipolar	IQ	rs35306827	0.01606583	0.02030777	0.42887555
Bipolar	IQ	rs35958438	0.01655478	0.02030444	0.41488464
Bipolar	IQ	rs41315395	0.01764552	0.0202806	0.38426284
Bipolar	IQ	rs4447398	0.01873938	0.0202588	0.35496601
Bipolar	IQ	rs4619651	0.0185217	0.02032119	0.36205959
Bipolar	IQ	rs4790841	0.01635923	0.02029283	0.42015158
Bipolar	IQ	rs5758064	0.01729501	0.020304	0.39432335
Bipolar	IQ	rs61554907	0.01740719	0.02030869	0.39137305
Bipolar	IQ	rs62581014	0.01921199	0.02026449	0.34309801
Bipolar	IQ	rs67712855	0.0133343	0.02009286	0.50692486
Bipolar	IQ	rs6887473	0.01572105	0.02027092	0.43801653
Bipolar	IQ	rs6946056	0.01498748	0.02017921	0.45765174
Bipolar	IQ	rs6954854	0.02187552	0.01970753	0.26699551
Bipolar	IQ	rs696366	0.01844809	0.01988369	0.35351119
Bipolar	IQ	rs6992333	0.0150533	0.0202744	0.4577978
Bipolar	IQ	rs7108878	0.01341876	0.01984915	0.49901739
Bipolar	IQ	rs7201930	0.01772754	0.02030907	0.3827241
Bipolar	IQ	rs748455	0.01866947	0.02031782	0.35816264
Bipolar	IQ	rs7707252	0.01685089	0.02029507	0.40637232
Bipolar	IQ	rs9834970	0.01430895	0.02052019	0.48560843
Bipolar	IQ	All	0.01694543	0.0198925	0.3942971
SZspecific	EA	rs10046758	-0.0160715	0.00912691	0.07825675
SZspecific	EA	rs118031494	-0.0142555	0.00908717	0.11670517
SZspecific	EA	rs12071594	-0.0139564	0.00896709	0.11961249
SZspecific	EA	rs1278493	-0.0156677	0.00916853	0.08747789

SZspecific	EA	rs12877581	-0.0170731	0.00901897	0.05835539
SZspecific	EA	rs13107325	-0.0129534	0.00876504	0.1394492
SZspecific	EA	rs1518393	-0.0166685	0.00909979	0.06698904
SZspecific	EA	rs16851037	-0.0168673	0.00903729	0.06198399
SZspecific	EA	rs17273111	-0.0165874	0.00915209	0.06992184
SZspecific	EA	rs1783976	-0.0128038	0.00873715	0.14280038
SZspecific	EA	rs28758902	-0.015047	0.00913062	0.09935918
SZspecific	EA	rs35746395	-0.0159721	0.00916342	0.08132911
SZspecific	EA	rs37658	-0.0150469	0.00917134	0.10087254
SZspecific	EA	rs380582	-0.0166701	0.0090939	0.06678593
SZspecific	EA	rs4766500	-0.0151709	0.00926336	0.10147696
SZspecific	EA	rs4814411	-0.015796	0.00915257	0.08437388
SZspecific	EA	rs4950119	-0.0209236	0.00816453	0.01038496
SZspecific	EA	rs605765	-0.0160276	0.00913296	0.07927401
SZspecific	EA	rs61833239	-0.0170978	0.0089783	0.05686509
SZspecific	EA	rs61937595	-0.0153502	0.00922763	0.09621049
SZspecific	EA	rs62039173	-0.0155992	0.00914279	0.08797462
SZspecific	EA	rs6688934	-0.0166857	0.0090836	0.06622356
SZspecific	EA	rs7048	-0.014442	0.00908115	0.11176131
SZspecific	EA	rs71367545	-0.0127515	0.00865616	0.14072219
SZspecific	EA	rs7245983	-0.0132408	0.00874137	0.12984053
SZspecific	EA	rs763263	-0.0142528	0.00904013	0.11488412
SZspecific	EA	rs778370	-0.0187605	0.00841186	0.02573128
SZspecific	EA	rs7838316	-0.0156922	0.00920237	0.08815146
SZspecific	EA	rs7856690	-0.016647	0.00908491	0.0668952
SZspecific	EA	All	-0.0156568	0.00885019	0.0768794
SZspecific	IQ	rs10046758	-0.0450127	0.01289645	0.00048245
SZspecific	IQ	rs118031494	-0.0397579	0.01335245	0.00290539
SZspecific	IQ	rs12071594	-0.0405404	0.0133977	0.00247874
SZspecific	IQ	rs1233578	-0.0368149	0.01320518	0.00530492
SZspecific	IQ	rs1278493	-0.0394668	0.01325372	0.00290338
SZspecific	IQ	rs12877581	-0.0434481	0.01330077	0.00108854
SZspecific	IQ	rs13107325	-0.0346903	0.01134279	0.00222556
SZspecific	IQ	rs1518393	-0.0413078	0.01345273	0.00213635
SZspecific	IQ	rs16851037	-0.0428421	0.01339407	0.00138103
SZspecific	IQ	rs17273111	-0.0433707	0.01342212	0.00123234
SZspecific	IQ	rs1783976	-0.0405363	0.01343145	0.00254438
SZspecific	IQ	rs28758902	-0.0413522	0.01344001	0.00209235
SZspecific	IQ	rs35746395	-0.0414484	0.01345376	0.00206445
SZspecific	IQ	rs37658	-0.0391731	0.01324981	0.00311148
SZspecific	IQ	rs380582	-0.04185	0.01345673	0.00187109
SZspecific	IQ	rs4766500	-0.0404931	0.01357346	0.00285197
SZspecific	IQ	rs4814411	-0.0427132	0.01340322	0.00143865
SZspecific	IQ	rs4950119	-0.0459698	0.01310677	0.00045262
SZspecific	IQ	rs605765	-0.044513	0.01305104	0.00064799
SZspecific	IQ	rs61937595	-0.0413251	0.01351082	0.00222322
SZspecific	IQ	rs62039173	-0.0413356	0.01342996	0.0020849
SZspecific	IQ	rs62062288	-0.0472069	0.01239675	0.00014009
SZspecific	IQ	rs6688934	-0.0417723	0.01344961	0.00189741
SZspecific	IQ	rs7048	-0.0424233	0.01344465	0.00160276

SZspecific	IQ	rs71367545	-0.0394549	0.01320715	0.00281373
SZspecific	IQ	rs7245983	-0.0417897	0.01344863	0.00188767
SZspecific	IQ	rs763263	-0.0413405	0.01347894	0.00216189
SZspecific	IQ	rs778370	-0.0445471	0.0130618	0.00064847
SZspecific	IQ	rs7838316	-0.0444757	0.01325135	0.00078989
SZspecific	IQ	rs7856690	-0.0425157	0.01342601	0.00154199
SZspecific	IQ	All	-0.0417888	0.0130277	0.00133807
PSYshared	EA	rs10255167	0.03750616	0.01089855	0.00057873
PSYshared	EA	rs10737496	0.0357534	0.0108183	0.0009501
PSYshared	EA	rs10994415	0.03816384	0.01089741	0.0004616
PSYshared	EA	rs112481526	0.03548552	0.01079455	0.00101133
PSYshared	EA	rs113779084	0.03542129	0.01090041	0.00115593
PSYshared	EA	rs11764361	0.03656478	0.01091022	0.00080397
PSYshared	EA	rs12575685	0.03758579	0.01092282	0.0005795
PSYshared	EA	rs12668848	0.03603487	0.01087173	0.00091791
PSYshared	EA	rs12868163	0.03954801	0.01052182	0.00017082
PSYshared	EA	rs12932628	0.03909246	0.01061966	0.00023219
PSYshared	EA	rs13044225	0.03690818	0.01090733	0.00071491
PSYshared	EA	rs13195402	0.04008452	0.01077668	0.00019957
PSYshared	EA	rs1487445	0.02850368	0.00840781	0.00069856
PSYshared	EA	rs17183814	0.03695025	0.01091065	0.00070758
PSYshared	EA	rs174592	0.03936244	0.01078153	0.00026131
PSYshared	EA	rs2159100	0.03804454	0.01097873	0.00052965
PSYshared	EA	rs2273738	0.03496072	0.01077522	0.00117637
PSYshared	EA	rs228768	0.0357651	0.01086045	0.00099072
PSYshared	EA	rs237460	0.03692967	0.01091423	0.00071535
PSYshared	EA	rs2577831	0.03830517	0.01091299	0.00044802
PSYshared	EA	rs2693698	0.03797891	0.01084152	0.00045988
PSYshared	EA	rs28455634	0.03806856	0.01088314	0.00046887
PSYshared	EA	rs28565152	0.03585828	0.01086254	0.00096308
PSYshared	EA	rs4447398	0.0372628	0.01091337	0.00063918
PSYshared	EA	rs5758064	0.03599257	0.01084106	0.0009001
PSYshared	EA	rs61554907	0.03887346	0.01068601	0.00027499
PSYshared	EA	rs62581014	0.03703332	0.01092013	0.00069565
PSYshared	EA	rs67712855	0.03423719	0.01056644	0.00119454
PSYshared	EA	rs6887473	0.03738318	0.01090409	0.00060724
PSYshared	EA	rs6954854	0.03824869	0.01084154	0.00041875
PSYshared	EA	rs6992333	0.03878169	0.01076939	0.00031687
PSYshared	EA	rs7201930	0.03741097	0.01090221	0.00060024
PSYshared	EA	rs72809838	0.03733872	0.01094467	0.00064586
PSYshared	EA	rs72841199	0.03609827	0.01091522	0.0009425
PSYshared	EA	rs73560982	0.03742851	0.01090543	0.00059893
PSYshared	EA	rs748455	0.0384592	0.01084274	0.00038964
PSYshared	EA	rs9834970	0.03472403	0.0109473	0.00151431
PSYshared	EA	All	0.03698003	0.01064281	0.00051152
PSYshared	IQ	rs10255167	0.00569399	0.01754068	0.74547148
PSYshared	IQ	rs10737496	0.00390352	0.01732024	0.8216889
PSYshared	IQ	rs10994415	0.00581091	0.01764646	0.74193198
PSYshared	IQ	rs112481526	0.00553502	0.0175374	0.75229658
PSYshared	IQ	rs113779084	0.00209179	0.01725133	0.90348979

PSYshared	IQ	rs11764361	0.00757033	0.01752846	0.66582295
PSYshared	IQ	rs12575685	0.00826957	0.017484	0.63622775
PSYshared	IQ	rs12668848	0.00747237	0.01752796	0.66988119
PSYshared	IQ	rs12868163	0.00549789	0.01752908	0.75379145
PSYshared	IQ	rs12932628	0.00671127	0.01740389	0.69977884
PSYshared	IQ	rs13044225	0.00723393	0.01753194	0.6798891
PSYshared	IQ	rs13195402	0.01202426	0.0169696	0.47858725
PSYshared	IQ	rs1487445	-0.0070179	0.01378889	0.61078463
PSYshared	IQ	rs17183814	0.00829504	0.01737155	0.63300098
PSYshared	IQ	rs174592	0.00312689	0.01743764	0.85768761
PSYshared	IQ	rs2159100	0.0085121	0.01762416	0.62911056
PSYshared	IQ	rs2273738	0.00384003	0.01749314	0.82624802
PSYshared	IQ	rs228768	0.00613183	0.01759955	0.72753353
PSYshared	IQ	rs237460	0.00655273	0.01756176	0.70905556
PSYshared	IQ	rs2577831	0.01269913	0.01672224	0.44760406
PSYshared	IQ	rs2693698	0.00703019	0.01752713	0.68834391
PSYshared	IQ	rs28455634	0.00525125	0.01756563	0.76497809
PSYshared	IQ	rs28565152	0.01051846	0.01696653	0.53528831
PSYshared	IQ	rs41315395	0.00689164	0.01752093	0.69407001
PSYshared	IQ	rs4447398	0.00780793	0.01751153	0.65568828
PSYshared	IQ	rs5758064	0.00657857	0.01753975	0.7076112
PSYshared	IQ	rs61554907	0.00667057	0.01754534	0.70380401
PSYshared	IQ	rs62581014	0.00819047	0.0175217	0.6401801
PSYshared	IQ	rs67712855	0.0030725	0.01730185	0.85905107
PSYshared	IQ	rs6887473	0.00520314	0.01749343	0.76613549
PSYshared	IQ	rs6954854	0.01053321	0.01702189	0.53604567
PSYshared	IQ	rs6992333	0.00458656	0.01749058	0.79314386
PSYshared	IQ	rs7201930	0.00694042	0.01754886	0.69248027
PSYshared	IQ	rs72809838	0.007586	0.01756764	0.66587465
PSYshared	IQ	rs72841199	0.00574667	0.01759734	0.74399697
PSYshared	IQ	rs73560982	0.00947118	0.01715141	0.5808045
PSYshared	IQ	rs748455	0.00771608	0.01756685	0.66048702
PSYshared	IQ	rs9834970	0.00374601	0.01771734	0.8325503
PSYshared	IQ	All	0.00641543	0.01712697	0.70797255
