# Supplementary material for: Splitting schizophrenia: divergent cognitive and educational outcomes revealed by genomic structural equation modelling
Source: Mol Psychiatry. 2026 Jan 31;31(6):3098–107. doi: 10.1038/s41380-026-03444-3 (PMC13190233; doi:10.1038/s41380-026-03444-3)
Supplement: Supplementary file 13 — Supplemental table 11 [file 41380_2026_3444_MOESM13_ESM.pdf]

MR estimates when the exposures are genetic components of years of education (Lee et al.2018) and IQ (Savage et al., 2018) and the outcomes are components of psychotic disorders

Exposure	Outcome	method	nsnp	b	lower_b	upper_b	se	OR	lower_OR	upper_OR	pval
EA	Schizophrenia	Inverse variance weighted	301	0.06897	-0.12530	0.26323	0.09912	1.07140	0.88223	1.30113	0.48654
	Schizophrenia	Weighted median	301	-0.04210	-0.20342	0.11922	0.08231	0.95877	0.81593	1.12661	0.60897
	Schizophrenia	Penalised weighted median	301	-0.08193	-0.24317	0.07931	0.08227	0.92133	0.78413	1.08254	0.31927
	Schizophrenia	MR Egger	301	0.24248	-0.51898	1.00393	0.38850	1.27440	0.59513	2.72898	0.53301
	Schizophrenia	Constrained Maximum Likelihood	284	-0.05517	-0.14778	0.03745	0.04725	0.94633	0.86262	1.03816	0.24302
	Bipolar	Inverse variance weighted	261	0.29214	0.12901	0.45526	0.08323	1.33929	1.13770	1.57659	0.00045
	Bipolar	Weighted median	261	0.16515	-0.00151	0.33181	0.08503	1.17957	0.99849	1.39349	0.05211
	Bipolar	Penalised weighted median	261	0.08317	-0.09061	0.25694	0.08866	1.08672	0.91338	1.29297	0.34822
	Bipolar	MR Egger	261	0.19674	-0.44788	0.84135	0.32888	1.21742	0.63898	2.31949	0.55023
	Bipolar	Constrained Maximum Likelihood	306	0.29458	0.19772	0.39144	0.04942	1.34256	1.21862	1.47910	2.51E-09
	SZspecific	Inverse variance weighted	300	-0.14336	-0.43925	0.15253	0.15096	0.86644	0.64452	1.16477	0.34229
	SZspecific	Weighted median	300	-0.25603	-0.54221	0.03015	0.14601	0.77412	0.58146	1.03061	0.07952
	SZspecific	Penalised weighted median	300	-0.28710	-0.57372	-0.00048	0.14623	0.75044	0.56343	0.99952	0.04961
	SZspecific	MR Egger	300	0.51909	-0.64749	1.68567	0.59519	1.68050	0.52336	5.39606	0.38383
	SZspecific	Constrained Maximum Likelihood	296	-0.26490	-0.44149	-0.08831	0.09010	0.76728	0.64308	0.91548	0.00328
	PSYshared	Inverse variance weighted	300	0.28708	0.09013	0.48403	0.10049	1.33253	1.09431	1.62260	0.00428
	PSYshared	Weighted median	300	0.15227	-0.05564	0.36019	0.10608	1.16447	0.94587	1.43359	0.15116
	PSYshared	Penalised weighted median	300	0.05899	-0.14836	0.26634	0.10579	1.06076	0.86212	1.30517	0.57711
	PSYshared	MR Egger	300	-0.03488	-0.80997	0.74020	0.39545	0.96572	0.44487	2.09635	0.92977
	PSYshared	Constrained Maximum Likelihood	303	0.11889	-0.00263	0.24040	0.06200	1.12624	0.99737	1.27176	0.05516
IQ	Schizophrenia	Inverse variance weighted	51	-0.47496	-0.77196	-0.17797	0.15153	0.62191	0.46211	0.83697	0.00172
	Schizophrenia	Weighted median	51	-0.32490	-0.57289	-0.07691	0.12652	0.72260	0.56389	0.92597	0.01023
	Schizophrenia	Penalised weighted median	51	-0.32420	-0.57279	-0.07562	0.12683	0.72310	0.56395	0.92717	0.01058
	Schizophrenia	MR Egger	51	0.44531	-0.75390	1.64452	0.61184	1.56097	0.47053	5.17852	0.47019
	Schizophrenia	Constrained Maximum Likelihood	46	-0.49770	-0.66776	-0.32765	0.08676	0.60792	0.51286	0.72062	9.68E-09
	Bipolar	Inverse variance weighted	45	-0.13286	-0.40516	0.13943	0.13893	0.87559	0.66687	1.14962	0.33890
	Bipolar	Weighted median	45	-0.09476	-0.36105	0.17154	0.13586	0.90960	0.69694	1.18713	0.48554
	Bipolar	Penalised weighted median	45	-0.07797	-0.34270	0.18675	0.13507	0.92499	0.70985	1.20533	0.56373
	Bipolar	MR Egger	45	-0.15211	-1.42523	1.12101	0.64955	0.85890	0.24045	3.06796	0.81596
	Bipolar	Constrained Maximum Likelihood	47	-0.18660	-0.34941	-0.02379	0.08307	0.82977	0.70510	0.97649	0.02468
	SZspecific	Inverse variance weighted	50	-0.64352	-1.08033	-0.20671	0.22286	0.52544	0.33948	0.81325	0.00388
	SZspecific	Weighted median	50	-0.43678	-0.90676	0.03320	0.23978	0.64611	0.40383	1.03376	0.06852
	SZspecific	Penalised weighted median	50	-0.54177	-1.00219	-0.08135	0.23491	0.58172	0.36707	0.92187	0.02109
	SZspecific	MR Egger	50	1.20111	-0.78635	3.18856	1.01401	3.32380	0.45551	24.25355	0.24204
	SZspecific	Constrained Maximum Likelihood	49	-0.66388	-0.97143	-0.35632	0.15692	0.51485	0.37854	0.70025	0.00002
	PSYshared	Inverse variance weighted	50	-0.19350	-0.52376	0.13676	0.16850	0.82407	0.59229	1.14655	0.25081
	PSYshared	Weighted median	50	-0.14160	-0.46473	0.18153	0.16486	0.86797	0.62830	1.19905	0.39040
	PSYshared	Penalised weighted median	50	-0.11086	-0.43091	0.20918	0.16329	0.89506	0.64992	1.23267	0.49718
	PSYshared	MR Egger	50	-0.00859	-1.56087	1.54369	0.79198	0.99145	0.20995	4.68184	0.99139
	PSYshared	Constrained Maximum Likelihood	47	-0.24728	-0.45834	-0.03623	0.10768	0.78092	0.63233	0.96442	0.02165

Heterogeneity tests

Exposure	Outcome	Method	Q	Q_df	Q_pval
**EA**	**Schizophrenia**	MR Egger	1678.37	299	2.14E-190
**EA**	**Schizophrenia**	Inverse variance weighted	1679.57	300	3.10E-190
**EA**	**Bipolar**	MR Egger	760.09	259	9.88E-51
**EA**	**Bipolar**	Inverse variance weighted	760.35	260	1.56E-50
**EA**	**SZspecific**	MR Egger	991.64	298	2.09E-75
**EA**	**SZspecific**	Inverse variance weighted	996.05	299	8.16E-76
**EA**	**PSYshared**	MR Egger	869.16	298	2.96E-57
**EA**	**PSYshared**	Inverse variance weighted	871.23	299	2.56E-57
**IQ**	**Schizophrenia**	MR Egger	237.85	49	1.30E-26
**IQ**	**Schizophrenia**	Inverse variance weighted	249.53	50	2.64E-28
**IQ**	**Bipolar**	MR Egger	141.36	43	2.07E-12
**IQ**	**Bipolar**	Inverse variance weighted	141.36	44	3.81E-12
**IQ**	**SZspecific**	MR Egger	124.04	48	1.19E-08
**IQ**	**SZspecific**	Inverse variance weighted	133.00	49	1.09E-09
**IQ**	**PSYshared**	MR Egger	149.85	48	2.09E-12
**IQ**	**PSYshared**	Inverse variance weighted	150.03	49	3.52E-12

	Min_F	Mean_F	Max_F	I_squared
**EA_Schizophrenia**	29.69	49.18	240.25	0.98
**EA_Bipolar**	29.69	49.18	240.25	0.98
**EA_SZspecific**	29.69	49.22	240.25	0.98
**EA_PSYshared**	29.69	49.22	240.25	0.98
**IQ_Schizophrenia**	29.76	42.40	125.64	0.98
**IQ_Bipolar**	29.76	42.44	125.64	0.98
**IQ_SZspecific**	29.76	42.44	125.64	0.98
**IQ_PSYshared**	29.76	42.44	125.64	0.98

Tests for pleiotropy

Exposure	Outcome	Egger Intercept	SE	pval
**EA**	**Schizophrenia**	-0.0025	0.0053	0.6445
**EA**	**Bipolar**	0.0013	0.0045	0.7645
**EA**	**SZspecific**	-0.0094	0.0082	0.2508
**EA**	**PSYshared**	0.0046	0.0054	0.4006
**IQ**	**Schizophrenia**	-0.0190	0.0123	0.1273
**IQ**	**Bipolar**	0.0004	0.0130	0.9759
**IQ**	**SZspecific**	-0.0374	0.0201	0.0687
**IQ**	**PSYshared**	-0.0038	0.0157	0.8121
