# Supplementary material for: Splitting schizophrenia: divergent cognitive and educational outcomes revealed by genomic structural equation modelling
Source: Mol Psychiatry. 2026 Jan 31;31(6):3098–107. doi: 10.1038/s41380-026-03444-3 (PMC13190233; doi:10.1038/s41380-026-03444-3)
Supplement: Supplementary file 14 — Supplemental table 12 [file 41380_2026_3444_MOESM14_ESM.pdf]

| exposure | outcome | SNP | b | se | p |
|---|---|---|---|---|---|
| EA | Schizophrenia | rs10073890 | 0.0665 | 0.0994 | 0.503215191 |
| EA | Schizophrenia | rs1008078 | 0.0807 | 0.0993 | 0.416554302 |
| EA | Schizophrenia | rs10189857 | 0.0530 | 0.0990 | 0.592587675 |
| EA | Schizophrenia | rs10205801 | 0.0538 | 0.0979 | 0.582461989 |
| EA | Schizophrenia | rs10215082 | 0.0724 | 0.0994 | 0.466561446 |
| EA | Schizophrenia | rs10240905 | 0.0684 | 0.0994 | 0.49152724 |
| EA | Schizophrenia | rs10456918 | 0.0629 | 0.0992 | 0.526065943 |
| EA | Schizophrenia | rs10460095 | 0.0746 | 0.0992 | 0.452086626 |
| EA | Schizophrenia | rs1051474 | 0.0755 | 0.0992 | 0.446842622 |
| EA | Schizophrenia | rs10760023 | 0.0698 | 0.0994 | 0.482513114 |
| EA | Schizophrenia | rs10765775 | 0.0658 | 0.0995 | 0.50861606 |
| EA | Schizophrenia | rs10772644 | 0.0666 | 0.0994 | 0.502441605 |
| EA | Schizophrenia | rs10773002 | 0.0409 | 0.0982 | 0.676735469 |
| EA | Schizophrenia | rs10856785 | 0.0690 | 0.0994 | 0.487417677 |
| EA | Schizophrenia | rs10862376 | 0.0739 | 0.0993 | 0.456876047 |
| EA | Schizophrenia | rs10875121 | 0.0470 | 0.0976 | 0.629662182 |
| EA | Schizophrenia | rs10887801 | 0.0693 | 0.0994 | 0.485876274 |
| EA | Schizophrenia | rs10940921 | 0.0704 | 0.0994 | 0.478831596 |
| EA | Schizophrenia | rs10963297 | 0.0729 | 0.0996 | 0.464233322 |
| EA | Schizophrenia | rs10994777 | 0.0684 | 0.0994 | 0.491728313 |
| EA | Schizophrenia | rs11023749 | 0.0692 | 0.0994 | 0.486375198 |
| EA | Schizophrenia | rs1105307 | 0.0759 | 0.0991 | 0.443885071 |
| EA | Schizophrenia | rs1106090 | 0.0571 | 0.0987 | 0.562833867 |
| EA | Schizophrenia | rs11081529 | 0.0716 | 0.0994 | 0.47108922 |
| EA | Schizophrenia | rs11123818 | 0.0700 | 0.0998 | 0.482831925 |
| EA | Schizophrenia | rs111821073 | 0.0732 | 0.0993 | 0.460962446 |
| EA | Schizophrenia | rs11222609 | 0.0701 | 0.0994 | 0.480614226 |
| EA | Schizophrenia | rs112687095 | 0.0678 | 0.0994 | 0.495226664 |
| EA | Schizophrenia | rs112806496 | 0.0715 | 0.0994 | 0.471740184 |
| EA | Schizophrenia | rs113182709 | 0.0716 | 0.0993 | 0.470804738 |
| EA | Schizophrenia | rs113520408 | 0.0758 | 0.0992 | 0.444561315 |
| EA | Schizophrenia | rs113615161 | 0.0652 | 0.0993 | 0.511376457 |
| EA | Schizophrenia | rs1143770 | 0.0712 | 0.0994 | 0.473750798 |
| EA | Schizophrenia | rs115000530 | 0.0690 | 0.0995 | 0.488001753 |
| EA | Schizophrenia | rs11601122 | 0.0692 | 0.0995 | 0.486683068 |
| EA | Schizophrenia | rs11620355 | 0.0734 | 0.0993 | 0.45983919 |
| EA | Schizophrenia | rs11627087 | 0.0713 | 0.0993 | 0.472633034 |
| EA | Schizophrenia | rs11635092 | 0.0700 | 0.0994 | 0.481691246 |
| EA | Schizophrenia | rs11657342 | 0.0743 | 0.0993 | 0.45427489 |
| EA | Schizophrenia | rs11663602 | 0.0832 | 0.0982 | 0.396567728 |
| EA | Schizophrenia | rs11678980 | 0.0813 | 0.0992 | 0.412407905 |
| EA | Schizophrenia | rs11681861 | 0.0651 | 0.0993 | 0.512033146 |
| EA | Schizophrenia | rs11694904 | 0.0621 | 0.0992 | 0.531255953 |
| EA | Schizophrenia | rs11732657 | 0.0670 | 0.0994 | 0.500080397 |
| EA | Schizophrenia | rs117468730 | 0.0693 | 0.0994 | 0.48563817 |
| EA | Schizophrenia | rs11752914 | 0.0626 | 0.0991 | 0.527229365 |
| EA | Schizophrenia | rs11772580 | 0.0665 | 0.0994 | 0.5032615 |

| | | | | | |
|---|---|---|---|---|---|
| EA | Schizophrenia | rs117799466 | 0.0830 | 0.0981 | 0.397491366 |
| EA | Schizophrenia | rs117895796 | 0.0717 | 0.0993 | 0.47064411 |
| EA | Schizophrenia | rs11871429 | 0.0667 | 0.0994 | 0.502191861 |
| EA | Schizophrenia | rs12028010 | 0.0716 | 0.0995 | 0.472050575 |
| EA | Schizophrenia | rs12332731 | 0.0682 | 0.0994 | 0.492789649 |
| EA | Schizophrenia | rs12375949 | 0.0699 | 0.0995 | 0.482604413 |
| EA | Schizophrenia | rs12468040 | 0.0731 | 0.0994 | 0.462253046 |
| EA | Schizophrenia | rs12503522 | 0.0652 | 0.0993 | 0.511693417 |
| EA | Schizophrenia | rs12519073 | 0.0686 | 0.0994 | 0.489844793 |
| EA | Schizophrenia | rs12574281 | 0.0680 | 0.0994 | 0.493655159 |
| EA | Schizophrenia | rs12602286 | 0.0562 | 0.0985 | 0.56816143 |
| EA | Schizophrenia | rs12643771 | 0.0593 | 0.0992 | 0.549710143 |
| EA | Schizophrenia | rs12647336 | 0.0683 | 0.0994 | 0.491714539 |
| EA | Schizophrenia | rs12655753 | 0.0708 | 0.0994 | 0.47628156 |
| EA | Schizophrenia | rs12682775 | 0.0696 | 0.0994 | 0.483658657 |
| EA | Schizophrenia | rs12804787 | 0.0718 | 0.0993 | 0.469634568 |
| EA | Schizophrenia | rs1291818 | 0.0747 | 0.0992 | 0.451475207 |
| EA | Schizophrenia | rs12940014 | 0.0745 | 0.0991 | 0.45267851 |
| EA | Schizophrenia | rs13010566 | 0.0688 | 0.0994 | 0.48891859 |
| EA | Schizophrenia | rs13029509 | 0.0758 | 0.0991 | 0.444173444 |
| EA | Schizophrenia | rs13090388 | 0.0581 | 0.1000 | 0.561049574 |
| EA | Schizophrenia | rs13141210 | 0.0739 | 0.0994 | 0.457453974 |
| EA | Schizophrenia | rs13145650 | 0.0626 | 0.0991 | 0.527952999 |
| EA | Schizophrenia | rs1334297 | 0.0708 | 0.0998 | 0.478362652 |
| EA | Schizophrenia | rs13422673 | 0.0697 | 0.0994 | 0.48309411 |
| EA | Schizophrenia | rs13428598 | 0.0742 | 0.0995 | 0.45585553 |
| EA | Schizophrenia | rs1363862 | 0.0637 | 0.0992 | 0.520676935 |
| EA | Schizophrenia | rs1391438 | 0.0628 | 0.0995 | 0.528084915 |
| EA | Schizophrenia | rs1427298 | 0.0623 | 0.0991 | 0.52923021 |
| EA | Schizophrenia | rs1450782 | 0.0710 | 0.0993 | 0.474994077 |
| EA | Schizophrenia | rs152603 | 0.0623 | 0.0991 | 0.52963042 |
| EA | Schizophrenia | rs1558727 | 0.0721 | 0.0994 | 0.46797409 |
| EA | Schizophrenia | rs1566085 | 0.0626 | 0.0995 | 0.529126439 |
| EA | Schizophrenia | rs1569092 | 0.0725 | 0.0994 | 0.465987956 |
| EA | Schizophrenia | rs1582173 | 0.0637 | 0.0992 | 0.521017522 |
| EA | Schizophrenia | rs1584469 | 0.0700 | 0.0994 | 0.481571698 |
| EA | Schizophrenia | rs1592757 | 0.0632 | 0.0992 | 0.523855691 |
| EA | Schizophrenia | rs1595973 | 0.0679 | 0.0994 | 0.494242359 |
| EA | Schizophrenia | rs1618725 | 0.0703 | 0.0995 | 0.4799423 |
| EA | Schizophrenia | rs1620977 | 0.0636 | 0.0996 | 0.523150897 |
| EA | Schizophrenia | rs1671770 | 0.0720 | 0.0993 | 0.468664074 |
| EA | Schizophrenia | rs16846463 | 0.0705 | 0.0995 | 0.478480009 |
| EA | Schizophrenia | rs16854920 | 0.0637 | 0.0992 | 0.520455697 |
| EA | Schizophrenia | rs1689510 | 0.0671 | 0.0996 | 0.500259101 |
| EA | Schizophrenia | rs16995054 | 0.0705 | 0.0994 | 0.478229441 |
| EA | Schizophrenia | rs17048855 | 0.0756 | 0.0992 | 0.445667567 |
| EA | Schizophrenia | rs17126938 | 0.0676 | 0.0994 | 0.496655012 |
| EA | Schizophrenia | rs17425572 | 0.0687 | 0.0995 | 0.489922473 |
| EA | Schizophrenia | rs17489649 | 0.0645 | 0.0994 | 0.51666089 |
| EA | Schizophrenia | rs175325 | 0.0738 | 0.0993 | 0.457464304 |
| EA | Schizophrenia | rs17551064 | 0.0656 | 0.0994 | 0.509258807 |
| EA | Schizophrenia | rs17563464 | 0.0681 | 0.0994 | 0.493204259 |
| EA | Schizophrenia | rs17565975 | 0.0703 | 0.0994 | 0.47957267 |
| EA | Schizophrenia | rs17598675 | 0.0678 | 0.0994 | 0.495534848 |
| EA | Schizophrenia | rs176218 | 0.0759 | 0.0994 | 0.444894862 |
| EA | Schizophrenia | rs1827540 | 0.0644 | 0.0993 | 0.516347034 |

| | | | | | |
|---|---|---|---|---|---|
| EA | Schizophrenia | rs1866823 | 0.0741 | 0.0992 | 0.454825603 |
| EA | Schizophrenia | rs1882273 | 0.0786 | 0.0989 | 0.427039493 |
| EA | Schizophrenia | rs192436652 | 0.0634 | 0.0992 | 0.522850978 |
| EA | Schizophrenia | rs1925576 | 0.0664 | 0.0993 | 0.504101701 |
| EA | Schizophrenia | rs1947114 | 0.0681 | 0.0994 | 0.493243497 |
| EA | Schizophrenia | rs1964927 | 0.0674 | 0.0995 | 0.497850386 |
| EA | Schizophrenia | rs2016392 | 0.0731 | 0.0993 | 0.461395744 |
| EA | Schizophrenia | rs2052285 | 0.0730 | 0.0993 | 0.462185438 |
| EA | Schizophrenia | rs2067854 | 0.0540 | 0.0983 | 0.583096596 |
| EA | Schizophrenia | rs2179152 | 0.0710 | 0.0995 | 0.475455935 |
| EA | Schizophrenia | rs2182505 | 0.0687 | 0.0994 | 0.489222533 |
| EA | Schizophrenia | rs225291 | 0.0663 | 0.0993 | 0.504697285 |
| EA | Schizophrenia | rs2256965 | 0.0697 | 0.0994 | 0.483313256 |
| EA | Schizophrenia | rs2283076 | 0.0734 | 0.0992 | 0.4591501 |
| EA | Schizophrenia | rs2287838 | 0.0741 | 0.0993 | 0.455611538 |
| EA | Schizophrenia | rs2302761 | 0.0678 | 0.0994 | 0.495070589 |
| EA | Schizophrenia | rs2347526 | 0.0644 | 0.0994 | 0.516982518 |
| EA | Schizophrenia | rs242093 | 0.0702 | 0.0994 | 0.480083532 |
| EA | Schizophrenia | rs2447535 | 0.0736 | 0.0993 | 0.458816391 |
| EA | Schizophrenia | rs2554835 | 0.0723 | 0.0993 | 0.466565079 |
| EA | Schizophrenia | rs2570497 | 0.0668 | 0.0994 | 0.501411113 |
| EA | Schizophrenia | rs2725370 | 0.0702 | 0.0995 | 0.480274741 |
| EA | Schizophrenia | rs277828 | 0.0751 | 0.0991 | 0.448389573 |
| EA | Schizophrenia | rs2819336 | 0.0844 | 0.0991 | 0.394172218 |
| EA | Schizophrenia | rs2820314 | 0.0728 | 0.0993 | 0.463678968 |
| EA | Schizophrenia | rs28373063 | 0.0680 | 0.0994 | 0.493707802 |
| EA | Schizophrenia | rs28513670 | 0.0640 | 0.0993 | 0.519121595 |
| EA | Schizophrenia | rs2885198 | 0.0676 | 0.0994 | 0.496137879 |
| EA | Schizophrenia | rs2901616 | 0.0596 | 0.0987 | 0.545575032 |
| EA | Schizophrenia | rs2905426 | 0.0854 | 0.0974 | 0.380834149 |
| EA | Schizophrenia | rs2923431 | 0.0657 | 0.0994 | 0.508647103 |
| EA | Schizophrenia | rs2971970 | 0.0722 | 0.0995 | 0.467565559 |
| EA | Schizophrenia | rs2998315 | 0.0679 | 0.0995 | 0.494840475 |
| EA | Schizophrenia | rs3013014 | 0.0657 | 0.0993 | 0.508239691 |
| EA | Schizophrenia | rs301800 | 0.0756 | 0.0992 | 0.446365818 |
| EA | Schizophrenia | rs3026996 | 0.0626 | 0.0993 | 0.528579626 |
| EA | Schizophrenia | rs31940 | 0.0710 | 0.0994 | 0.475228383 |
| EA | Schizophrenia | rs337637 | 0.0693 | 0.0994 | 0.485634265 |
| EA | Schizophrenia | rs34316 | 0.0798 | 0.0995 | 0.422952942 |
| EA | Schizophrenia | rs34394051 | 0.0634 | 0.0992 | 0.522947833 |
| EA | Schizophrenia | rs34485537 | 0.0715 | 0.0994 | 0.471798847 |
| EA | Schizophrenia | rs34853711 | 0.0643 | 0.0994 | 0.517515351 |
| EA | Schizophrenia | rs35039375 | 0.0694 | 0.0994 | 0.485149215 |
| EA | Schizophrenia | rs35309068 | 0.0650 | 0.0994 | 0.513403376 |
| EA | Schizophrenia | rs35316276 | 0.0715 | 0.0994 | 0.471761078 |
| EA | Schizophrenia | rs35417702 | 0.0528 | 0.0986 | 0.592178259 |
| EA | Schizophrenia | rs35475880 | 0.0643 | 0.0993 | 0.517344259 |
| EA | Schizophrenia | rs35532491 | 0.0682 | 0.0994 | 0.492775343 |
| EA | Schizophrenia | rs36083520 | 0.0641 | 0.0993 | 0.518825895 |
| EA | Schizophrenia | rs36119825 | 0.0655 | 0.0993 | 0.509769261 |
| EA | Schizophrenia | rs363096 | 0.0744 | 0.0994 | 0.4538488 |
| EA | Schizophrenia | rs3747631 | 0.0832 | 0.0992 | 0.401884005 |
| EA | Schizophrenia | rs3788556 | 0.0825 | 0.0984 | 0.401733803 |
| EA | Schizophrenia | rs3800546 | 0.0660 | 0.0994 | 0.506611609 |
| EA | Schizophrenia | rs3809634 | 0.0698 | 0.0994 | 0.482741891 |
| EA | Schizophrenia | rs3890802 | 0.0578 | 0.0985 | 0.557342457 |
| EA | Schizophrenia | rs3897821 | 0.0557 | 0.0989 | 0.573346817 |
| EA | Schizophrenia | rs406413 | 0.0755 | 0.0994 | 0.447374387 |
| EA | Schizophrenia | rs4073894 | 0.0738 | 0.0993 | 0.457458157 |

| | | | | | |
|---|---|---|---|---|---|
| EA | Schizophrenia | rs4328757 | 0.0622 | 0.0991 | 0.530109564 |
| EA | Schizophrenia | rs4352658 | 0.0678 | 0.0994 | 0.4950611 |
| EA | Schizophrenia | rs4369924 | 0.0649 | 0.0993 | 0.513003337 |
| EA | Schizophrenia | rs4382592 | 0.0694 | 0.0996 | 0.485663385 |
| EA | Schizophrenia | rs4384309 | 0.0686 | 0.0994 | 0.490437722 |
| EA | Schizophrenia | rs4442732 | 0.0746 | 0.0992 | 0.452046113 |
| EA | Schizophrenia | rs4667029 | 0.0633 | 0.0991 | 0.523169479 |
| EA | Schizophrenia | rs4700393 | 0.0899 | 0.0991 | 0.364027213 |
| EA | Schizophrenia | rs4726070 | 0.0693 | 0.0995 | 0.486138059 |
| EA | Schizophrenia | rs4733264 | 0.0684 | 0.0994 | 0.49150909 |
| EA | Schizophrenia | rs4757957 | 0.0665 | 0.0995 | 0.503623618 |
| EA | Schizophrenia | rs4766424 | 0.0728 | 0.0993 | 0.463527901 |
| EA | Schizophrenia | rs4778058 | 0.0677 | 0.0994 | 0.495488625 |
| EA | Schizophrenia | rs4787457 | 0.0756 | 0.0995 | 0.447132433 |
| EA | Schizophrenia | rs4810227 | 0.0716 | 0.0994 | 0.471504218 |
| EA | Schizophrenia | rs4839155 | 0.0677 | 0.0994 | 0.495626536 |
| EA | Schizophrenia | rs4846724 | 0.0658 | 0.0993 | 0.507889068 |
| EA | Schizophrenia | rs4870482 | 0.0710 | 0.0994 | 0.474995671 |
| EA | Schizophrenia | rs4888746 | 0.0712 | 0.0993 | 0.473558027 |
| EA | Schizophrenia | rs4895650 | 0.0735 | 0.0992 | 0.459078553 |
| EA | Schizophrenia | rs4945424 | 0.0653 | 0.0993 | 0.510667091 |
| EA | Schizophrenia | rs4964046 | 0.0657 | 0.0993 | 0.50801643 |
| EA | Schizophrenia | rs4972400 | 0.0644 | 0.0993 | 0.516733886 |
| EA | Schizophrenia | rs4984541 | 0.0678 | 0.0994 | 0.495054977 |
| EA | Schizophrenia | rs532799 | 0.0646 | 0.0993 | 0.514931103 |
| EA | Schizophrenia | rs535307 | 0.0664 | 0.0993 | 0.503637041 |
| EA | Schizophrenia | rs55736314 | 0.0574 | 0.0990 | 0.562287364 |
| EA | Schizophrenia | rs55771711 | 0.0752 | 0.0993 | 0.448973272 |
| EA | Schizophrenia | rs56391344 | 0.0701 | 0.0995 | 0.481051395 |
| EA | Schizophrenia | rs575113 | 0.0660 | 0.0994 | 0.506412799 |
| EA | Schizophrenia | rs59123361 | 0.0701 | 0.0994 | 0.480653042 |
| EA | Schizophrenia | rs59480703 | 0.0645 | 0.0992 | 0.515735491 |
| EA | Schizophrenia | rs60904894 | 0.0696 | 0.0994 | 0.484112284 |
| EA | Schizophrenia | rs6122735 | 0.0653 | 0.0993 | 0.510559336 |
| EA | Schizophrenia | rs6123924 | 0.0709 | 0.0994 | 0.475844018 |
| EA | Schizophrenia | rs613872 | 0.0627 | 0.0993 | 0.527701222 |
| EA | Schizophrenia | rs62097985 | 0.0806 | 0.0989 | 0.415235981 |
| EA | Schizophrenia | rs62103084 | 0.0684 | 0.0994 | 0.491110886 |
| EA | Schizophrenia | rs62157915 | 0.0684 | 0.0994 | 0.491552482 |
| EA | Schizophrenia | rs62183776 | 0.0657 | 0.0993 | 0.50853356 |
| EA | Schizophrenia | rs62184480 | 0.0697 | 0.0995 | 0.483863681 |
| EA | Schizophrenia | rs622169 | 0.0629 | 0.0991 | 0.525135704 |
| EA | Schizophrenia | rs62439690 | 0.0682 | 0.0994 | 0.492838338 |
| EA | Schizophrenia | rs62444881 | 0.0543 | 0.0988 | 0.582857196 |
| EA | Schizophrenia | rs6493265 | 0.0749 | 0.0994 | 0.450904738 |
| EA | Schizophrenia | rs6513959 | 0.0591 | 0.0988 | 0.550146793 |
| EA | Schizophrenia | rs6557171 | 0.0691 | 0.0995 | 0.487793725 |
| EA | Schizophrenia | rs663234 | 0.0705 | 0.0994 | 0.478171047 |
| EA | Schizophrenia | rs66568921 | 0.0664 | 0.0995 | 0.504420541 |
| EA | Schizophrenia | rs6731373 | 0.0633 | 0.0993 | 0.523814056 |
| EA | Schizophrenia | rs6731967 | 0.0646 | 0.0993 | 0.515119562 |
| EA | Schizophrenia | rs67885444 | 0.0652 | 0.0993 | 0.511428884 |
| EA | Schizophrenia | rs67890737 | 0.0589 | 0.0988 | 0.551206937 |
| EA | Schizophrenia | rs6803651 | 0.0717 | 0.0994 | 0.470878124 |
| EA | Schizophrenia | rs6805241 | 0.0673 | 0.0994 | 0.498598084 |
| EA | Schizophrenia | rs6938002 | 0.0663 | 0.0993 | 0.504320578 |
| EA | Schizophrenia | rs6959891 | 0.0702 | 0.0994 | 0.479878612 |
| EA | Schizophrenia | rs7012546 | 0.0707 | 0.0994 | 0.47705445 |
| EA | Schizophrenia | rs7016302 | 0.0730 | 0.0992 | 0.46175751 |

| | | | | | |
|---|---|---|---|---|---|
| EA | Schizophrenia | rs702606 | 0.0709 | 0.0994 | 0.475270644 |
| EA | Schizophrenia | rs7029718 | 0.0801 | 0.0998 | 0.422181836 |
| EA | Schizophrenia | rs7031698 | 0.0704 | 0.0994 | 0.47867375 |
| EA | Schizophrenia | rs710629 | 0.0724 | 0.0993 | 0.466297396 |
| EA | Schizophrenia | rs71646142 | 0.0724 | 0.0993 | 0.466021806 |
| EA | Schizophrenia | rs7233920 | 0.0709 | 0.0994 | 0.475481072 |
| EA | Schizophrenia | rs72486027 | 0.0630 | 0.0991 | 0.525186347 |
| EA | Schizophrenia | rs7257460 | 0.0684 | 0.0994 | 0.491366833 |
| EA | Schizophrenia | rs7278859 | 0.0708 | 0.0994 | 0.476374114 |
| EA | Schizophrenia | rs72807818 | 0.0640 | 0.0994 | 0.519491431 |
| EA | Schizophrenia | rs72828517 | 0.0777 | 0.0992 | 0.43343977 |
| EA | Schizophrenia | rs72840994 | 0.0676 | 0.0994 | 0.496310458 |
| EA | Schizophrenia | rs730384 | 0.0744 | 0.0992 | 0.453242163 |
| EA | Schizophrenia | rs7315713 | 0.0752 | 0.0991 | 0.44790361 |
| EA | Schizophrenia | rs73301698 | 0.0701 | 0.0994 | 0.48070496 |
| EA | Schizophrenia | rs7332724 | 0.0752 | 0.0991 | 0.448067258 |
| EA | Schizophrenia | rs73344830 | 0.0657 | 0.0996 | 0.509164227 |
| EA | Schizophrenia | rs736282 | 0.0676 | 0.0994 | 0.496405476 |
| EA | Schizophrenia | rs73874335 | 0.0718 | 0.0993 | 0.469589825 |
| EA | Schizophrenia | rs743316 | 0.0730 | 0.0993 | 0.462367309 |
| EA | Schizophrenia | rs74643044 | 0.0695 | 0.0994 | 0.484452452 |
| EA | Schizophrenia | rs74701752 | 0.0729 | 0.0993 | 0.462903403 |
| EA | Schizophrenia | rs74998289 | 0.0524 | 0.0986 | 0.59544079 |
| EA | Schizophrenia | rs7543462 | 0.0714 | 0.0993 | 0.472600534 |
| EA | Schizophrenia | rs7594904 | 0.0698 | 0.0994 | 0.482640079 |
| EA | Schizophrenia | rs7603132 | 0.0731 | 0.0993 | 0.461676623 |
| EA | Schizophrenia | rs76076331 | 0.0788 | 0.0990 | 0.426435566 |
| EA | Schizophrenia | rs7650602 | 0.0679 | 0.0994 | 0.494347908 |
| EA | Schizophrenia | rs76608582 | 0.0677 | 0.0994 | 0.495672735 |
| EA | Schizophrenia | rs77025239 | 0.0691 | 0.0994 | 0.487281977 |
| EA | Schizophrenia | rs77128898 | 0.0698 | 0.0994 | 0.482678159 |
| EA | Schizophrenia | rs77702622 | 0.0718 | 0.0994 | 0.469991588 |
| EA | Schizophrenia | rs77719387 | 0.0733 | 0.0993 | 0.459956221 |
| EA | Schizophrenia | rs7773815 | 0.0678 | 0.0994 | 0.495073966 |
| EA | Schizophrenia | rs77835879 | 0.0662 | 0.0993 | 0.504890133 |
| EA | Schizophrenia | rs7796203 | 0.0673 | 0.0994 | 0.498333632 |
| EA | Schizophrenia | rs7803932 | 0.0758 | 0.0991 | 0.444632736 |
| EA | Schizophrenia | rs7808399 | 0.0654 | 0.0993 | 0.51016948 |
| EA | Schizophrenia | rs7833201 | 0.0704 | 0.0994 | 0.478416097 |
| EA | Schizophrenia | rs7863447 | 0.0690 | 0.0995 | 0.487583307 |
| EA | Schizophrenia | rs7864396 | 0.0765 | 0.0989 | 0.439566194 |
| EA | Schizophrenia | rs78721320 | 0.0697 | 0.0994 | 0.483325725 |
| EA | Schizophrenia | rs790647 | 0.0716 | 0.0994 | 0.471433706 |
| EA | Schizophrenia | rs7924036 | 0.0634 | 0.0995 | 0.523831012 |
| EA | Schizophrenia | rs79265434 | 0.0507 | 0.0984 | 0.606872929 |
| EA | Schizophrenia | rs79269403 | 0.0705 | 0.0994 | 0.47851886 |
| EA | Schizophrenia | rs7928622 | 0.0710 | 0.0994 | 0.474958216 |
| EA | Schizophrenia | rs795230 | 0.0712 | 0.0993 | 0.473516838 |
| EA | Schizophrenia | rs79523955 | 0.0730 | 0.0993 | 0.461917492 |
| EA | Schizophrenia | rs7977614 | 0.0762 | 0.0991 | 0.442176196 |
| EA | Schizophrenia | rs7993663 | 0.0735 | 0.0993 | 0.459380332 |
| EA | Schizophrenia | rs8008382 | 0.0630 | 0.0992 | 0.525553929 |
| EA | Schizophrenia | rs80171383 | 0.0727 | 0.0993 | 0.464330646 |
| EA | Schizophrenia | rs8020034 | 0.0737 | 0.0994 | 0.458659772 |
| EA | Schizophrenia | rs818415 | 0.0640 | 0.0992 | 0.518838606 |
| EA | Schizophrenia | rs837080 | 0.0803 | 0.0986 | 0.415403971 |
| EA | Schizophrenia | rs892612 | 0.0640 | 0.0993 | 0.518753526 |
| EA | Schizophrenia | rs894067 | 0.0652 | 0.0993 | 0.511569009 |
| EA | Schizophrenia | rs9289300 | 0.0730 | 0.0993 | 0.462216167 |

| | | | | | |
|---|---|---|---|---|---|
| EA | Schizophrenia | rs9320493 | 0.0724 | 0.0993 | 0.466168265 |
| EA | Schizophrenia | rs9342482 | 0.0720 | 0.0994 | 0.468657423 |
| EA | Schizophrenia | rs9349956 | 0.0705 | 0.0995 | 0.478360522 |
| EA | Schizophrenia | rs9372625 | 0.0606 | 0.0998 | 0.543806901 |
| EA | Schizophrenia | rs9384679 | 0.0734 | 0.0992 | 0.459249982 |
| EA | Schizophrenia | rs9386319 | 0.0692 | 0.0994 | 0.486501795 |
| EA | Schizophrenia | rs9436866 | 0.0663 | 0.0994 | 0.504990354 |
| EA | Schizophrenia | rs9503598 | 0.0713 | 0.0994 | 0.473037374 |
| EA | Schizophrenia | rs9529119 | 0.0688 | 0.0994 | 0.488669946 |
| EA | Schizophrenia | rs9556958 | 0.0645 | 0.0993 | 0.51592428 |
| EA | Schizophrenia | rs9616906 | 0.0608 | 0.0993 | 0.540345957 |
| EA | Schizophrenia | rs9679654 | 0.0671 | 0.0994 | 0.499397481 |
| EA | Schizophrenia | rs969512 | 0.0652 | 0.0994 | 0.512020332 |
| EA | Schizophrenia | rs9704097 | 0.0674 | 0.0994 | 0.497993497 |
| EA | Schizophrenia | rs9882532 | 0.0836 | 0.0983 | 0.3953011 |
| EA | Schizophrenia | rs9914918 | 0.0766 | 0.0990 | 0.439530962 |
| EA | Schizophrenia | rs9933256 | 0.0673 | 0.0994 | 0.49803816 |
| EA | Schizophrenia | rs9936270 | 0.0666 | 0.0994 | 0.502725359 |
| EA | Schizophrenia | rs9938678 | 0.0682 | 0.0994 | 0.492934833 |
| EA | Schizophrenia | rs9964724 | 0.0768 | 0.0996 | 0.440173134 |
| EA | Schizophrenia | rs9995567 | 0.0714 | 0.0993 | 0.472019942 |
| EA | Schizophrenia | All | 0.0690 | 0.0991 | 0.486544668 |
| EA | Bipolar | rs10073890 | 0.2909 | 0.0835 | 0.000494779 |
| EA | Bipolar | rs1008078 | 0.3062 | 0.0831 | 0.000231421 |
| EA | Bipolar | rs10189857 | 0.2830 | 0.0835 | 0.000699711 |
| EA | Bipolar | rs10205801 | 0.2862 | 0.0832 | 0.000585091 |
| EA | Bipolar | rs10215082 | 0.2834 | 0.0832 | 0.000658368 |
| EA | Bipolar | rs10240905 | 0.2868 | 0.0833 | 0.000578183 |
| EA | Bipolar | rs10456918 | 0.2909 | 0.0835 | 0.000496667 |
| EA | Bipolar | rs10460095 | 0.2926 | 0.0835 | 0.000458397 |
| EA | Bipolar | rs1051474 | 0.2904 | 0.0835 | 0.000505573 |
| EA | Bipolar | rs10765775 | 0.2963 | 0.0836 | 0.000390045 |
| EA | Bipolar | rs10856785 | 0.2921 | 0.0835 | 0.000468389 |
| EA | Bipolar | rs10887801 | 0.2963 | 0.0834 | 0.000381281 |
| EA | Bipolar | rs10940921 | 0.2865 | 0.0833 | 0.000581768 |
| EA | Bipolar | rs10994777 | 0.2934 | 0.0835 | 0.000441907 |
| EA | Bipolar | rs11023749 | 0.2913 | 0.0835 | 0.000486133 |
| EA | Bipolar | rs1105307 | 0.2917 | 0.0835 | 0.000476514 |
| EA | Bipolar | rs1106090 | 0.2877 | 0.0834 | 0.000562057 |
| EA | Bipolar | rs11081529 | 0.2954 | 0.0835 | 0.000402302 |
| EA | Bipolar | rs11123818 | 0.2960 | 0.0838 | 0.000414453 |
| EA | Bipolar | rs111821073 | 0.2909 | 0.0835 | 0.000494119 |
| EA | Bipolar | rs11222609 | 0.2923 | 0.0835 | 0.000464591 |
| EA | Bipolar | rs112687095 | 0.2918 | 0.0835 | 0.000473734 |
| EA | Bipolar | rs113182709 | 0.2908 | 0.0835 | 0.000495565 |
| EA | Bipolar | rs113520408 | 0.2976 | 0.0833 | 0.000356027 |
| EA | Bipolar | rs113615161 | 0.2922 | 0.0835 | 0.000467106 |
| EA | Bipolar | rs1143770 | 0.2930 | 0.0835 | 0.000450763 |
| EA | Bipolar | rs11601122 | 0.2938 | 0.0836 | 0.000440913 |
| EA | Bipolar | rs11620355 | 0.2965 | 0.0833 | 0.000373313 |
| EA | Bipolar | rs11627087 | 0.2934 | 0.0835 | 0.00044066 |
| EA | Bipolar | rs11635092 | 0.2853 | 0.0833 | 0.00061172 |
| EA | Bipolar | rs11657342 | 0.2879 | 0.0834 | 0.000558475 |
| EA | Bipolar | rs11663602 | 0.2947 | 0.0835 | 0.000414868 |
| EA | Bipolar | rs11678980 | 0.3014 | 0.0834 | 0.000303145 |
| EA | Bipolar | rs11681861 | 0.2858 | 0.0831 | 0.000583164 |
| EA | Bipolar | rs11694904 | 0.2900 | 0.0835 | 0.000514119 |
| EA | Bipolar | rs11732657 | 0.2924 | 0.0835 | 0.000462795 |
| EA | Bipolar | rs117468730 | 0.2929 | 0.0835 | 0.000450852 |

| | | | | | |
|---|---|---|---|---|---|
| EA | Bipolar | rs11752914 | 0.2924 | 0.0835 | 0.000460807 |
| EA | Bipolar | rs11772580 | 0.2937 | 0.0835 | 0.000434976 |
| EA | Bipolar | rs117895796 | 0.2915 | 0.0835 | 0.000478598 |
| EA | Bipolar | rs11871429 | 0.2978 | 0.0834 | 0.000353381 |
| EA | Bipolar | rs12028010 | 0.3065 | 0.0828 | 0.000213494 |
| EA | Bipolar | rs12375949 | 0.2885 | 0.0836 | 0.000555903 |
| EA | Bipolar | rs12468040 | 0.3021 | 0.0832 | 0.000281348 |
| EA | Bipolar | rs12503522 | 0.2933 | 0.0835 | 0.000442352 |
| EA | Bipolar | rs12519073 | 0.2945 | 0.0835 | 0.000418079 |
| EA | Bipolar | rs12574281 | 0.2901 | 0.0835 | 0.000511398 |
| EA | Bipolar | rs12602286 | 0.2901 | 0.0835 | 0.000512368 |
| EA | Bipolar | rs12643771 | 0.2942 | 0.0836 | 0.000431557 |
| EA | Bipolar | rs12647336 | 0.2918 | 0.0835 | 0.000474004 |
| EA | Bipolar | rs12655753 | 0.2932 | 0.0835 | 0.000444615 |
| EA | Bipolar | rs12682775 | 0.2893 | 0.0834 | 0.000524298 |
| EA | Bipolar | rs12804787 | 0.2987 | 0.0830 | 0.000321084 |
| EA | Bipolar | rs1291818 | 0.2878 | 0.0834 | 0.000556045 |
| EA | Bipolar | rs12940014 | 0.2957 | 0.0834 | 0.000389845 |
| EA | Bipolar | rs13010566 | 0.2911 | 0.0835 | 0.000490241 |
| EA | Bipolar | rs13029509 | 0.2948 | 0.0835 | 0.000412292 |
| EA | Bipolar | rs13090388 | 0.2895 | 0.0842 | 0.000585859 |
| EA | Bipolar | rs13141210 | 0.2960 | 0.0835 | 0.000394906 |
| EA | Bipolar | rs13145650 | 0.2846 | 0.0831 | 0.000614527 |
| EA | Bipolar | rs1334297 | 0.3095 | 0.0834 | 0.0002062 |
| EA | Bipolar | rs13422673 | 0.2897 | 0.0835 | 0.000523807 |
| EA | Bipolar | rs13428598 | 0.3026 | 0.0833 | 0.000280862 |
| EA | Bipolar | rs1363862 | 0.2874 | 0.0833 | 0.000562661 |
| EA | Bipolar | rs1391438 | 0.2802 | 0.0832 | 0.000756188 |
| EA | Bipolar | rs1427298 | 0.2930 | 0.0835 | 0.000449408 |
| EA | Bipolar | rs1450782 | 0.2908 | 0.0835 | 0.000493154 |
| EA | Bipolar | rs152603 | 0.2950 | 0.0834 | 0.000406336 |
| EA | Bipolar | rs1558727 | 0.2875 | 0.0834 | 0.000563176 |
| EA | Bipolar | rs1566085 | 0.2976 | 0.0836 | 0.000370079 |
| EA | Bipolar | rs1569092 | 0.2955 | 0.0835 | 0.000402615 |
| EA | Bipolar | rs1582173 | 0.2904 | 0.0835 | 0.000505386 |
| EA | Bipolar | rs1584469 | 0.3008 | 0.0831 | 0.000294846 |
| EA | Bipolar | rs1595973 | 0.2901 | 0.0835 | 0.00050851 |
| EA | Bipolar | rs1618725 | 0.2909 | 0.0836 | 0.000503109 |
| EA | Bipolar | rs1620977 | 0.2791 | 0.0832 | 0.000798679 |
| EA | Bipolar | rs1671770 | 0.2860 | 0.0832 | 0.000587021 |
| EA | Bipolar | rs16846463 | 0.2930 | 0.0836 | 0.000457383 |
| EA | Bipolar | rs16854920 | 0.2972 | 0.0832 | 0.000355909 |
| EA | Bipolar | rs16995054 | 0.2869 | 0.0834 | 0.000578383 |
| EA | Bipolar | rs17048855 | 0.2961 | 0.0834 | 0.000385826 |
| EA | Bipolar | rs17126938 | 0.2794 | 0.0823 | 0.000682385 |
| EA | Bipolar | rs17425572 | 0.2809 | 0.0828 | 0.000695968 |
| EA | Bipolar | rs17489649 | 0.2926 | 0.0836 | 0.00046467 |
| EA | Bipolar | rs17551064 | 0.2896 | 0.0835 | 0.000521468 |
| EA | Bipolar | rs17563464 | 0.2914 | 0.0835 | 0.000485113 |
| EA | Bipolar | rs17565975 | 0.2872 | 0.0834 | 0.000570125 |
| EA | Bipolar | rs17598675 | 0.2878 | 0.0834 | 0.000561061 |
| EA | Bipolar | rs176218 | 0.2905 | 0.0836 | 0.000513248 |
| EA | Bipolar | rs1827540 | 0.2840 | 0.0830 | 0.000623403 |
| EA | Bipolar | rs1866823 | 0.2974 | 0.0833 | 0.000354519 |
| EA | Bipolar | rs192436652 | 0.2913 | 0.0835 | 0.00048849 |
| EA | Bipolar | rs1925576 | 0.2869 | 0.0833 | 0.00056918 |
| EA | Bipolar | rs1947114 | 0.2890 | 0.0834 | 0.000528607 |
| EA | Bipolar | rs1964927 | 0.2994 | 0.0834 | 0.000329534 |
| EA | Bipolar | rs2016392 | 0.2917 | 0.0835 | 0.0004772 |

| | | | | | |
|---|---|---|---|---|---|
| EA | Bipolar | rs2052285 | 0.2963 | 0.0834 | 0.00038059 |
| EA | Bipolar | rs2067854 | 0.2834 | 0.0831 | 0.00064965 |
| EA | Bipolar | rs2179152 | 0.2894 | 0.0836 | 0.000534324 |
| EA | Bipolar | rs2182505 | 0.2918 | 0.0835 | 0.000473709 |
| EA | Bipolar | rs225291 | 0.2978 | 0.0832 | 0.000344249 |
| EA | Bipolar | rs2256965 | 0.2866 | 0.0833 | 0.000581571 |
| EA | Bipolar | rs2283076 | 0.2966 | 0.0833 | 0.000370727 |
| EA | Bipolar | rs2287838 | 0.2915 | 0.0835 | 0.000484348 |
| EA | Bipolar | rs2302761 | 0.3004 | 0.0830 | 0.000298131 |
| EA | Bipolar | rs2347526 | 0.2964 | 0.0835 | 0.000386145 |
| EA | Bipolar | rs242093 | 0.2916 | 0.0835 | 0.000478857 |
| EA | Bipolar | rs2447535 | 0.2946 | 0.0835 | 0.000417971 |
| EA | Bipolar | rs2554835 | 0.2986 | 0.0831 | 0.00032609 |
| EA | Bipolar | rs2570497 | 0.2943 | 0.0835 | 0.00042587 |
| EA | Bipolar | rs2725370 | 0.2962 | 0.0835 | 0.000390916 |
| EA | Bipolar | rs277828 | 0.2963 | 0.0833 | 0.000376202 |
| EA | Bipolar | rs2819336 | 0.3043 | 0.0833 | 0.000257705 |
| EA | Bipolar | rs2820314 | 0.2886 | 0.0834 | 0.000539671 |
| EA | Bipolar | rs28513670 | 0.2951 | 0.0835 | 0.000406938 |
| EA | Bipolar | rs2885198 | 0.2955 | 0.0834 | 0.000395293 |
| EA | Bipolar | rs2901616 | 0.2883 | 0.0833 | 0.000542327 |
| EA | Bipolar | rs2905426 | 0.2997 | 0.0830 | 0.000305062 |
| EA | Bipolar | rs2971970 | 0.2868 | 0.0835 | 0.000589074 |
| EA | Bipolar | rs2998315 | 0.2946 | 0.0835 | 0.000421011 |
| EA | Bipolar | rs3013014 | 0.2884 | 0.0834 | 0.000541078 |
| EA | Bipolar | rs301800 | 0.2929 | 0.0835 | 0.00045518 |
| EA | Bipolar | rs3026996 | 0.2906 | 0.0836 | 0.000506394 |
| EA | Bipolar | rs31940 | 0.2883 | 0.0834 | 0.000547739 |
| EA | Bipolar | rs337637 | 0.2925 | 0.0835 | 0.000460323 |
| EA | Bipolar | rs34316 | 0.2911 | 0.0838 | 0.000515643 |
| EA | Bipolar | rs34394051 | 0.2974 | 0.0833 | 0.000354192 |
| EA | Bipolar | rs34485537 | 0.2930 | 0.0835 | 0.000449199 |
| EA | Bipolar | rs35039375 | 0.2950 | 0.0835 | 0.000410462 |
| EA | Bipolar | rs35309068 | 0.2880 | 0.0835 | 0.000561755 |
| EA | Bipolar | rs35316276 | 0.2918 | 0.0835 | 0.000473592 |
| EA | Bipolar | rs35417702 | 0.2913 | 0.0836 | 0.00049488 |
| EA | Bipolar | rs35475880 | 0.2918 | 0.0836 | 0.000479092 |
| EA | Bipolar | rs36083520 | 0.2825 | 0.0830 | 0.000667804 |
| EA | Bipolar | rs36119825 | 0.2958 | 0.0834 | 0.000390404 |
| EA | Bipolar | rs363096 | 0.3013 | 0.0832 | 0.000293423 |
| EA | Bipolar | rs3788556 | 0.2960 | 0.0834 | 0.000387908 |
| EA | Bipolar | rs3809634 | 0.2936 | 0.0835 | 0.000435815 |
| EA | Bipolar | rs3890802 | 0.2825 | 0.0827 | 0.000636133 |
| EA | Bipolar | rs3897821 | 0.2827 | 0.0832 | 0.000683577 |
| EA | Bipolar | rs4073894 | 0.2842 | 0.0832 | 0.000635542 |
| EA | Bipolar | rs4328757 | 0.2741 | 0.0809 | 0.000703679 |
| EA | Bipolar | rs4352658 | 0.2930 | 0.0835 | 0.000452248 |
| EA | Bipolar | rs4369924 | 0.2883 | 0.0834 | 0.000544699 |
| EA | Bipolar | rs4382592 | 0.2976 | 0.0835 | 0.00036674 |
| EA | Bipolar | rs4384309 | 0.2963 | 0.0834 | 0.000381381 |
| EA | Bipolar | rs4442732 | 0.2915 | 0.0835 | 0.00048149 |
| EA | Bipolar | rs4700393 | 0.3034 | 0.0836 | 0.000287214 |
| EA | Bipolar | rs4726070 | 0.2893 | 0.0835 | 0.000531949 |
| EA | Bipolar | rs4778058 | 0.2941 | 0.0835 | 0.000425296 |
| EA | Bipolar | rs4787457 | 0.3003 | 0.0835 | 0.000322993 |
| EA | Bipolar | rs4810227 | 0.2958 | 0.0835 | 0.000395078 |
| EA | Bipolar | rs4839155 | 0.2887 | 0.0834 | 0.000539458 |
| EA | Bipolar | rs4846724 | 0.2924 | 0.0835 | 0.000462366 |
| EA | Bipolar | rs4888746 | 0.2929 | 0.0835 | 0.000449747 |

| | | | | | |
|---|---|---|---|---|---|
| EA | Bipolar | rs4895650 | 0.2930 | 0.0835 | 0.000448246 |
| EA | Bipolar | rs4945424 | 0.2930 | 0.0835 | 0.000448589 |
| EA | Bipolar | rs4964046 | 0.2869 | 0.0833 | 0.000570468 |
| EA | Bipolar | rs4972400 | 0.2886 | 0.0834 | 0.000539761 |
| EA | Bipolar | rs4984541 | 0.2937 | 0.0835 | 0.000433762 |
| EA | Bipolar | rs532799 | 0.2944 | 0.0835 | 0.000419735 |
| EA | Bipolar | rs535307 | 0.2877 | 0.0833 | 0.000551959 |
| EA | Bipolar | rs56391344 | 0.2909 | 0.0836 | 0.000501696 |
| EA | Bipolar | rs575113 | 0.2845 | 0.0832 | 0.000626836 |
| EA | Bipolar | rs59123361 | 0.2995 | 0.0833 | 0.000320513 |
| EA | Bipolar | rs60904894 | 0.2934 | 0.0835 | 0.000441844 |
| EA | Bipolar | rs6122735 | 0.2980 | 0.0832 | 0.000344137 |
| EA | Bipolar | rs6123924 | 0.2876 | 0.0834 | 0.000561769 |
| EA | Bipolar | rs613872 | 0.2911 | 0.0836 | 0.000495814 |
| EA | Bipolar | rs62097985 | 0.3013 | 0.0831 | 0.000289063 |
| EA | Bipolar | rs62103084 | 0.2953 | 0.0834 | 0.000400411 |
| EA | Bipolar | rs62157915 | 0.2928 | 0.0835 | 0.000453588 |
| EA | Bipolar | rs62183776 | 0.2946 | 0.0835 | 0.000415321 |
| EA | Bipolar | rs62184480 | 0.2952 | 0.0836 | 0.000411013 |
| EA | Bipolar | rs622169 | 0.2915 | 0.0835 | 0.000479677 |
| EA | Bipolar | rs62439690 | 0.2926 | 0.0835 | 0.000457116 |
| EA | Bipolar | rs62444881 | 0.2789 | 0.0829 | 0.000770248 |
| EA | Bipolar | rs6493265 | 0.2974 | 0.0835 | 0.000366512 |
| EA | Bipolar | rs6513959 | 0.2762 | 0.0816 | 0.000716157 |
| EA | Bipolar | rs6557171 | 0.3002 | 0.0834 | 0.000317516 |
| EA | Bipolar | rs66568921 | 0.2965 | 0.0836 | 0.000388708 |
| EA | Bipolar | rs6731373 | 0.2869 | 0.0834 | 0.000578699 |
| EA | Bipolar | rs67885444 | 0.2931 | 0.0835 | 0.00044658 |
| EA | Bipolar | rs67890737 | 0.2892 | 0.0835 | 0.000529229 |
| EA | Bipolar | rs6803651 | 0.2915 | 0.0835 | 0.000482915 |
| EA | Bipolar | rs6805241 | 0.2889 | 0.0835 | 0.000538977 |
| EA | Bipolar | rs6938002 | 0.2917 | 0.0835 | 0.000476976 |
| EA | Bipolar | rs6959891 | 0.2886 | 0.0834 | 0.000539908 |
| EA | Bipolar | rs7012546 | 0.2888 | 0.0834 | 0.000534246 |
| EA | Bipolar | rs702606 | 0.2905 | 0.0835 | 0.000499827 |
| EA | Bipolar | rs7029718 | 0.2743 | 0.0836 | 0.001029511 |
| EA | Bipolar | rs7031698 | 0.2980 | 0.0832 | 0.000342004 |
| EA | Bipolar | rs710629 | 0.2895 | 0.0834 | 0.000521582 |
| EA | Bipolar | rs71646142 | 0.2940 | 0.0835 | 0.000427353 |
| EA | Bipolar | rs7233920 | 0.2906 | 0.0835 | 0.000501308 |
| EA | Bipolar | rs72486027 | 0.2877 | 0.0833 | 0.000552674 |
| EA | Bipolar | rs7257460 | 0.2947 | 0.0835 | 0.000414147 |
| EA | Bipolar | rs72807818 | 0.2991 | 0.0833 | 0.000332173 |
| EA | Bipolar | rs72828517 | 0.2960 | 0.0835 | 0.000394845 |
| EA | Bipolar | rs72840994 | 0.2896 | 0.0834 | 0.000518067 |
| EA | Bipolar | rs730384 | 0.2949 | 0.0834 | 0.000409605 |
| EA | Bipolar | rs73301698 | 0.2916 | 0.0835 | 0.000480301 |
| EA | Bipolar | rs7332724 | 0.2985 | 0.0832 | 0.000334122 |
| EA | Bipolar | rs73344830 | 0.2881 | 0.0837 | 0.000574785 |
| EA | Bipolar | rs736282 | 0.2944 | 0.0835 | 0.000420556 |
| EA | Bipolar | rs73874335 | 0.2905 | 0.0835 | 0.000500623 |
| EA | Bipolar | rs743316 | 0.2968 | 0.0833 | 0.000365915 |
| EA | Bipolar | rs74643044 | 0.2925 | 0.0835 | 0.000459871 |
| EA | Bipolar | rs74701752 | 0.2963 | 0.0833 | 0.000376635 |
| EA | Bipolar | rs74998289 | 0.3001 | 0.0834 | 0.000319796 |
| EA | Bipolar | rs7543462 | 0.2911 | 0.0835 | 0.000488486 |
| EA | Bipolar | rs7594904 | 0.2961 | 0.0833 | 0.000380764 |
| EA | Bipolar | rs7603132 | 0.2999 | 0.0830 | 0.000302775 |
| EA | Bipolar | rs76076331 | 0.2976 | 0.0834 | 0.000360813 |

| | | | | | |
|---|---|---|---|---|---|
| EA | Bipolar | rs7650602 | 0.2888 | 0.0834 | 0.0005326 |
| EA | Bipolar | rs76608582 | 0.2911 | 0.0835 | 0.00048888 |
| EA | Bipolar | rs77025239 | 0.2888 | 0.0834 | 0.000536136 |
| EA | Bipolar | rs77128898 | 0.2974 | 0.0833 | 0.000353212 |
| EA | Bipolar | rs77702622 | 0.2920 | 0.0835 | 0.000473247 |
| EA | Bipolar | rs7773815 | 0.2945 | 0.0834 | 0.000415544 |
| EA | Bipolar | rs77835879 | 0.2885 | 0.0833 | 0.000536789 |
| EA | Bipolar | rs7796203 | 0.2865 | 0.0833 | 0.000580989 |
| EA | Bipolar | rs7803932 | 0.2967 | 0.0834 | 0.000371088 |
| EA | Bipolar | rs7808399 | 0.2910 | 0.0835 | 0.000492191 |
| EA | Bipolar | rs7863447 | 0.2975 | 0.0834 | 0.000362015 |
| EA | Bipolar | rs7864396 | 0.2993 | 0.0830 | 0.000310927 |
| EA | Bipolar | rs78721320 | 0.2939 | 0.0835 | 0.00042968 |
| EA | Bipolar | rs790647 | 0.2942 | 0.0835 | 0.000428774 |
| EA | Bipolar | rs7924036 | 0.2969 | 0.0836 | 0.000380206 |
| EA | Bipolar | rs79265434 | 0.2738 | 0.0824 | 0.00088995 |
| EA | Bipolar | rs79269403 | 0.2965 | 0.0834 | 0.000380429 |
| EA | Bipolar | rs795230 | 0.2910 | 0.0835 | 0.000489559 |
| EA | Bipolar | rs79523955 | 0.2966 | 0.0834 | 0.000373739 |
| EA | Bipolar | rs7977614 | 0.2937 | 0.0835 | 0.000436864 |
| EA | Bipolar | rs7993663 | 0.2992 | 0.0832 | 0.000322921 |
| EA | Bipolar | rs8008382 | 0.2896 | 0.0835 | 0.000521731 |
| EA | Bipolar | rs80171383 | 0.2984 | 0.0832 | 0.000332342 |
| EA | Bipolar | rs8020034 | 0.2957 | 0.0835 | 0.00040021 |
| EA | Bipolar | rs818415 | 0.2886 | 0.0834 | 0.000538488 |
| EA | Bipolar | rs837080 | 0.2958 | 0.0834 | 0.00039264 |
| EA | Bipolar | rs892612 | 0.2901 | 0.0835 | 0.000511398 |
| EA | Bipolar | rs894067 | 0.2897 | 0.0835 | 0.000517471 |
| EA | Bipolar | rs9289300 | 0.2942 | 0.0835 | 0.000426382 |
| EA | Bipolar | rs9320493 | 0.2946 | 0.0834 | 0.000413842 |
| EA | Bipolar | rs9342482 | 0.2916 | 0.0835 | 0.00047971 |
| EA | Bipolar | rs9349956 | 0.2822 | 0.0832 | 0.000691965 |
| EA | Bipolar | rs9372625 | 0.2704 | 0.0833 | 0.001164577 |
| EA | Bipolar | rs9384679 | 0.2953 | 0.0834 | 0.00039829 |
| EA | Bipolar | rs9386319 | 0.2943 | 0.0835 | 0.000420527 |
| EA | Bipolar | rs9436866 | 0.2918 | 0.0835 | 0.00047739 |
| EA | Bipolar | rs9503598 | 0.2953 | 0.0834 | 0.000402086 |
| EA | Bipolar | rs9556958 | 0.2906 | 0.0835 | 0.000500849 |
| EA | Bipolar | rs9616906 | 0.2809 | 0.0832 | 0.000729891 |
| EA | Bipolar | rs9679654 | 0.2898 | 0.0835 | 0.000515862 |
| EA | Bipolar | rs9704097 | 0.2927 | 0.0835 | 0.000456361 |
| EA | Bipolar | rs9882532 | 0.2990 | 0.0832 | 0.000328768 |
| EA | Bipolar | rs9914918 | 0.2914 | 0.0835 | 0.000484423 |
| EA | Bipolar | rs9933256 | 0.2903 | 0.0835 | 0.000505477 |
| EA | Bipolar | rs9936270 | 0.2850 | 0.0832 | 0.000616349 |
| EA | Bipolar | rs9964724 | 0.2944 | 0.0838 | 0.000439521 |
| EA | Bipolar | rs9995567 | 0.2930 | 0.0835 | 0.000448081 |
| EA | Bipolar | All | 0.2921 | 0.0832 | 0.000447975 |
| EA | SZspecific | rs10073890 | -0.1460 | 0.1514 | 0.334771721 |
| EA | SZspecific | rs1008078 | -0.1369 | 0.1517 | 0.36663689 |
| EA | SZspecific | rs10189857 | -0.1610 | 0.1512 | 0.287136046 |
| EA | SZspecific | rs10205801 | -0.1632 | 0.1497 | 0.275650043 |
| EA | SZspecific | rs10215082 | -0.1281 | 0.1509 | 0.395670488 |
| EA | SZspecific | rs10240905 | -0.1386 | 0.1514 | 0.359852154 |
| EA | SZspecific | rs10456918 | -0.1523 | 0.1511 | 0.313660884 |
| EA | SZspecific | rs10460095 | -0.1339 | 0.1510 | 0.375465383 |
| EA | SZspecific | rs1051474 | -0.1303 | 0.1509 | 0.387671838 |
| EA | SZspecific | rs10760023 | -0.1456 | 0.1514 | 0.336004425 |
| EA | SZspecific | rs10765775 | -0.1528 | 0.1514 | 0.312878299 |

| | | | | | |
|---|---|---|---|---|---|
| EA | SZspecific | rs10772644 | -0.1435 | 0.1514 | 0.343224373 |
| EA | SZspecific | rs10773002 | -0.1823 | 0.1500 | 0.224178798 |
| EA | SZspecific | rs10856785 | -0.1430 | 0.1514 | 0.34477453 |
| EA | SZspecific | rs10862376 | -0.1429 | 0.1515 | 0.345488993 |
| EA | SZspecific | rs10875121 | -0.1821 | 0.1476 | 0.217485603 |
| EA | SZspecific | rs10887801 | -0.1468 | 0.1514 | 0.332053204 |
| EA | SZspecific | rs10940921 | -0.1348 | 0.1511 | 0.372392513 |
| EA | SZspecific | rs10963297 | -0.1459 | 0.1517 | 0.336209077 |
| EA | SZspecific | rs10994777 | -0.1455 | 0.1514 | 0.336532975 |
| EA | SZspecific | rs11023749 | -0.1419 | 0.1514 | 0.348670413 |
| EA | SZspecific | rs1105307 | -0.1309 | 0.1507 | 0.38527351 |
| EA | SZspecific | rs1106090 | -0.1591 | 0.1506 | 0.290633522 |
| EA | SZspecific | rs11081529 | -0.1418 | 0.1515 | 0.349253347 |
| EA | SZspecific | rs11123818 | -0.1447 | 0.1519 | 0.341058435 |
| EA | SZspecific | rs111821073 | -0.1346 | 0.1510 | 0.372888021 |
| EA | SZspecific | rs11222609 | -0.1414 | 0.1514 | 0.350409962 |
| EA | SZspecific | rs112687095 | -0.1449 | 0.1514 | 0.338440405 |
| EA | SZspecific | rs112806496 | -0.1409 | 0.1514 | 0.352110574 |
| EA | SZspecific | rs113182709 | -0.1372 | 0.1512 | 0.364239441 |
| EA | SZspecific | rs113520408 | -0.1369 | 0.1513 | 0.365621795 |
| EA | SZspecific | rs113615161 | -0.1497 | 0.1512 | 0.322245818 |
| EA | SZspecific | rs1143770 | -0.1402 | 0.1514 | 0.354527331 |
| EA | SZspecific | rs115000530 | -0.1493 | 0.1514 | 0.324191616 |
| EA | SZspecific | rs11601122 | -0.1443 | 0.1516 | 0.341284724 |
| EA | SZspecific | rs11620355 | -0.1402 | 0.1513 | 0.354328364 |
| EA | SZspecific | rs11627087 | -0.1403 | 0.1513 | 0.353922065 |
| EA | SZspecific | rs11635092 | -0.1347 | 0.1512 | 0.37301785 |
| EA | SZspecific | rs11657342 | -0.1295 | 0.1508 | 0.390429566 |
| EA | SZspecific | rs11663602 | -0.1212 | 0.1495 | 0.417540827 |
| EA | SZspecific | rs11678980 | -0.1312 | 0.1515 | 0.386417376 |
| EA | SZspecific | rs11681861 | -0.1432 | 0.1514 | 0.344048405 |
| EA | SZspecific | rs11694904 | -0.1528 | 0.1511 | 0.311978471 |
| EA | SZspecific | rs11732657 | -0.1468 | 0.1514 | 0.332302725 |
| EA | SZspecific | rs117468730 | -0.1434 | 0.1514 | 0.343605043 |
| EA | SZspecific | rs11752914 | -0.1543 | 0.1507 | 0.305894556 |
| EA | SZspecific | rs11772580 | -0.1490 | 0.1512 | 0.324603138 |
| EA | SZspecific | rs117799466 | -0.1269 | 0.1503 | 0.398404319 |
| EA | SZspecific | rs11871429 | -0.1528 | 0.1512 | 0.312134747 |
| EA | SZspecific | rs12028010 | -0.1529 | 0.1514 | 0.312389154 |
| EA | SZspecific | rs12332731 | -0.1434 | 0.1514 | 0.343508529 |
| EA | SZspecific | rs12375949 | -0.1376 | 0.1515 | 0.363768778 |
| EA | SZspecific | rs12468040 | -0.1460 | 0.1515 | 0.335366412 |
| EA | SZspecific | rs12503522 | -0.1510 | 0.1511 | 0.317880465 |
| EA | SZspecific | rs12519073 | -0.1461 | 0.1514 | 0.334390631 |
| EA | SZspecific | rs12574281 | -0.1426 | 0.1514 | 0.346179194 |
| EA | SZspecific | rs12602286 | -0.1627 | 0.1501 | 0.278383516 |
| EA | SZspecific | rs12643771 | -0.1614 | 0.1507 | 0.284342053 |
| EA | SZspecific | rs12647336 | -0.1439 | 0.1514 | 0.341704948 |
| EA | SZspecific | rs12655753 | -0.1412 | 0.1514 | 0.350810899 |
| EA | SZspecific | rs12682775 | -0.1392 | 0.1513 | 0.357722074 |
| EA | SZspecific | rs12804787 | -0.1450 | 0.1513 | 0.337912954 |
| EA | SZspecific | rs1291818 | -0.1288 | 0.1506 | 0.39215729 |
| EA | SZspecific | rs12940014 | -0.1374 | 0.1512 | 0.363516846 |
| EA | SZspecific | rs13010566 | -0.1424 | 0.1514 | 0.347033943 |
| EA | SZspecific | rs13029509 | -0.1341 | 0.1510 | 0.374539345 |
| EA | SZspecific | rs13090388 | -0.1580 | 0.1523 | 0.299640308 |
| EA | SZspecific | rs13141210 | -0.1385 | 0.1515 | 0.3605803 |
| EA | SZspecific | rs13145650 | -0.1465 | 0.1514 | 0.333250828 |
| EA | SZspecific | rs1334297 | -0.1569 | 0.1519 | 0.301722061 |

| | | | | | |
|---|---|---|---|---|---|
| EA | SZspecific | rs13422673 | -0.1392 | 0.1514 | 0.357961742 |
| EA | SZspecific | rs13428598 | -0.1447 | 0.1517 | 0.340001516 |
| EA | SZspecific | rs1363862 | -0.1470 | 0.1513 | 0.331266421 |
| EA | SZspecific | rs1391438 | -0.1412 | 0.1517 | 0.351901479 |
| EA | SZspecific | rs1427298 | -0.1554 | 0.1507 | 0.302412341 |
| EA | SZspecific | rs1450782 | -0.1384 | 0.1512 | 0.360100479 |
| EA | SZspecific | rs152603 | -0.1576 | 0.1504 | 0.294558896 |
| EA | SZspecific | rs1558727 | -0.1329 | 0.1510 | 0.378562906 |
| EA | SZspecific | rs1566085 | -0.1594 | 0.1512 | 0.291773953 |
| EA | SZspecific | rs1569092 | -0.1404 | 0.1515 | 0.353879891 |
| EA | SZspecific | rs1582173 | -0.1504 | 0.1512 | 0.319703244 |
| EA | SZspecific | rs1584469 | -0.1501 | 0.1513 | 0.321144753 |
| EA | SZspecific | rs1592757 | -0.1427 | 0.1514 | 0.345772094 |
| EA | SZspecific | rs1595973 | -0.1428 | 0.1514 | 0.345380758 |
| EA | SZspecific | rs1618725 | -0.1394 | 0.1516 | 0.357755922 |
| EA | SZspecific | rs1620977 | -0.1381 | 0.1517 | 0.362854898 |
| EA | SZspecific | rs1671770 | -0.1317 | 0.1508 | 0.382559011 |
| EA | SZspecific | rs16846463 | -0.1412 | 0.1515 | 0.351564393 |
| EA | SZspecific | rs16854920 | -0.1574 | 0.1503 | 0.294862294 |
| EA | SZspecific | rs1689510 | -0.1555 | 0.1515 | 0.304625193 |
| EA | SZspecific | rs16995054 | -0.1351 | 0.1512 | 0.371619137 |
| EA | SZspecific | rs17048855 | -0.1358 | 0.1512 | 0.369301751 |
| EA | SZspecific | rs17126938 | -0.1323 | 0.1509 | 0.38081424 |
| EA | SZspecific | rs17425572 | -0.1320 | 0.1511 | 0.382096962 |
| EA | SZspecific | rs17489649 | -0.1513 | 0.1513 | 0.317548267 |
| EA | SZspecific | rs175325 | -0.1321 | 0.1510 | 0.381712256 |
| EA | SZspecific | rs17551064 | -0.1464 | 0.1514 | 0.333387663 |
| EA | SZspecific | rs17563464 | -0.1438 | 0.1514 | 0.342311812 |
| EA | SZspecific | rs17565975 | -0.1358 | 0.1512 | 0.369353569 |
| EA | SZspecific | rs17598675 | -0.1407 | 0.1514 | 0.353032146 |
| EA | SZspecific | rs176218 | -0.1292 | 0.1512 | 0.392694813 |
| EA | SZspecific | rs1827540 | -0.1427 | 0.1514 | 0.346133933 |
| EA | SZspecific | rs1866823 | -0.1396 | 0.1513 | 0.356166383 |
| EA | SZspecific | rs1882273 | -0.1378 | 0.1513 | 0.362499789 |
| EA | SZspecific | rs192436652 | -0.1523 | 0.1511 | 0.31366077 |
| EA | SZspecific | rs1925576 | -0.1423 | 0.1514 | 0.347220415 |
| EA | SZspecific | rs1947114 | -0.1415 | 0.1514 | 0.349778196 |
| EA | SZspecific | rs1964927 | -0.1530 | 0.1513 | 0.311974891 |
| EA | SZspecific | rs2016392 | -0.1356 | 0.1511 | 0.369610857 |
| EA | SZspecific | rs2052285 | -0.1406 | 0.1514 | 0.353092646 |
| EA | SZspecific | rs2067854 | -0.1598 | 0.1506 | 0.288539438 |
| EA | SZspecific | rs2179152 | -0.1367 | 0.1515 | 0.366896282 |
| EA | SZspecific | rs2182505 | -0.1433 | 0.1514 | 0.343968974 |
| EA | SZspecific | rs225291 | -0.1537 | 0.1508 | 0.30825307 |
| EA | SZspecific | rs2256965 | -0.1360 | 0.1512 | 0.36844049 |
| EA | SZspecific | rs2283076 | -0.1400 | 0.1513 | 0.354741543 |
| EA | SZspecific | rs2287838 | -0.1336 | 0.1511 | 0.376612407 |
| EA | SZspecific | rs2302761 | -0.1536 | 0.1510 | 0.309251959 |
| EA | SZspecific | rs2347526 | -0.1552 | 0.1512 | 0.304486406 |
| EA | SZspecific | rs242093 | -0.1405 | 0.1514 | 0.35320602 |
| EA | SZspecific | rs2447535 | -0.1376 | 0.1513 | 0.362913786 |
| EA | SZspecific | rs2554835 | -0.1440 | 0.1514 | 0.341464574 |
| EA | SZspecific | rs2570497 | -0.1490 | 0.1514 | 0.325084539 |
| EA | SZspecific | rs2725370 | -0.1450 | 0.1516 | 0.338679398 |
| EA | SZspecific | rs277828 | -0.1369 | 0.1511 | 0.364904624 |
| EA | SZspecific | rs2819336 | -0.1302 | 0.1515 | 0.390170845 |
| EA | SZspecific | rs2820314 | -0.1330 | 0.1509 | 0.378313569 |
| EA | SZspecific | rs28373063 | -0.1492 | 0.1512 | 0.323800353 |
| EA | SZspecific | rs28513670 | -0.1547 | 0.1510 | 0.305484177 |

| | | | | | |
|---|---|---|---|---|---|
| EA | SZspecific | rs2885198 | -0.1489 | 0.1513 | 0.324743793 |
| EA | SZspecific | rs2901616 | -0.1553 | 0.1506 | 0.302454589 |
| EA | SZspecific | rs2905426 | -0.1226 | 0.1493 | 0.411836076 |
| EA | SZspecific | rs2923431 | -0.1430 | 0.1514 | 0.344951195 |
| EA | SZspecific | rs2971970 | -0.1319 | 0.1512 | 0.383069457 |
| EA | SZspecific | rs2998315 | -0.1474 | 0.1514 | 0.330331696 |
| EA | SZspecific | rs3013014 | -0.1447 | 0.1514 | 0.339065606 |
| EA | SZspecific | rs301800 | -0.1326 | 0.1511 | 0.380210283 |
| EA | SZspecific | rs3026996 | -0.1525 | 0.1513 | 0.31343086 |
| EA | SZspecific | rs31940 | -0.1357 | 0.1512 | 0.369385192 |
| EA | SZspecific | rs337637 | -0.1429 | 0.1514 | 0.345136552 |
| EA | SZspecific | rs34316 | -0.1230 | 0.1514 | 0.41666957 |
| EA | SZspecific | rs34394051 | -0.1583 | 0.1504 | 0.292515432 |
| EA | SZspecific | rs34485537 | -0.1397 | 0.1513 | 0.355967091 |
| EA | SZspecific | rs34853711 | -0.1519 | 0.1513 | 0.315539638 |
| EA | SZspecific | rs35039375 | -0.1452 | 0.1514 | 0.337660877 |
| EA | SZspecific | rs35309068 | -0.1457 | 0.1515 | 0.33619635 |
| EA | SZspecific | rs35316276 | -0.1385 | 0.1513 | 0.359775536 |
| EA | SZspecific | rs35417702 | -0.1699 | 0.1500 | 0.257386537 |
| EA | SZspecific | rs35475880 | -0.1508 | 0.1513 | 0.318815528 |
| EA | SZspecific | rs35532491 | -0.1482 | 0.1514 | 0.327556468 |
| EA | SZspecific | rs36083520 | -0.1417 | 0.1515 | 0.349640086 |
| EA | SZspecific | rs36119825 | -0.1529 | 0.1510 | 0.311243308 |
| EA | SZspecific | rs363096 | -0.1431 | 0.1515 | 0.344981739 |
| EA | SZspecific | rs3747631 | -0.1339 | 0.1517 | 0.377378078 |
| EA | SZspecific | rs3788556 | -0.1235 | 0.1500 | 0.410036125 |
| EA | SZspecific | rs3800546 | -0.1466 | 0.1514 | 0.33271906 |
| EA | SZspecific | rs3809634 | -0.1434 | 0.1514 | 0.343412285 |
| EA | SZspecific | rs3890802 | -0.1525 | 0.1510 | 0.312455799 |
| EA | SZspecific | rs3897821 | -0.1563 | 0.1512 | 0.301138966 |
| EA | SZspecific | rs406413 | -0.1406 | 0.1515 | 0.353678307 |
| EA | SZspecific | rs4073894 | -0.1266 | 0.1506 | 0.400446208 |
| EA | SZspecific | rs4328757 | -0.1364 | 0.1512 | 0.36707915 |
| EA | SZspecific | rs4352658 | -0.1459 | 0.1514 | 0.335218092 |
| EA | SZspecific | rs4369924 | -0.1460 | 0.1514 | 0.334682395 |
| EA | SZspecific | rs4382592 | -0.1478 | 0.1516 | 0.32950329 |
| EA | SZspecific | rs4384309 | -0.1481 | 0.1513 | 0.327865236 |
| EA | SZspecific | rs4442732 | -0.1328 | 0.1509 | 0.378898742 |
| EA | SZspecific | rs4667029 | -0.1473 | 0.1513 | 0.330367941 |
| EA | SZspecific | rs4700393 | -0.1178 | 0.1513 | 0.436178147 |
| EA | SZspecific | rs4726070 | -0.1397 | 0.1514 | 0.356281913 |
| EA | SZspecific | rs4733264 | -0.1512 | 0.1510 | 0.316817737 |
| EA | SZspecific | rs4757957 | -0.1469 | 0.1515 | 0.332080343 |
| EA | SZspecific | rs4766424 | -0.1360 | 0.1511 | 0.367903799 |
| EA | SZspecific | rs4778058 | -0.1473 | 0.1513 | 0.330251472 |
| EA | SZspecific | rs4787457 | -0.1398 | 0.1517 | 0.356763401 |
| EA | SZspecific | rs4810227 | -0.1424 | 0.1515 | 0.347319706 |
| EA | SZspecific | rs4839155 | -0.1417 | 0.1514 | 0.349165432 |
| EA | SZspecific | rs4846724 | -0.1489 | 0.1513 | 0.324907441 |
| EA | SZspecific | rs4870482 | -0.1435 | 0.1514 | 0.3432751 |
| EA | SZspecific | rs4888746 | -0.1402 | 0.1513 | 0.354204471 |
| EA | SZspecific | rs4895650 | -0.1363 | 0.1511 | 0.366962269 |
| EA | SZspecific | rs4945424 | -0.1504 | 0.1511 | 0.319731179 |
| EA | SZspecific | rs4964046 | -0.1434 | 0.1514 | 0.34356403 |
| EA | SZspecific | rs4972400 | -0.1474 | 0.1513 | 0.330233508 |
| EA | SZspecific | rs4984541 | -0.1468 | 0.1513 | 0.33202304 |
| EA | SZspecific | rs532799 | -0.1530 | 0.1509 | 0.310878848 |
| EA | SZspecific | rs535307 | -0.1430 | 0.1514 | 0.344908011 |
| EA | SZspecific | rs55736314 | -0.1652 | 0.1504 | 0.272044397 |

| | | | | | |
|---|---|---|---|---|---|
| EA | SZspecific | rs55771711 | -0.1384 | 0.1515 | 0.360863496 |
| EA | SZspecific | rs56391344 | -0.1398 | 0.1515 | 0.35624822 |
| EA | SZspecific | rs575113 | -0.1404 | 0.1514 | 0.353874698 |
| EA | SZspecific | rs59123361 | -0.1485 | 0.1514 | 0.326674963 |
| EA | SZspecific | rs59480703 | -0.1500 | 0.1512 | 0.321032302 |
| EA | SZspecific | rs60904894 | -0.1434 | 0.1514 | 0.343437553 |
| EA | SZspecific | rs6122735 | -0.1554 | 0.1508 | 0.302812669 |
| EA | SZspecific | rs6123924 | -0.1351 | 0.1512 | 0.371326045 |
| EA | SZspecific | rs613872 | -0.1526 | 0.1513 | 0.313165357 |
| EA | SZspecific | rs62097985 | -0.1325 | 0.1512 | 0.380860095 |
| EA | SZspecific | rs62103084 | -0.1473 | 0.1513 | 0.330273135 |
| EA | SZspecific | rs62157915 | -0.1449 | 0.1514 | 0.338527584 |
| EA | SZspecific | rs62183776 | -0.1514 | 0.1511 | 0.316373952 |
| EA | SZspecific | rs62184480 | -0.1449 | 0.1515 | 0.338955703 |
| EA | SZspecific | rs622169 | -0.1528 | 0.1508 | 0.311042131 |
| EA | SZspecific | rs62439690 | -0.1451 | 0.1514 | 0.337866938 |
| EA | SZspecific | rs62444881 | -0.1543 | 0.1513 | 0.307935052 |
| EA | SZspecific | rs6493265 | -0.1381 | 0.1515 | 0.361794412 |
| EA | SZspecific | rs6513959 | -0.1434 | 0.1514 | 0.343513957 |
| EA | SZspecific | rs6557171 | -0.1510 | 0.1515 | 0.318862514 |
| EA | SZspecific | rs663234 | -0.1375 | 0.1512 | 0.363295151 |
| EA | SZspecific | rs66568921 | -0.1517 | 0.1515 | 0.316424012 |
| EA | SZspecific | rs6731373 | -0.1475 | 0.1514 | 0.330022661 |
| EA | SZspecific | rs6731967 | -0.1531 | 0.1510 | 0.310472332 |
| EA | SZspecific | rs67885444 | -0.1507 | 0.1512 | 0.318940469 |
| EA | SZspecific | rs67890737 | -0.1576 | 0.1506 | 0.295331034 |
| EA | SZspecific | rs6803651 | -0.1378 | 0.1513 | 0.362375764 |
| EA | SZspecific | rs6805241 | -0.1426 | 0.1515 | 0.346358286 |
| EA | SZspecific | rs6938002 | -0.1472 | 0.1513 | 0.330620227 |
| EA | SZspecific | rs6959891 | -0.1374 | 0.1512 | 0.363631788 |
| EA | SZspecific | rs7012546 | -0.1369 | 0.1512 | 0.365338111 |
| EA | SZspecific | rs7016302 | -0.1425 | 0.1514 | 0.346490832 |
| EA | SZspecific | rs702606 | -0.1381 | 0.1512 | 0.361120612 |
| EA | SZspecific | rs7029718 | -0.1048 | 0.1510 | 0.487723533 |
| EA | SZspecific | rs7031698 | -0.1468 | 0.1513 | 0.331993488 |
| EA | SZspecific | rs710629 | -0.1346 | 0.1510 | 0.372929098 |
| EA | SZspecific | rs71646142 | -0.1392 | 0.1513 | 0.35761519 |
| EA | SZspecific | rs7233920 | -0.1382 | 0.1513 | 0.361174606 |
| EA | SZspecific | rs72486027 | -0.1484 | 0.1513 | 0.326677394 |
| EA | SZspecific | rs7257460 | -0.1467 | 0.1514 | 0.332305262 |
| EA | SZspecific | rs7278859 | -0.1405 | 0.1513 | 0.353226109 |
| EA | SZspecific | rs72807818 | -0.1587 | 0.1509 | 0.292854615 |
| EA | SZspecific | rs72828517 | -0.1318 | 0.1512 | 0.383336087 |
| EA | SZspecific | rs72840994 | -0.1430 | 0.1514 | 0.344997528 |
| EA | SZspecific | rs730384 | -0.1367 | 0.1512 | 0.365965923 |
| EA | SZspecific | rs7315713 | -0.1361 | 0.1511 | 0.367765476 |
| EA | SZspecific | rs73301698 | -0.1406 | 0.1514 | 0.352920345 |
| EA | SZspecific | rs7332724 | -0.1387 | 0.1513 | 0.359213392 |
| EA | SZspecific | rs73344830 | -0.1442 | 0.1517 | 0.341830298 |
| EA | SZspecific | rs736282 | -0.1478 | 0.1513 | 0.328684174 |
| EA | SZspecific | rs73874335 | -0.1366 | 0.1511 | 0.366195878 |
| EA | SZspecific | rs743316 | -0.1410 | 0.1514 | 0.351389567 |
| EA | SZspecific | rs74643044 | -0.1426 | 0.1514 | 0.3461045 |
| EA | SZspecific | rs74701752 | -0.1405 | 0.1513 | 0.353211275 |
| EA | SZspecific | rs74998289 | -0.1798 | 0.1486 | 0.226482164 |
| EA | SZspecific | rs7543462 | -0.1380 | 0.1512 | 0.361470301 |
| EA | SZspecific | rs7594904 | -0.1459 | 0.1513 | 0.335136315 |
| EA | SZspecific | rs7603132 | -0.1440 | 0.1514 | 0.341424218 |
| EA | SZspecific | rs76076331 | -0.1315 | 0.1511 | 0.383976713 |

| | | | | | |
|---|---|---|---|---|---|
| EA | SZspecific | rs7650602 | -0.1416 | 0.1514 | 0.349529102 |
| EA | SZspecific | rs76608582 | -0.1444 | 0.1514 | 0.339994861 |
| EA | SZspecific | rs77025239 | -0.1396 | 0.1513 | 0.356409579 |
| EA | SZspecific | rs77128898 | -0.1469 | 0.1513 | 0.331683229 |
| EA | SZspecific | rs77702622 | -0.1380 | 0.1513 | 0.361734221 |
| EA | SZspecific | rs77719387 | -0.1359 | 0.1511 | 0.368421243 |
| EA | SZspecific | rs7773815 | -0.1477 | 0.1513 | 0.329064745 |
| EA | SZspecific | rs77835879 | -0.1441 | 0.1514 | 0.340982232 |
| EA | SZspecific | rs7796203 | -0.1402 | 0.1514 | 0.354197028 |
| EA | SZspecific | rs7803932 | -0.1360 | 0.1512 | 0.368424872 |
| EA | SZspecific | rs7808399 | -0.1481 | 0.1513 | 0.327673883 |
| EA | SZspecific | rs7833201 | -0.1427 | 0.1514 | 0.345816808 |
| EA | SZspecific | rs7863447 | -0.1484 | 0.1514 | 0.326976929 |
| EA | SZspecific | rs7864396 | -0.1375 | 0.1512 | 0.362994017 |
| EA | SZspecific | rs78721320 | -0.1438 | 0.1514 | 0.342198095 |
| EA | SZspecific | rs790647 | -0.1407 | 0.1515 | 0.352943528 |
| EA | SZspecific | rs7924036 | -0.1574 | 0.1512 | 0.29780964 |
| EA | SZspecific | rs79265434 | -0.1567 | 0.1512 | 0.300308365 |
| EA | SZspecific | rs79269403 | -0.1449 | 0.1515 | 0.338697527 |
| EA | SZspecific | rs7928622 | -0.1414 | 0.1514 | 0.350258042 |
| EA | SZspecific | rs795230 | -0.1382 | 0.1512 | 0.360816411 |
| EA | SZspecific | rs79523955 | -0.1404 | 0.1514 | 0.353630062 |
| EA | SZspecific | rs7977614 | -0.1324 | 0.1510 | 0.380510129 |
| EA | SZspecific | rs7993663 | -0.1425 | 0.1514 | 0.346617265 |
| EA | SZspecific | rs8008382 | -0.1508 | 0.1512 | 0.318555003 |
| EA | SZspecific | rs80171383 | -0.1431 | 0.1514 | 0.344454921 |
| EA | SZspecific | rs8020034 | -0.1386 | 0.1515 | 0.360399379 |
| EA | SZspecific | rs818415 | -0.1481 | 0.1513 | 0.327680948 |
| EA | SZspecific | rs837080 | -0.1271 | 0.1504 | 0.398113238 |
| EA | SZspecific | rs892612 | -0.1496 | 0.1512 | 0.322698887 |
| EA | SZspecific | rs894067 | -0.1473 | 0.1513 | 0.330290465 |
| EA | SZspecific | rs9289300 | -0.1383 | 0.1513 | 0.360833119 |
| EA | SZspecific | rs9320493 | -0.1399 | 0.1513 | 0.355150421 |
| EA | SZspecific | rs9342482 | -0.1374 | 0.1513 | 0.363843323 |
| EA | SZspecific | rs9349956 | -0.1299 | 0.1512 | 0.390040794 |
| EA | SZspecific | rs9372625 | -0.1345 | 0.1521 | 0.37645364 |
| EA | SZspecific | rs9384679 | -0.1387 | 0.1513 | 0.359084805 |
| EA | SZspecific | rs9386319 | -0.1451 | 0.1514 | 0.337860299 |
| EA | SZspecific | rs9436866 | -0.1474 | 0.1514 | 0.330265733 |
| EA | SZspecific | rs9503598 | -0.1424 | 0.1514 | 0.347041799 |
| EA | SZspecific | rs9529119 | -0.1354 | 0.1512 | 0.37025208 |
| EA | SZspecific | rs9556958 | -0.1492 | 0.1513 | 0.324099249 |
| EA | SZspecific | rs9616906 | -0.1455 | 0.1516 | 0.337235912 |
| EA | SZspecific | rs9679654 | -0.1440 | 0.1514 | 0.341674251 |
| EA | SZspecific | rs969512 | -0.1465 | 0.1514 | 0.333451774 |
| EA | SZspecific | rs9704097 | -0.1465 | 0.1514 | 0.333045329 |
| EA | SZspecific | rs9882532 | -0.1251 | 0.1503 | 0.405221652 |
| EA | SZspecific | rs9914918 | -0.1293 | 0.1506 | 0.390461401 |
| EA | SZspecific | rs9933256 | -0.1440 | 0.1514 | 0.341603979 |
| EA | SZspecific | rs9936270 | -0.1397 | 0.1514 | 0.356281569 |
| EA | SZspecific | rs9938678 | -0.1501 | 0.1513 | 0.320926267 |
| EA | SZspecific | rs9964724 | -0.1315 | 0.1516 | 0.385787489 |
| EA | SZspecific | rs9995567 | -0.1398 | 0.1513 | 0.355477917 |
| EA | SZspecific | All | -0.1434 | 0.1510 | 0.342290347 |
| EA | PSYshared | rs10073890 | 0.2855 | 0.1008 | 0.004614995 |
| EA | PSYshared | rs1008078 | 0.3020 | 0.1005 | 0.002641817 |
| EA | PSYshared | rs10189857 | 0.2763 | 0.1007 | 0.006088118 |
| EA | PSYshared | rs10205801 | 0.2803 | 0.1005 | 0.005284677 |
| EA | PSYshared | rs10215082 | 0.2770 | 0.1004 | 0.005813398 |

| | | | | | |
|---|---|---|---|---|---|
| EA | PSYshared | rs10240905 | 0.2809 | 0.1006 | 0.005227201 |
| EA | PSYshared | rs10456918 | 0.2854 | 0.1008 | 0.004629444 |
| EA | PSYshared | rs10460095 | 0.2874 | 0.1008 | 0.004351254 |
| EA | PSYshared | rs1051474 | 0.2849 | 0.1008 | 0.004694199 |
| EA | PSYshared | rs10760023 | 0.2911 | 0.1007 | 0.003831834 |
| EA | PSYshared | rs10765775 | 0.2913 | 0.1008 | 0.003865705 |
| EA | PSYshared | rs10772644 | 0.2829 | 0.1007 | 0.004949383 |
| EA | PSYshared | rs10773002 | 0.2769 | 0.1009 | 0.006060767 |
| EA | PSYshared | rs10856785 | 0.2868 | 0.1008 | 0.0044215 |
| EA | PSYshared | rs10862376 | 0.2957 | 0.1004 | 0.00323299 |
| EA | PSYshared | rs10875121 | 0.2876 | 0.1009 | 0.004360815 |
| EA | PSYshared | rs10887801 | 0.2914 | 0.1007 | 0.003793348 |
| EA | PSYshared | rs10940921 | 0.2806 | 0.1005 | 0.005257288 |
| EA | PSYshared | rs10963297 | 0.2971 | 0.1007 | 0.003171668 |
| EA | PSYshared | rs10994777 | 0.2883 | 0.1008 | 0.00423232 |
| EA | PSYshared | rs11023749 | 0.2859 | 0.1008 | 0.004551999 |
| EA | PSYshared | rs1105307 | 0.2864 | 0.1008 | 0.004481035 |
| EA | PSYshared | rs1106090 | 0.2819 | 0.1007 | 0.005107006 |
| EA | PSYshared | rs11081529 | 0.2904 | 0.1008 | 0.003948167 |
| EA | PSYshared | rs11123818 | 0.2905 | 0.1011 | 0.00407166 |
| EA | PSYshared | rs111821073 | 0.2855 | 0.1008 | 0.004607743 |
| EA | PSYshared | rs11222609 | 0.2871 | 0.1008 | 0.004394836 |
| EA | PSYshared | rs112687095 | 0.2865 | 0.1008 | 0.004457699 |
| EA | PSYshared | rs112806496 | 0.2891 | 0.1007 | 0.004106182 |
| EA | PSYshared | rs113182709 | 0.2854 | 0.1007 | 0.00461747 |
| EA | PSYshared | rs113520408 | 0.2929 | 0.1006 | 0.0036088 |
| EA | PSYshared | rs113615161 | 0.2869 | 0.1008 | 0.00441151 |
| EA | PSYshared | rs1143770 | 0.2878 | 0.1008 | 0.004297584 |
| EA | PSYshared | rs115000530 | 0.2937 | 0.1007 | 0.003527092 |
| EA | PSYshared | rs11601122 | 0.2885 | 0.1009 | 0.004237557 |
| EA | PSYshared | rs11620355 | 0.2917 | 0.1006 | 0.003734925 |
| EA | PSYshared | rs11627087 | 0.2883 | 0.1007 | 0.004219949 |
| EA | PSYshared | rs11635092 | 0.2792 | 0.1005 | 0.005474658 |
| EA | PSYshared | rs11657342 | 0.2821 | 0.1007 | 0.005080513 |
| EA | PSYshared | rs11663602 | 0.2897 | 0.1008 | 0.004037341 |
| EA | PSYshared | rs11678980 | 0.2968 | 0.1007 | 0.003213864 |
| EA | PSYshared | rs11681861 | 0.2798 | 0.1003 | 0.005287112 |
| EA | PSYshared | rs11694904 | 0.2845 | 0.1008 | 0.004755942 |
| EA | PSYshared | rs11732657 | 0.2872 | 0.1008 | 0.004383673 |
| EA | PSYshared | rs117468730 | 0.2878 | 0.1008 | 0.004294628 |
| EA | PSYshared | rs11752914 | 0.2872 | 0.1008 | 0.004364855 |
| EA | PSYshared | rs11772580 | 0.2886 | 0.1008 | 0.004180314 |
| EA | PSYshared | rs117799466 | 0.2955 | 0.1003 | 0.003230559 |
| EA | PSYshared | rs11871429 | 0.2931 | 0.1006 | 0.003589497 |
| EA | PSYshared | rs12028010 | 0.3026 | 0.1001 | 0.002498636 |
| EA | PSYshared | rs12332731 | 0.2857 | 0.1008 | 0.004579602 |
| EA | PSYshared | rs12375949 | 0.2826 | 0.1008 | 0.005065346 |
| EA | PSYshared | rs12468040 | 0.2977 | 0.1005 | 0.003043224 |
| EA | PSYshared | rs12503522 | 0.2882 | 0.1008 | 0.004233735 |
| EA | PSYshared | rs12519073 | 0.2895 | 0.1007 | 0.004059351 |
| EA | PSYshared | rs12574281 | 0.2845 | 0.1007 | 0.004734647 |
| EA | PSYshared | rs12602286 | 0.2846 | 0.1008 | 0.004743345 |
| EA | PSYshared | rs12643771 | 0.2890 | 0.1009 | 0.004165938 |
| EA | PSYshared | rs12647336 | 0.2865 | 0.1008 | 0.004462001 |
| EA | PSYshared | rs12655753 | 0.2881 | 0.1008 | 0.004250091 |
| EA | PSYshared | rs12682775 | 0.2838 | 0.1007 | 0.004828972 |
| EA | PSYshared | rs12804787 | 0.2942 | 0.1003 | 0.00335578 |
| EA | PSYshared | rs1291818 | 0.2821 | 0.1006 | 0.00506396 |
| EA | PSYshared | rs12940014 | 0.2908 | 0.1006 | 0.003854336 |

| | | | | | |
|---|---|---|---|---|---|
| EA | PSYshared | rs13010566 | 0.2857 | 0.1008 | 0.00458135 |
| EA | PSYshared | rs13029509 | 0.2898 | 0.1007 | 0.004017679 |
| EA | PSYshared | rs13090388 | 0.2826 | 0.1015 | 0.005352526 |
| EA | PSYshared | rs13141210 | 0.2910 | 0.1008 | 0.003898637 |
| EA | PSYshared | rs13145650 | 0.2784 | 0.1003 | 0.005512849 |
| EA | PSYshared | rs1334297 | 0.3054 | 0.1007 | 0.002432117 |
| EA | PSYshared | rs13422673 | 0.2840 | 0.1008 | 0.004828427 |
| EA | PSYshared | rs13428598 | 0.2982 | 0.1006 | 0.003039141 |
| EA | PSYshared | rs1363862 | 0.2816 | 0.1006 | 0.005115264 |
| EA | PSYshared | rs1391438 | 0.2733 | 0.1004 | 0.006495487 |
| EA | PSYshared | rs1427298 | 0.2878 | 0.1008 | 0.004284096 |
| EA | PSYshared | rs1450782 | 0.2855 | 0.1007 | 0.004599095 |
| EA | PSYshared | rs152603 | 0.2900 | 0.1007 | 0.003973431 |
| EA | PSYshared | rs1558727 | 0.2816 | 0.1006 | 0.005117397 |
| EA | PSYshared | rs1566085 | 0.2927 | 0.1009 | 0.003723383 |
| EA | PSYshared | rs1569092 | 0.2905 | 0.1008 | 0.003952521 |
| EA | PSYshared | rs1582173 | 0.2849 | 0.1008 | 0.004690769 |
| EA | PSYshared | rs1584469 | 0.2964 | 0.1004 | 0.003150156 |
| EA | PSYshared | rs1592757 | 0.2758 | 0.0999 | 0.005749805 |
| EA | PSYshared | rs1595973 | 0.2847 | 0.1007 | 0.004712676 |
| EA | PSYshared | rs1618725 | 0.2853 | 0.1009 | 0.004688335 |
| EA | PSYshared | rs1620977 | 0.2720 | 0.1005 | 0.006781132 |
| EA | PSYshared | rs1671770 | 0.2800 | 0.1004 | 0.005302902 |
| EA | PSYshared | rs16846463 | 0.2876 | 0.1009 | 0.004354447 |
| EA | PSYshared | rs16854920 | 0.2925 | 0.1005 | 0.003608964 |
| EA | PSYshared | rs1689510 | 0.2969 | 0.1007 | 0.003209406 |
| EA | PSYshared | rs16995054 | 0.2810 | 0.1006 | 0.005228081 |
| EA | PSYshared | rs17048855 | 0.2912 | 0.1007 | 0.003826951 |
| EA | PSYshared | rs17126938 | 0.2727 | 0.0995 | 0.006111627 |
| EA | PSYshared | rs17425572 | 0.2742 | 0.1000 | 0.00612094 |
| EA | PSYshared | rs17489649 | 0.2872 | 0.1009 | 0.00440442 |
| EA | PSYshared | rs175325 | 0.2839 | 0.1008 | 0.004839314 |
| EA | PSYshared | rs17551064 | 0.2841 | 0.1007 | 0.004809353 |
| EA | PSYshared | rs17563464 | 0.2860 | 0.1008 | 0.004545357 |
| EA | PSYshared | rs17565975 | 0.2814 | 0.1006 | 0.005167213 |
| EA | PSYshared | rs17598675 | 0.2820 | 0.1007 | 0.00509932 |
| EA | PSYshared | rs176218 | 0.2848 | 0.1009 | 0.004762631 |
| EA | PSYshared | rs1827540 | 0.2778 | 0.1003 | 0.005583089 |
| EA | PSYshared | rs1866823 | 0.2927 | 0.1005 | 0.003598027 |
| EA | PSYshared | rs1882273 | 0.2992 | 0.1000 | 0.002775097 |
| EA | PSYshared | rs192436652 | 0.2858 | 0.1008 | 0.004570714 |
| EA | PSYshared | rs1925576 | 0.2811 | 0.1005 | 0.005167295 |
| EA | PSYshared | rs1947114 | 0.2835 | 0.1007 | 0.004861264 |
| EA | PSYshared | rs1964927 | 0.2947 | 0.1007 | 0.003412474 |
| EA | PSYshared | rs2016392 | 0.2864 | 0.1008 | 0.004484917 |
| EA | PSYshared | rs2052285 | 0.2915 | 0.1007 | 0.003788226 |
| EA | PSYshared | rs2067854 | 0.2770 | 0.1003 | 0.005760978 |
| EA | PSYshared | rs2179152 | 0.2837 | 0.1008 | 0.004909873 |
| EA | PSYshared | rs2182505 | 0.2865 | 0.1008 | 0.004458824 |
| EA | PSYshared | rs225291 | 0.2932 | 0.1005 | 0.003523492 |
| EA | PSYshared | rs2256965 | 0.2807 | 0.1006 | 0.005253992 |
| EA | PSYshared | rs2283076 | 0.2918 | 0.1006 | 0.003716339 |
| EA | PSYshared | rs2287838 | 0.2861 | 0.1008 | 0.004541114 |
| EA | PSYshared | rs2302761 | 0.2960 | 0.1003 | 0.003177469 |
| EA | PSYshared | rs2347526 | 0.2914 | 0.1008 | 0.003833241 |
| EA | PSYshared | rs242093 | 0.2863 | 0.1008 | 0.004497127 |
| EA | PSYshared | rs2447535 | 0.2895 | 0.1008 | 0.004059604 |
| EA | PSYshared | rs2554835 | 0.2940 | 0.1004 | 0.003391401 |
| EA | PSYshared | rs2570497 | 0.2892 | 0.1008 | 0.004119679 |

| | | | | | |
|---|---|---|---|---|---|
| EA | PSYshared | rs2725370 | 0.2912 | 0.1008 | 0.003870874 |
| EA | PSYshared | rs277828 | 0.2915 | 0.1006 | 0.00375595 |
| EA | PSYshared | rs2819336 | 0.3000 | 0.1006 | 0.002855538 |
| EA | PSYshared | rs2820314 | 0.2829 | 0.1007 | 0.004942772 |
| EA | PSYshared | rs28373063 | 0.2916 | 0.1006 | 0.003756881 |
| EA | PSYshared | rs28513670 | 0.2901 | 0.1008 | 0.003981009 |
| EA | PSYshared | rs2885198 | 0.2906 | 0.1007 | 0.003894102 |
| EA | PSYshared | rs2901616 | 0.2826 | 0.1006 | 0.004964533 |
| EA | PSYshared | rs2905426 | 0.2953 | 0.1003 | 0.0032332 |
| EA | PSYshared | rs2923431 | 0.2806 | 0.1005 | 0.005252885 |
| EA | PSYshared | rs2971970 | 0.2808 | 0.1007 | 0.00530284 |
| EA | PSYshared | rs2998315 | 0.2895 | 0.1008 | 0.004086241 |
| EA | PSYshared | rs3013014 | 0.2828 | 0.1006 | 0.004954162 |
| EA | PSYshared | rs301800 | 0.2876 | 0.1008 | 0.004330922 |
| EA | PSYshared | rs3026996 | 0.2850 | 0.1008 | 0.004705242 |
| EA | PSYshared | rs31940 | 0.2825 | 0.1007 | 0.005002221 |
| EA | PSYshared | rs337637 | 0.2873 | 0.1008 | 0.004365152 |
| EA | PSYshared | rs34316 | 0.2851 | 0.1011 | 0.004804699 |
| EA | PSYshared | rs34394051 | 0.2927 | 0.1005 | 0.003595683 |
| EA | PSYshared | rs34485537 | 0.2879 | 0.1008 | 0.004283806 |
| EA | PSYshared | rs34853711 | 0.2876 | 0.1009 | 0.00434695 |
| EA | PSYshared | rs35039375 | 0.2900 | 0.1008 | 0.004006354 |
| EA | PSYshared | rs35309068 | 0.2822 | 0.1008 | 0.005105084 |
| EA | PSYshared | rs35316276 | 0.2866 | 0.1008 | 0.004458556 |
| EA | PSYshared | rs35417702 | 0.2857 | 0.1009 | 0.004628451 |
| EA | PSYshared | rs35475880 | 0.2864 | 0.1008 | 0.004505576 |
| EA | PSYshared | rs35532491 | 0.2909 | 0.1007 | 0.003874799 |
| EA | PSYshared | rs36083520 | 0.2761 | 0.1003 | 0.005895815 |
| EA | PSYshared | rs36119825 | 0.2909 | 0.1007 | 0.00385926 |
| EA | PSYshared | rs363096 | 0.2969 | 0.1005 | 0.003137175 |
| EA | PSYshared | rs3747631 | 0.3033 | 0.1005 | 0.00254772 |
| EA | PSYshared | rs3788556 | 0.2911 | 0.1007 | 0.003842349 |
| EA | PSYshared | rs3800546 | 0.2851 | 0.1008 | 0.004668575 |
| EA | PSYshared | rs3809634 | 0.2885 | 0.1007 | 0.004185132 |
| EA | PSYshared | rs3890802 | 0.2761 | 0.0999 | 0.00571758 |
| EA | PSYshared | rs3897821 | 0.2762 | 0.1005 | 0.005986427 |
| EA | PSYshared | rs406413 | 0.2962 | 0.1006 | 0.003235188 |
| EA | PSYshared | rs4073894 | 0.2779 | 0.1004 | 0.005651125 |
| EA | PSYshared | rs4328757 | 0.2668 | 0.0981 | 0.006513371 |
| EA | PSYshared | rs4352658 | 0.2878 | 0.1008 | 0.004309205 |
| EA | PSYshared | rs4369924 | 0.2826 | 0.1006 | 0.004981096 |
| EA | PSYshared | rs4382592 | 0.2927 | 0.1008 | 0.003694639 |
| EA | PSYshared | rs4384309 | 0.2914 | 0.1007 | 0.003793986 |
| EA | PSYshared | rs4442732 | 0.2862 | 0.1008 | 0.004517082 |
| EA | PSYshared | rs4667029 | 0.2805 | 0.1004 | 0.005218264 |
| EA | PSYshared | rs4700393 | 0.2986 | 0.1010 | 0.003099534 |
| EA | PSYshared | rs4726070 | 0.2836 | 0.1008 | 0.00488824 |
| EA | PSYshared | rs4733264 | 0.2945 | 0.1003 | 0.003328845 |
| EA | PSYshared | rs4757957 | 0.2864 | 0.1009 | 0.004515708 |
| EA | PSYshared | rs4766424 | 0.2862 | 0.1007 | 0.00449616 |
| EA | PSYshared | rs4778058 | 0.2891 | 0.1007 | 0.004110339 |
| EA | PSYshared | rs4787457 | 0.2956 | 0.1008 | 0.003367718 |
| EA | PSYshared | rs4810227 | 0.2909 | 0.1008 | 0.003896567 |
| EA | PSYshared | rs4839155 | 0.2830 | 0.1007 | 0.004940935 |
| EA | PSYshared | rs4846724 | 0.2872 | 0.1008 | 0.004378129 |
| EA | PSYshared | rs4870482 | 0.2910 | 0.1006 | 0.003841781 |
| EA | PSYshared | rs4888746 | 0.2878 | 0.1007 | 0.004284518 |
| EA | PSYshared | rs4895650 | 0.2879 | 0.1008 | 0.004274285 |
| EA | PSYshared | rs4945424 | 0.2879 | 0.1008 | 0.004277729 |

| | | | | | |
|---|---|---|---|---|---|
| EA | PSYshared | rs4964046 | 0.2811 | 0.1005 | 0.005175286 |
| EA | PSYshared | rs4972400 | 0.2830 | 0.1007 | 0.004943309 |
| EA | PSYshared | rs4984541 | 0.2886 | 0.1007 | 0.00417078 |
| EA | PSYshared | rs532799 | 0.2894 | 0.1007 | 0.004070121 |
| EA | PSYshared | rs535307 | 0.2819 | 0.1005 | 0.005039861 |
| EA | PSYshared | rs55736314 | 0.2891 | 0.1009 | 0.004161933 |
| EA | PSYshared | rs55771711 | 0.2936 | 0.1007 | 0.003555247 |
| EA | PSYshared | rs56391344 | 0.2853 | 0.1009 | 0.004673792 |
| EA | PSYshared | rs575113 | 0.2783 | 0.1004 | 0.005588428 |
| EA | PSYshared | rs59123361 | 0.2950 | 0.1005 | 0.003344184 |
| EA | PSYshared | rs59480703 | 0.2859 | 0.1008 | 0.004540189 |
| EA | PSYshared | rs60904894 | 0.2882 | 0.1008 | 0.004230303 |
| EA | PSYshared | rs6122735 | 0.2933 | 0.1005 | 0.003521591 |
| EA | PSYshared | rs6123924 | 0.2818 | 0.1006 | 0.005105772 |
| EA | PSYshared | rs613872 | 0.2856 | 0.1008 | 0.0046295 |
| EA | PSYshared | rs62097985 | 0.2970 | 0.1004 | 0.003104362 |
| EA | PSYshared | rs62103084 | 0.2904 | 0.1007 | 0.003930953 |
| EA | PSYshared | rs62157915 | 0.2876 | 0.1008 | 0.004314792 |
| EA | PSYshared | rs62183776 | 0.2896 | 0.1007 | 0.004039223 |
| EA | PSYshared | rs62184480 | 0.2901 | 0.1008 | 0.004016907 |
| EA | PSYshared | rs622169 | 0.2862 | 0.1007 | 0.004500344 |
| EA | PSYshared | rs62439690 | 0.2874 | 0.1008 | 0.004338224 |
| EA | PSYshared | rs62444881 | 0.2720 | 0.1002 | 0.006618452 |
| EA | PSYshared | rs6493265 | 0.2926 | 0.1008 | 0.003689211 |
| EA | PSYshared | rs6513959 | 0.2692 | 0.0988 | 0.006459359 |
| EA | PSYshared | rs6557171 | 0.2956 | 0.1007 | 0.003322092 |
| EA | PSYshared | rs663234 | 0.2835 | 0.1007 | 0.004858949 |
| EA | PSYshared | rs66568921 | 0.2914 | 0.1009 | 0.003857232 |
| EA | PSYshared | rs6731373 | 0.2810 | 0.1006 | 0.005229986 |
| EA | PSYshared | rs6731967 | 0.2895 | 0.1007 | 0.004052507 |
| EA | PSYshared | rs67885444 | 0.2880 | 0.1008 | 0.004264211 |
| EA | PSYshared | rs67890737 | 0.2836 | 0.1007 | 0.004865784 |
| EA | PSYshared | rs6803651 | 0.2861 | 0.1008 | 0.004529713 |
| EA | PSYshared | rs6805241 | 0.2832 | 0.1007 | 0.004937908 |
| EA | PSYshared | rs6938002 | 0.2864 | 0.1008 | 0.004482884 |
| EA | PSYshared | rs6959891 | 0.2829 | 0.1007 | 0.004944498 |
| EA | PSYshared | rs7012546 | 0.2832 | 0.1007 | 0.004902797 |
| EA | PSYshared | rs7016302 | 0.2936 | 0.1004 | 0.00345462 |
| EA | PSYshared | rs702606 | 0.2851 | 0.1007 | 0.004648407 |
| EA | PSYshared | rs7029718 | 0.2661 | 0.1008 | 0.008292328 |
| EA | PSYshared | rs7031698 | 0.2934 | 0.1005 | 0.003506187 |
| EA | PSYshared | rs710629 | 0.2839 | 0.1007 | 0.004808866 |
| EA | PSYshared | rs71646142 | 0.2890 | 0.1007 | 0.004125423 |
| EA | PSYshared | rs7233920 | 0.2851 | 0.1008 | 0.004662762 |
| EA | PSYshared | rs72486027 | 0.2819 | 0.1005 | 0.005044712 |
| EA | PSYshared | rs7257460 | 0.2897 | 0.1007 | 0.004030996 |
| EA | PSYshared | rs7278859 | 0.2873 | 0.1008 | 0.004351743 |
| EA | PSYshared | rs72807818 | 0.2944 | 0.1006 | 0.003431861 |
| EA | PSYshared | rs72828517 | 0.2910 | 0.1008 | 0.003899092 |
| EA | PSYshared | rs72840994 | 0.2841 | 0.1007 | 0.004782846 |
| EA | PSYshared | rs730384 | 0.2899 | 0.1007 | 0.003997459 |
| EA | PSYshared | rs7315713 | 0.2908 | 0.1006 | 0.003864212 |
| EA | PSYshared | rs73301698 | 0.2862 | 0.1008 | 0.004509158 |
| EA | PSYshared | rs7332724 | 0.2939 | 0.1005 | 0.003447712 |
| EA | PSYshared | rs73344830 | 0.2820 | 0.1009 | 0.005208469 |
| EA | PSYshared | rs736282 | 0.2894 | 0.1008 | 0.004078145 |
| EA | PSYshared | rs73874335 | 0.2851 | 0.1007 | 0.004654366 |
| EA | PSYshared | rs743316 | 0.2921 | 0.1006 | 0.003681259 |
| EA | PSYshared | rs74643044 | 0.2873 | 0.1008 | 0.004358886 |

| | | | | | |
|---|---|---|---|---|---|
| EA | PSYshared | rs74701752 | 0.2915 | 0.1006 | 0.003759032 |
| EA | PSYshared | rs74998289 | 0.2955 | 0.1007 | 0.003339423 |
| EA | PSYshared | rs7543462 | 0.2857 | 0.1007 | 0.004565605 |
| EA | PSYshared | rs7594904 | 0.2913 | 0.1006 | 0.003788842 |
| EA | PSYshared | rs7603132 | 0.2955 | 0.1003 | 0.003214955 |
| EA | PSYshared | rs76076331 | 0.2928 | 0.1007 | 0.003645297 |
| EA | PSYshared | rs7650602 | 0.2832 | 0.1006 | 0.004891651 |
| EA | PSYshared | rs76608582 | 0.2857 | 0.1008 | 0.004569592 |
| EA | PSYshared | rs77025239 | 0.2832 | 0.1007 | 0.004916514 |
| EA | PSYshared | rs77128898 | 0.2928 | 0.1005 | 0.00358884 |
| EA | PSYshared | rs77702622 | 0.2867 | 0.1008 | 0.004460836 |
| EA | PSYshared | rs77719387 | 0.2872 | 0.1008 | 0.004368419 |
| EA | PSYshared | rs7773815 | 0.2896 | 0.1007 | 0.004039497 |
| EA | PSYshared | rs77835879 | 0.2829 | 0.1006 | 0.00492373 |
| EA | PSYshared | rs7796203 | 0.2806 | 0.1005 | 0.005251604 |
| EA | PSYshared | rs7803932 | 0.2920 | 0.1006 | 0.003718897 |
| EA | PSYshared | rs7808399 | 0.2856 | 0.1008 | 0.004595449 |
| EA | PSYshared | rs7833201 | 0.2891 | 0.1007 | 0.004102523 |
| EA | PSYshared | rs7863447 | 0.2927 | 0.1007 | 0.00365398 |
| EA | PSYshared | rs7864396 | 0.2949 | 0.1003 | 0.003279028 |
| EA | PSYshared | rs78721320 | 0.2888 | 0.1007 | 0.004141433 |
| EA | PSYshared | rs790647 | 0.2891 | 0.1008 | 0.004142261 |
| EA | PSYshared | rs7924036 | 0.2919 | 0.1009 | 0.00379474 |
| EA | PSYshared | rs79265434 | 0.2662 | 0.0996 | 0.007507796 |
| EA | PSYshared | rs79269403 | 0.2916 | 0.1007 | 0.003788732 |
| EA | PSYshared | rs7928622 | 0.2887 | 0.1007 | 0.004155879 |
| EA | PSYshared | rs795230 | 0.2857 | 0.1007 | 0.004573184 |
| EA | PSYshared | rs79523955 | 0.2918 | 0.1006 | 0.003738187 |
| EA | PSYshared | rs7977614 | 0.2886 | 0.1008 | 0.004196812 |
| EA | PSYshared | rs7993663 | 0.2947 | 0.1005 | 0.003363083 |
| EA | PSYshared | rs8008382 | 0.2840 | 0.1007 | 0.004811252 |
| EA | PSYshared | rs80171383 | 0.2939 | 0.1004 | 0.003435635 |
| EA | PSYshared | rs8020034 | 0.2907 | 0.1008 | 0.003937938 |
| EA | PSYshared | rs818415 | 0.2829 | 0.1006 | 0.004934678 |
| EA | PSYshared | rs837080 | 0.2909 | 0.1007 | 0.003876252 |
| EA | PSYshared | rs892612 | 0.2846 | 0.1007 | 0.004734904 |
| EA | PSYshared | rs894067 | 0.2842 | 0.1007 | 0.004778905 |
| EA | PSYshared | rs9289300 | 0.2891 | 0.1008 | 0.004120767 |
| EA | PSYshared | rs9320493 | 0.2897 | 0.1007 | 0.004027643 |
| EA | PSYshared | rs9342482 | 0.2863 | 0.1008 | 0.00450595 |
| EA | PSYshared | rs9349956 | 0.2757 | 0.1004 | 0.006049441 |
| EA | PSYshared | rs9372625 | 0.2618 | 0.1005 | 0.009164812 |
| EA | PSYshared | rs9384679 | 0.2904 | 0.1007 | 0.003915085 |
| EA | PSYshared | rs9386319 | 0.2893 | 0.1007 | 0.004075682 |
| EA | PSYshared | rs9436866 | 0.2864 | 0.1008 | 0.004490243 |
| EA | PSYshared | rs9503598 | 0.2903 | 0.1007 | 0.003944192 |
| EA | PSYshared | rs9529119 | 0.2782 | 0.1003 | 0.005547982 |
| EA | PSYshared | rs9556958 | 0.2851 | 0.1008 | 0.004658558 |
| EA | PSYshared | rs9616906 | 0.2741 | 0.1004 | 0.006316513 |
| EA | PSYshared | rs9679654 | 0.2843 | 0.1007 | 0.004767126 |
| EA | PSYshared | rs969512 | 0.2833 | 0.1008 | 0.004925919 |
| EA | PSYshared | rs9704097 | 0.2875 | 0.1008 | 0.00433546 |
| EA | PSYshared | rs9882532 | 0.2944 | 0.1005 | 0.003406561 |
| EA | PSYshared | rs9914918 | 0.2860 | 0.1008 | 0.004538701 |
| EA | PSYshared | rs9933256 | 0.2849 | 0.1007 | 0.004691245 |
| EA | PSYshared | rs9936270 | 0.2788 | 0.1005 | 0.005511459 |
| EA | PSYshared | rs9938678 | 0.2929 | 0.1006 | 0.003595461 |
| EA | PSYshared | rs9964724 | 0.2889 | 0.1011 | 0.004246673 |
| EA | PSYshared | rs9995567 | 0.2879 | 0.1008 | 0.004273423 |

| | | | | | |
|---|---|---|---|---|---|
| EA | PSYshared | All | 0.2871 | 0.1005 | 0.004277761 |
| IQ | Schizophrenia | rs10189857 | -0.5339 | 0.1440 | 0.000208214 |
| IQ | Schizophrenia | rs10189912 | -0.4703 | 0.1547 | 0.002355615 |
| IQ | Schizophrenia | rs10779271 | -0.5026 | 0.1505 | 0.000837248 |
| IQ | Schizophrenia | rs10917152 | -0.4553 | 0.1527 | 0.002866551 |
| IQ | Schizophrenia | rs11210871 | -0.4295 | 0.1455 | 0.003161888 |
| IQ | Schizophrenia | rs112780312 | -0.4486 | 0.1515 | 0.003065365 |
| IQ | Schizophrenia | rs11678106 | -0.4912 | 0.1532 | 0.001342268 |
| IQ | Schizophrenia | rs11720523 | -0.4326 | 0.1486 | 0.003591726 |
| IQ | Schizophrenia | rs11898362 | -0.4439 | 0.1504 | 0.003167874 |
| IQ | Schizophrenia | rs12026245 | -0.4778 | 0.1546 | 0.00199627 |
| IQ | Schizophrenia | rs12035012 | -0.4810 | 0.1554 | 0.001967522 |
| IQ | Schizophrenia | rs12470949 | -0.4682 | 0.1540 | 0.002369556 |
| IQ | Schizophrenia | rs12646225 | -0.4944 | 0.1523 | 0.001171333 |
| IQ | Schizophrenia | rs13024268 | -0.4621 | 0.1536 | 0.00262454 |
| IQ | Schizophrenia | rs13071190 | -0.4899 | 0.1536 | 0.001428064 |
| IQ | Schizophrenia | rs13395129 | -0.4969 | 0.1522 | 0.001092858 |
| IQ | Schizophrenia | rs144246 | -0.4729 | 0.1541 | 0.002152222 |
| IQ | Schizophrenia | rs1473474 | -0.4677 | 0.1540 | 0.002386118 |
| IQ | Schizophrenia | rs1589652 | -0.4799 | 0.1544 | 0.00187877 |
| IQ | Schizophrenia | rs1831539 | -0.4976 | 0.1526 | 0.001114492 |
| IQ | Schizophrenia | rs2007176 | -0.4576 | 0.1528 | 0.002745435 |
| IQ | Schizophrenia | rs2268894 | -0.4323 | 0.1506 | 0.004110011 |
| IQ | Schizophrenia | rs2309812 | -0.4888 | 0.1550 | 0.00161386 |
| IQ | Schizophrenia | rs2352974 | -0.5391 | 0.1530 | 0.000426 |
| IQ | Schizophrenia | rs2420551 | -0.4928 | 0.1533 | 0.001309034 |
| IQ | Schizophrenia | rs2558096 | -0.4473 | 0.1508 | 0.003009133 |
| IQ | Schizophrenia | rs2678210 | -0.4689 | 0.1544 | 0.002383749 |
| IQ | Schizophrenia | rs2955280 | -0.4651 | 0.1536 | 0.002466817 |
| IQ | Schizophrenia | rs297578 | -0.4715 | 0.1543 | 0.002247855 |
| IQ | Schizophrenia | rs3128341 | -0.4912 | 0.1559 | 0.001629566 |
| IQ | Schizophrenia | rs34811474 | -0.4600 | 0.1549 | 0.002980767 |
| IQ | Schizophrenia | rs35731967 | -0.4913 | 0.1533 | 0.001354317 |
| IQ | Schizophrenia | rs3860537 | -0.4732 | 0.1542 | 0.002147585 |
| IQ | Schizophrenia | rs41293013 | -0.4770 | 0.1548 | 0.002056633 |
| IQ | Schizophrenia | rs4484297 | -0.4740 | 0.1543 | 0.00212395 |
| IQ | Schizophrenia | rs4852252 | -0.4416 | 0.1523 | 0.003738783 |
| IQ | Schizophrenia | rs58593843 | -0.4737 | 0.1541 | 0.002111932 |
| IQ | Schizophrenia | rs59142272 | -0.4934 | 0.1532 | 0.001277236 |
| IQ | Schizophrenia | rs600806 | -0.4938 | 0.1531 | 0.001259147 |
| IQ | Schizophrenia | rs60262711 | -0.4724 | 0.1542 | 0.002184276 |
| IQ | Schizophrenia | rs62181012 | -0.4913 | 0.1535 | 0.001370136 |
| IQ | Schizophrenia | rs62198803 | -0.4608 | 0.1535 | 0.002688336 |
| IQ | Schizophrenia | rs6432749 | -0.4740 | 0.1542 | 0.002119089 |
| IQ | Schizophrenia | rs6550835 | -0.4561 | 0.1550 | 0.003253442 |
| IQ | Schizophrenia | rs6668048 | -0.4844 | 0.1550 | 0.001784769 |
| IQ | Schizophrenia | rs6770622 | -0.4847 | 0.1542 | 0.00166712 |
| IQ | Schizophrenia | rs7518151 | -0.4702 | 0.1541 | 0.002278592 |
| IQ | Schizophrenia | rs7573001 | -0.4516 | 0.1518 | 0.002931577 |
| IQ | Schizophrenia | rs7640196 | -0.4619 | 0.1533 | 0.002581279 |
| IQ | Schizophrenia | rs7652296 | -0.4884 | 0.1536 | 0.001473103 |
| IQ | Schizophrenia | rs967569 | -0.5011 | 0.1519 | 0.000971112 |
| IQ | Schizophrenia | All | -0.4750 | 0.1515 | 0.001721563 |
| IQ | Bipolar | rs10189857 | -0.1689 | 0.1379 | 0.22060506 |
| IQ | Bipolar | rs10189912 | -0.1167 | 0.1412 | 0.408697606 |
| IQ | Bipolar | rs10779271 | -0.1389 | 0.1415 | 0.326118728 |
| IQ | Bipolar | rs10917152 | -0.1257 | 0.1416 | 0.374641492 |
| IQ | Bipolar | rs112780312 | -0.1349 | 0.1418 | 0.34145712 |
| IQ | Bipolar | rs11678106 | -0.1150 | 0.1403 | 0.412458739 |

| | | | | | |
|---|---|---|---|---|---|
| IQ | Bipolar | rs11720523 | -0.1340 | 0.1421 | 0.345665033 |
| IQ | Bipolar | rs11898362 | -0.0859 | 0.1316 | 0.514020431 |
| IQ | Bipolar | rs12026245 | -0.1194 | 0.1414 | 0.398332063 |
| IQ | Bipolar | rs12035012 | -0.0885 | 0.1384 | 0.52244335 |
| IQ | Bipolar | rs12470949 | -0.1396 | 0.1415 | 0.323971195 |
| IQ | Bipolar | rs12646225 | -0.1473 | 0.1405 | 0.294570888 |
| IQ | Bipolar | rs13024268 | -0.1223 | 0.1412 | 0.386671726 |
| IQ | Bipolar | rs13071190 | -0.1527 | 0.1403 | 0.276330459 |
| IQ | Bipolar | rs144246 | -0.1361 | 0.1416 | 0.336433377 |
| IQ | Bipolar | rs1473474 | -0.1175 | 0.1405 | 0.403030222 |
| IQ | Bipolar | rs1589652 | -0.1471 | 0.1411 | 0.297359353 |
| IQ | Bipolar | rs1831539 | -0.1792 | 0.1330 | 0.177832763 |
| IQ | Bipolar | rs2007176 | -0.1110 | 0.1392 | 0.425356422 |
| IQ | Bipolar | rs2268894 | -0.1078 | 0.1409 | 0.444320655 |
| IQ | Bipolar | rs2309812 | -0.1361 | 0.1430 | 0.340906507 |
| IQ | Bipolar | rs2352974 | -0.1284 | 0.1453 | 0.376907724 |
| IQ | Bipolar | rs2558096 | -0.1219 | 0.1411 | 0.387432845 |
| IQ | Bipolar | rs2678210 | -0.1499 | 0.1409 | 0.287211764 |
| IQ | Bipolar | rs2955280 | -0.1392 | 0.1414 | 0.324762757 |
| IQ | Bipolar | rs297578 | -0.1195 | 0.1411 | 0.396887923 |
| IQ | Bipolar | rs3128341 | -0.1487 | 0.1435 | 0.299952451 |
| IQ | Bipolar | rs34811474 | -0.1005 | 0.1406 | 0.474977759 |
| IQ | Bipolar | rs35731967 | -0.1377 | 0.1418 | 0.331366285 |
| IQ | Bipolar | rs3860537 | -0.1306 | 0.1417 | 0.356771194 |
| IQ | Bipolar | rs4852252 | -0.1420 | 0.1424 | 0.318808934 |
| IQ | Bipolar | rs58593843 | -0.1470 | 0.1404 | 0.295143159 |
| IQ | Bipolar | rs59142272 | -0.1621 | 0.1384 | 0.241475105 |
| IQ | Bipolar | rs600806 | -0.1746 | 0.1346 | 0.194506952 |
| IQ | Bipolar | rs60262711 | -0.1429 | 0.1411 | 0.311076621 |
| IQ | Bipolar | rs62181012 | -0.1626 | 0.1382 | 0.239378925 |
| IQ | Bipolar | rs62198803 | -0.1287 | 0.1418 | 0.364057993 |
| IQ | Bipolar | rs6432749 | -0.1310 | 0.1417 | 0.355252647 |
| IQ | Bipolar | rs6550835 | -0.1302 | 0.1432 | 0.363518872 |
| IQ | Bipolar | rs6668048 | -0.0942 | 0.1388 | 0.497367126 |
| IQ | Bipolar | rs6770622 | -0.1364 | 0.1419 | 0.336443611 |
| IQ | Bipolar | rs7518151 | -0.1065 | 0.1382 | 0.441031613 |
| IQ | Bipolar | rs7640196 | -0.1250 | 0.1413 | 0.376412059 |
| IQ | Bipolar | rs7652296 | -0.1502 | 0.1405 | 0.284818279 |
| IQ | Bipolar | rs967569 | -0.1433 | 0.1415 | 0.311182452 |
| IQ | Bipolar | All | -0.1329 | 0.1389 | 0.338901099 |
| IQ | SZspecific | rs10189857 | -0.7081 | 0.2191 | 0.001230461 |
| IQ | SZspecific | rs10189912 | -0.6530 | 0.2274 | 0.004089428 |
| IQ | SZspecific | rs10779271 | -0.6860 | 0.2210 | 0.001907263 |
| IQ | SZspecific | rs10917152 | -0.6162 | 0.2249 | 0.006153938 |
| IQ | SZspecific | rs11210871 | -0.5949 | 0.2203 | 0.006924519 |
| IQ | SZspecific | rs112780312 | -0.5949 | 0.2205 | 0.006961964 |
| IQ | SZspecific | rs11678106 | -0.6911 | 0.2205 | 0.001718181 |
| IQ | SZspecific | rs11720523 | -0.5674 | 0.2141 | 0.008058241 |
| IQ | SZspecific | rs11898362 | -0.6396 | 0.2271 | 0.004847789 |
| IQ | SZspecific | rs12026245 | -0.6628 | 0.2266 | 0.003446529 |
| IQ | SZspecific | rs12035012 | -0.7013 | 0.2240 | 0.001745713 |
| IQ | SZspecific | rs12470949 | -0.6241 | 0.2258 | 0.005710076 |
| IQ | SZspecific | rs12646225 | -0.6616 | 0.2257 | 0.003376506 |
| IQ | SZspecific | rs13024268 | -0.6323 | 0.2266 | 0.005274157 |
| IQ | SZspecific | rs13071190 | -0.6478 | 0.2272 | 0.004355156 |
| IQ | SZspecific | rs13395129 | -0.6857 | 0.2218 | 0.001987148 |
| IQ | SZspecific | rs144246 | -0.6362 | 0.2266 | 0.004996552 |
| IQ | SZspecific | rs1473474 | -0.6474 | 0.2268 | 0.004317266 |
| IQ | SZspecific | rs1589652 | -0.6365 | 0.2271 | 0.005070294 |

| | | | | | |
|---|---|---|---|---|---|
| IQ | SZspecific | rs1831539 | -0.6320 | 0.2270 | 0.005361154 |
| IQ | SZspecific | rs2007176 | -0.6363 | 0.2267 | 0.005000967 |
| IQ | SZspecific | rs2268894 | -0.5960 | 0.2245 | 0.007940961 |
| IQ | SZspecific | rs2309812 | -0.6639 | 0.2281 | 0.003611094 |
| IQ | SZspecific | rs2352974 | -0.7604 | 0.2215 | 0.000598192 |
| IQ | SZspecific | rs2420551 | -0.6676 | 0.2259 | 0.003123476 |
| IQ | SZspecific | rs2558096 | -0.6069 | 0.2228 | 0.006446543 |
| IQ | SZspecific | rs2678210 | -0.6143 | 0.2252 | 0.006388572 |
| IQ | SZspecific | rs2955280 | -0.6191 | 0.2248 | 0.005878098 |
| IQ | SZspecific | rs297578 | -0.6513 | 0.2270 | 0.004108294 |
| IQ | SZspecific | rs3128341 | -0.6545 | 0.2300 | 0.004424269 |
| IQ | SZspecific | rs34811474 | -0.6499 | 0.2287 | 0.004484142 |
| IQ | SZspecific | rs35731967 | -0.6668 | 0.2257 | 0.003134556 |
| IQ | SZspecific | rs3860537 | -0.6428 | 0.2269 | 0.004612592 |
| IQ | SZspecific | rs4484297 | -0.6204 | 0.2254 | 0.005914003 |
| IQ | SZspecific | rs4852252 | -0.5745 | 0.2200 | 0.009029563 |
| IQ | SZspecific | rs58593843 | -0.6254 | 0.2256 | 0.005574662 |
| IQ | SZspecific | rs59142272 | -0.6439 | 0.2273 | 0.004609233 |
| IQ | SZspecific | rs600806 | -0.6313 | 0.2269 | 0.005390919 |
| IQ | SZspecific | rs60262711 | -0.6274 | 0.2260 | 0.005507439 |
| IQ | SZspecific | rs62181012 | -0.6391 | 0.2272 | 0.004910112 |
| IQ | SZspecific | rs62198803 | -0.6227 | 0.2259 | 0.005839138 |
| IQ | SZspecific | rs6432749 | -0.6435 | 0.2269 | 0.004572083 |
| IQ | SZspecific | rs6550835 | -0.6126 | 0.2279 | 0.007183897 |
| IQ | SZspecific | rs6668048 | -0.7012 | 0.2231 | 0.001672617 |
| IQ | SZspecific | rs6770622 | -0.6564 | 0.2269 | 0.003821426 |
| IQ | SZspecific | rs7518151 | -0.6637 | 0.2257 | 0.003271872 |
| IQ | SZspecific | rs7573001 | -0.6165 | 0.2247 | 0.006085601 |
| IQ | SZspecific | rs7640196 | -0.6288 | 0.2260 | 0.005403239 |
| IQ | SZspecific | rs7652296 | -0.6482 | 0.2270 | 0.004298163 |
| IQ | SZspecific | rs967569 | -0.6777 | 0.2243 | 0.002515568 |
| IQ | SZspecific | All | -0.6435 | 0.2229 | 0.003882597 |
| IQ | PSYshared | rs10189857 | -0.2363 | 0.1672 | 0.157684771 |
| IQ | PSYshared | rs10189912 | -0.1751 | 0.1711 | 0.306179648 |
| IQ | PSYshared | rs10779271 | -0.2010 | 0.1713 | 0.240663627 |
| IQ | PSYshared | rs10917152 | -0.1855 | 0.1714 | 0.279219029 |
| IQ | PSYshared | rs11210871 | -0.1600 | 0.1674 | 0.339154454 |
| IQ | PSYshared | rs112780312 | -0.1963 | 0.1716 | 0.252825536 |
| IQ | PSYshared | rs11678106 | -0.1729 | 0.1700 | 0.309153858 |
| IQ | PSYshared | rs11720523 | -0.1954 | 0.1720 | 0.256047344 |
| IQ | PSYshared | rs11898362 | -0.1388 | 0.1606 | 0.387184086 |
| IQ | PSYshared | rs12026245 | -0.1782 | 0.1713 | 0.297963812 |
| IQ | PSYshared | rs12035012 | -0.1424 | 0.1681 | 0.396841474 |
| IQ | PSYshared | rs12470949 | -0.2017 | 0.1713 | 0.238940709 |
| IQ | PSYshared | rs12646225 | -0.2107 | 0.1702 | 0.215596492 |
| IQ | PSYshared | rs13024268 | -0.1815 | 0.1711 | 0.288777419 |
| IQ | PSYshared | rs13071190 | -0.2172 | 0.1699 | 0.201142662 |
| IQ | PSYshared | rs13395129 | -0.1899 | 0.1716 | 0.268442049 |
| IQ | PSYshared | rs144246 | -0.1976 | 0.1714 | 0.248887628 |
| IQ | PSYshared | rs1473474 | -0.1758 | 0.1702 | 0.301708809 |
| IQ | PSYshared | rs1589652 | -0.2106 | 0.1708 | 0.217753057 |
| IQ | PSYshared | rs1831539 | -0.2482 | 0.1617 | 0.124899081 |
| IQ | PSYshared | rs2007176 | -0.1682 | 0.1689 | 0.319206147 |
| IQ | PSYshared | rs2268894 | -0.1648 | 0.1707 | 0.334470504 |
| IQ | PSYshared | rs2309812 | -0.1981 | 0.1729 | 0.251936159 |
| IQ | PSYshared | rs2352974 | -0.1898 | 0.1755 | 0.279636997 |
| IQ | PSYshared | rs2420551 | -0.2014 | 0.1716 | 0.240711333 |
| IQ | PSYshared | rs2558096 | -0.1811 | 0.1709 | 0.289394036 |
| IQ | PSYshared | rs2678210 | -0.2139 | 0.1706 | 0.209717166 |

| | | | | | |
|---|---|---|---|---|---|
| IQ | PSYshared | rs2955280 | -0.2013 | 0.1712 | 0.239607032 |
| IQ | PSYshared | rs297578 | -0.1783 | 0.1709 | 0.296852972 |
| IQ | PSYshared | rs3128341 | -0.2131 | 0.1735 | 0.219256317 |
| IQ | PSYshared | rs34811474 | -0.1564 | 0.1705 | 0.358957931 |
| IQ | PSYshared | rs35731967 | -0.1996 | 0.1716 | 0.244768005 |
| IQ | PSYshared | rs3860537 | -0.1912 | 0.1715 | 0.265053686 |
| IQ | PSYshared | rs4484297 | -0.2159 | 0.1695 | 0.202761072 |
| IQ | PSYshared | rs4852252 | -0.2048 | 0.1723 | 0.234536701 |
| IQ | PSYshared | rs58593843 | -0.2104 | 0.1701 | 0.216053947 |
| IQ | PSYshared | rs59142272 | -0.2282 | 0.1678 | 0.173812957 |
| IQ | PSYshared | rs600806 | -0.2429 | 0.1635 | 0.137559382 |
| IQ | PSYshared | rs60262711 | -0.2056 | 0.1708 | 0.228710537 |
| IQ | PSYshared | rs62181012 | -0.2288 | 0.1676 | 0.172177193 |
| IQ | PSYshared | rs62198803 | -0.1890 | 0.1716 | 0.270795842 |
| IQ | PSYshared | rs6432749 | -0.1917 | 0.1716 | 0.263834916 |
| IQ | PSYshared | rs6550835 | -0.1912 | 0.1733 | 0.269806715 |
| IQ | PSYshared | rs6668048 | -0.1490 | 0.1685 | 0.376712386 |
| IQ | PSYshared | rs6770622 | -0.1982 | 0.1718 | 0.248762854 |
| IQ | PSYshared | rs7518151 | -0.1629 | 0.1677 | 0.33141001 |
| IQ | PSYshared | rs7573001 | -0.1781 | 0.1706 | 0.29659017 |
| IQ | PSYshared | rs7640196 | -0.1846 | 0.1711 | 0.280684966 |
| IQ | PSYshared | rs7652296 | -0.2143 | 0.1701 | 0.207850437 |
| IQ | PSYshared | rs967569 | -0.2062 | 0.1713 | 0.228710968 |
| IQ | PSYshared | All | -0.1935 | 0.1685 | 0.250813298 |
